# Discovery of Desmuramylpeptide NOD2 Agonists with Single-Digit Nanomolar Potency

**DOI:** 10.1021/acsmedchemlett.2c00121

**Published:** 2022-07-18

**Authors:** Samo Guzelj, Špela Bizjak, Žiga Jakopin

**Affiliations:** Faculty of Pharmacy, University of Ljubljana, SI-1000 Ljubljana, Slovenia

**Keywords:** NOD2, desmuramylpeptide, immunostimulant, adamantane, PBMC cytotoxicity, K562

## Abstract

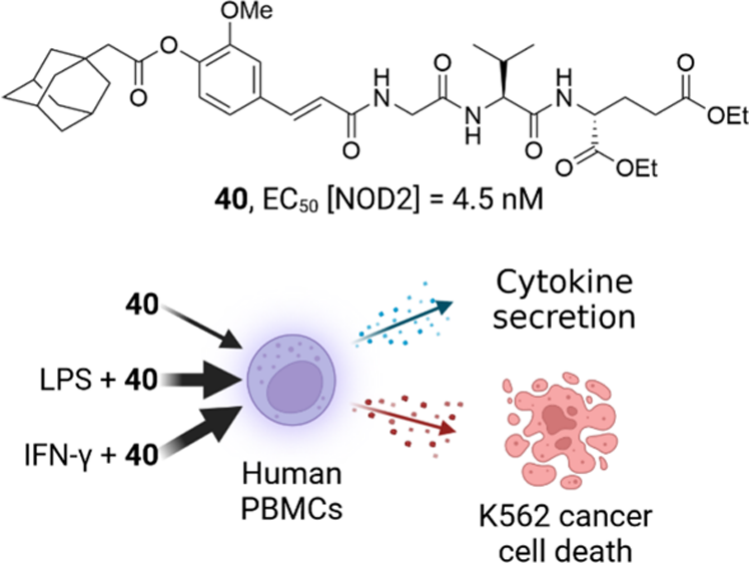

The innate immune receptor nucleotide-binding oligomerization-domain-containing protein 2 (NOD2) represents an important target for the development of structurally defined small molecule immunomodulatory compounds that have great potential to be used either as vaccine adjuvants or as general immunostimulatory agents. We report here the investigation of the structure–activity relationship of a series of novel desmuramylpeptide NOD2 agonists. Extensive exploration of chemical space culminated in the discovery of a lipophilic adamantane-moiety-featuring compound **40**, the first single-digit nanomolar and the most potent NOD2 agonist in its structural class to date. Moreover, **40** acted synergistically with lipopolysaccharide and interferon-γ to induce the production of cytokines in human peripheral blood mononuclear cells and enhance their nonspecific cytotoxic activity against K562 cancer cells. These findings provide initial insight into its immunostimulatory potential, especially when used in combination with other immunopotentiators.

Nucleotide-binding oligomerization-domain-containing protein 2 (NOD2) and its closely related partner NOD1 are cytosolic innate immune receptors that have evolved to recognize and respond to bacterial peptidoglycan fragments.^1^ Stimulation of NOD2 by its cognate ligands activates the nuclear factor κB (NF-κB) and mitogen-activated protein kinase downstream signaling pathways.^2^ The resulting proinflammatory signature, characterized by the production of cytokines, type I interferons (IFNs), nitric oxide, and reactive oxygen species, thus provides rapid protection against invading microbes while also augmenting antigen-specific adaptive immunity.^3^

The clinical utility of NOD2 agonists was first illustrated by the discovery that the adjuvanticity of Freund’s complete adjuvant was based on muramyl dipeptide (MDP), the smallest fragment of peptidoglycan still capable of activating NOD2.^4,5^ In addition to stimulating cytokine production, NOD2 agonists also trigger maturation and activation of dendritic cells as well as induce autophagy, which are highly desirable traits in vaccine adjuvant development.^6−8^ However, the use of NOD2 agonists is not limited to conventional prophylactic vaccines. For instance, engagement of NOD2 proved to be essential for antigen-specific mucosal and systemic responses of mucosal vaccines.^9,10^ The capacity of NOD2 agonists to enhance the antitumor activity of immune cells also underscores their potential in cancer immunotherapy.^11^ Moreover, consistent with its evolutionary role, activation of NOD2 provides protection against microbial infections and could be exploited in the treatment of acute infections.^12^ Interestingly, in contrast to the predominantly proinflammatory effects described above, sustained triggering of NOD2 also induces a switch in monocytes from the inflammatory Ly6C^hi^ to the patrolling Ly6C^low^ subset, which exerts a regulatory role and assists in tissue repair.^13^ The use of MDP has therefore also shown promise in mouse models of multiple sclerosis and Alzheimer’s disease, as these conditions are characterized by chronic inflammation.^14,15^

While pyrogenicity, rapid elimination, and metabolic instability preclude the use of MDP in the clinic, both the efficacy and safety profile can be improved by chemical modifications of the parent structure.^16^ Our previous efforts led to the discovery of desmuramylpeptides **1** and **2**, MDP analogues that carry a *trans*-feruloyl-glycine moiety as a replacement of the *N*-acetylmuramic acid moiety of MDP and exhibit potent NOD2 stimulatory activity in the low nanomolar range (Figure 1A).^17,18^ Here, we continue our systematic search for chemical modifications that would further improve their NOD2 agonistic activity. Our earlier work has shown that incorporation of amino acids with bulkier hydrophobic side chains, namely l-phenylalanine and l-valine, results in significantly improved NOD2 activity compared to desmuramylpeptides featuring the less bulky l-alanine and the more hydrophilic l-serine and l-threonine.^17,18^ However, it is important to note that, similar to the present study, the results were obtained in cellular assays in which both ligand binding affinity as well as cell membrane permeability play a role. In fact, previous studies suggest that desmuramylpeptides likely enter the cell through passive membrane penetration.^19^ Consequently, the increased NOD2 activity of desmuramylpeptides equipped with lipophilic side chains could be attributed to the occupation of a potential hydrophobic side pocket, enhanced membrane permeability, or both. In the present study, we first extend the analysis of structure–activity relationship (SAR) to unnatural amino acids, namely, the hydrophobic l-cyclohexlyalanine, l-homophenylalanine, and (*S*)-adamantylglycine, whereas l-pyridylalanine was investigated due to previous reports of its capacity to substitute l-phenylalanine and improve solubility while retaining bioactivity.^20^

**Figure 1:**
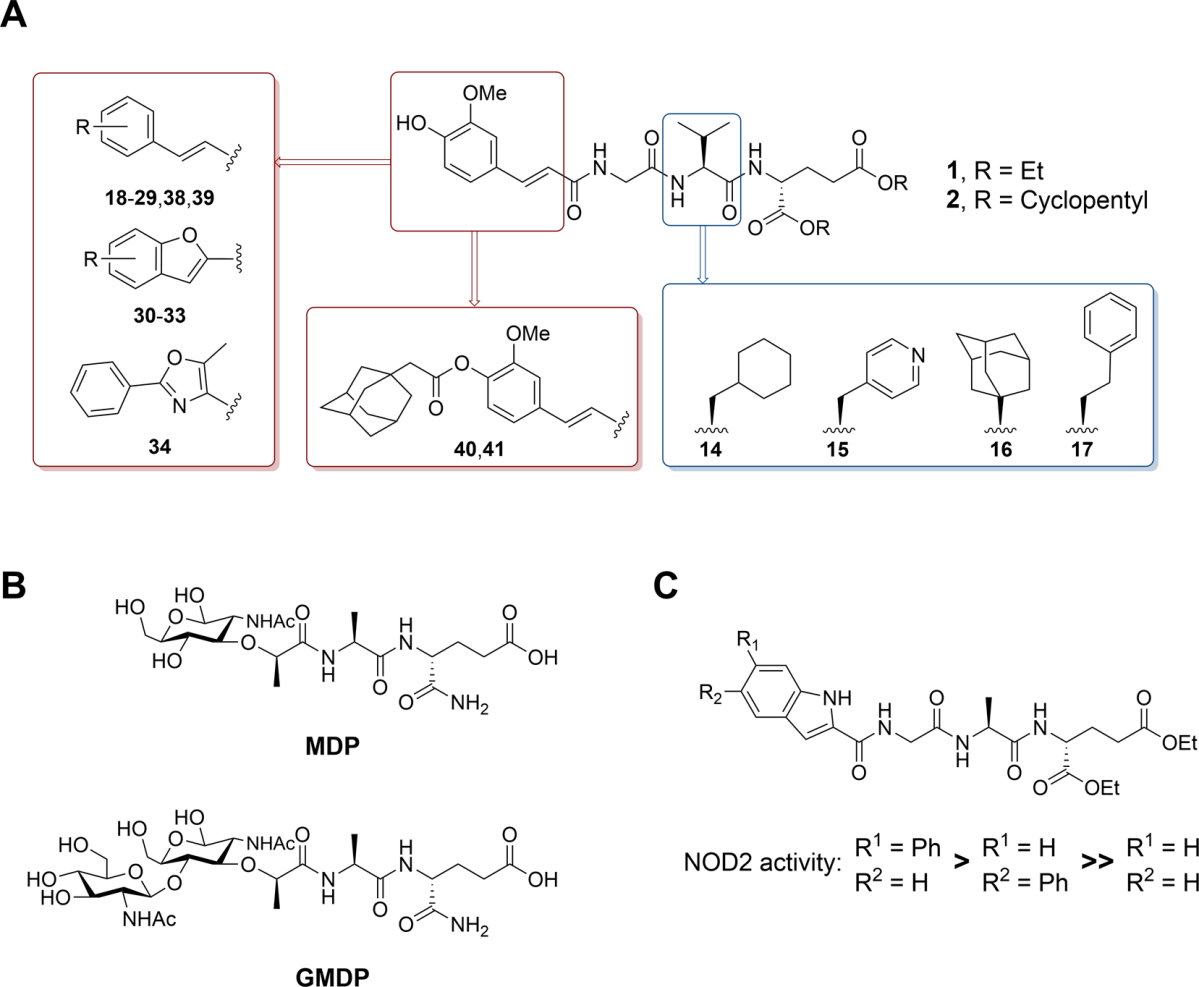
(A) Design of novel desmuramylpeptides based on the structures of **1** and **2**. (B) Structures of muropeptides MDP and GMDP. (C) SAR of indole-based desmuramylpeptides.^24^

Second, the immunostimulatory activity of glucosaminylmuramyl dipeptide (GMDP; Figure 1B) is stronger than that of MDP.^21−23^ The positioning of the additional N-acetylglucosamine moiety in GMDP indicates that there is additional space in the NOD2 ligand binding pocket that is not fully utilized by MDP alone. Furthermore, in our previous exploration of the chemical space of N-acetylmuramic acid mimetics, both 5- and 6-phenylindole derivatives showed stronger NOD2 stimulatory activity compared with the derivative featuring unsubstituted indole (Figure 1C).^24^ This prompted us to evaluate a series of desmuramylpeptides incorporating 3- or 4-substituted cinnamic acid or its conformationally constrained derivatives in an attempt to take advantage of additional potential interactions within the binding pocket. Finally, in addition to incorporating adamantane in the form of (*S*)-adamantylglycine, we also installed it as a cleavable group on the aromatic ring of both **1** and **2**. The adamantane moiety is commonly used in drug design as a hydrophobic auxiliary group and has been used previously to increase the lipophilicity and adjuvant activity of NOD2 agonists.^25,26^

The synthetic strategy involving sequential steps of *tert*-butoxycarbonyl (Boc) protecting group removal and amide bond formation is shown in Scheme 1 and begins with the esterification of d-glutamic acid with thionyl chloride in ethanol to produce the diethyl ester **3**. The linkage of **3** with Boc-protected amino acids (l-cyclohexlyalanine, l-pyridylalanine, (*S*)-adamantylglycine, l-homophenylalanine, and l-valine) using the 1-ethyl-3-(3-(dimethylamino)propyl)carbodiimide (EDC)/1-hydroxybenzotriazole (HOBt)/4-dimethylaminopyridine (DMAP) coupling method gave the corresponding dipeptides **4**–**8**. Acidolytic cleavage of the Boc protecting group with trifluoroacetic acid (TFA) and subsequent coupling to Boc-glycine yielded tripeptides **9**–**13**, which were then subjected to a final round of TFA-mediated Boc deprotection and coupling with either *trans*-ferulic acid or various derivatives/mimetics of cinnamic acid to produce the final acyltripeptides **1** and **14**–**34**.

**Scheme 1:**
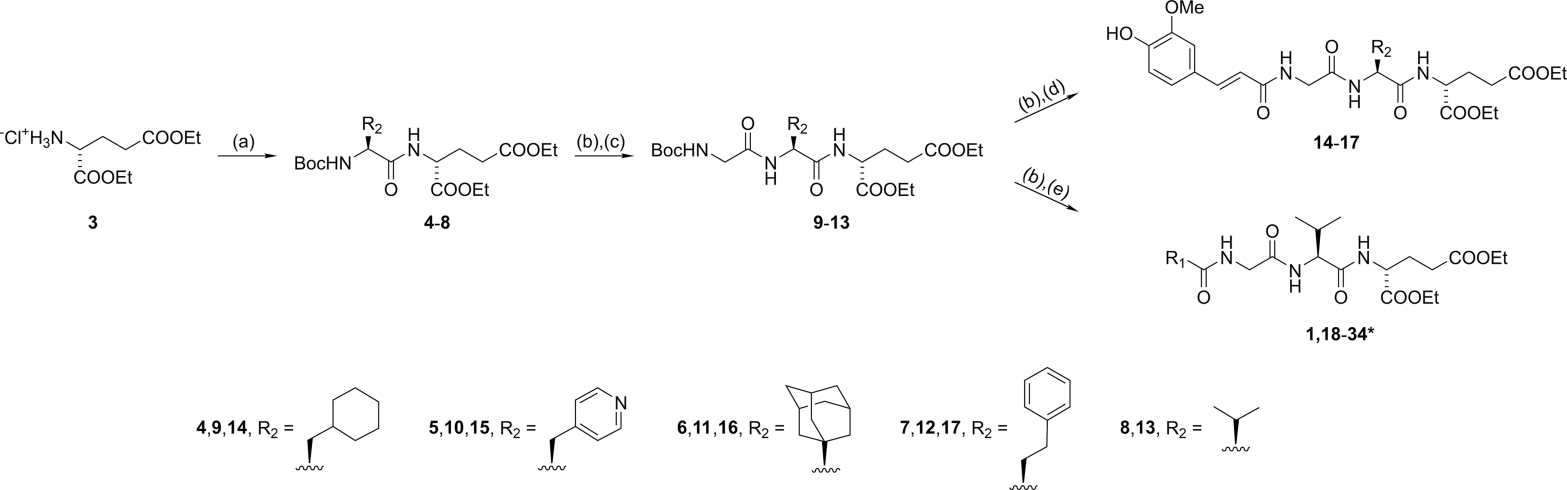
Synthesis of Desmuramylpeptides **1** and **14**–**34** Reagents and conditions: (a) Boc-protected amino acids, EDC, HOBt, DIPEA, DMAP, DMF, rt; (b) TFA/DCM (1:5), rt; (c) Boc-Gly, EDC, HOBt, DIPEA, DMAP, DMF, rt; (d) *trans*-ferulic acid, EDC, HOBt, DIPEA, DMAP, DMF, rt; (e) R_1_COOH, EDC, HOBt, DIPEA, DMAP, DMF, rt. *See Table 1 for definitions of the R_1_ group.

Similarly, desmuramylpeptides featuring a dicyclopentyl-d-glutamic acid moiety were synthesized from Boc-d-glutamic acid, which was esterified with cyclopentanol and EDC to produce the diester **35** (Scheme 2). Two rounds of TFA-mediated Boc deprotection and coupling, first to Boc-l-valine and then to Boc-glycine, gave the corresponding dipeptide **36** and tripeptide **37**, respectively. Finally, **37** was deprotected and coupled to *trans*-ferulic acid, 3-phenylcinnamic acid, or 3-phenoxycinnamic acid to give **2**, **38**, and **39**, respectively.

**Scheme 2:**
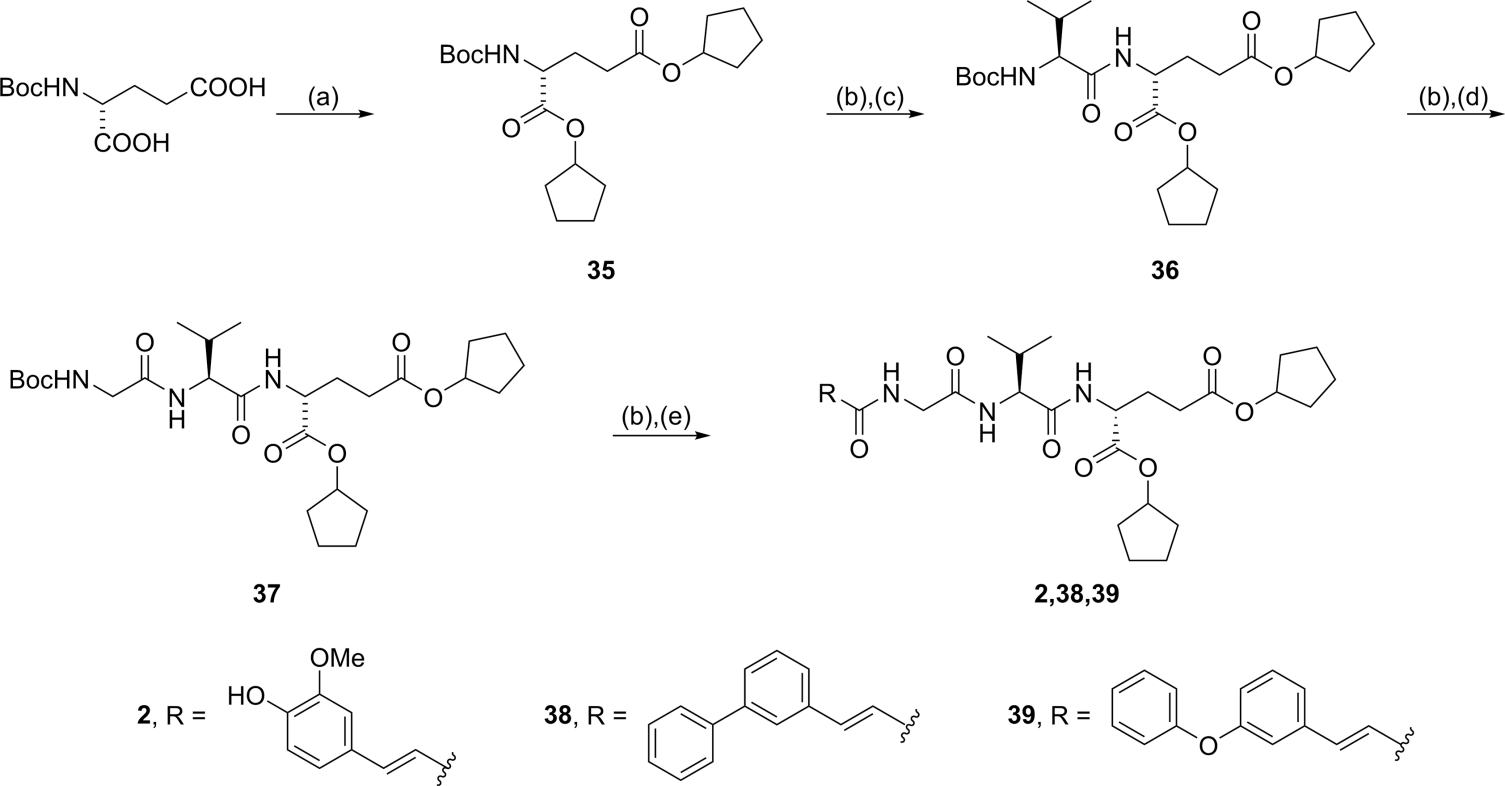
Synthesis of Dicyclopentyl Desmuramylpeptide Derivatives **2**, **38**, and **39** Reagents and conditions: (a) cyclopentanol, EDC, DMAP, DCM, rt; (b) TFA/DCM (1:5), rt; (c) Boc-l-Val, EDC, HOBt, DIPEA, DMAP, DMF, rt; (d) Boc-Gly, EDC, HOBt, DIPEA, DMAP, DMF, rt; (e) RCOOH, EDC, HOBt, DIPEA, DMAP, DMF, rt.

Lastly, the phenol group on the *trans*-ferulic acid moiety of the two lead compounds (**1** and **2**) was decorated with an adamantyl group through EDC/HOBt-mediated esterification with 1-adamantaneacetic acid, producing **40** and **41** (Scheme 3).

**Scheme 3:**
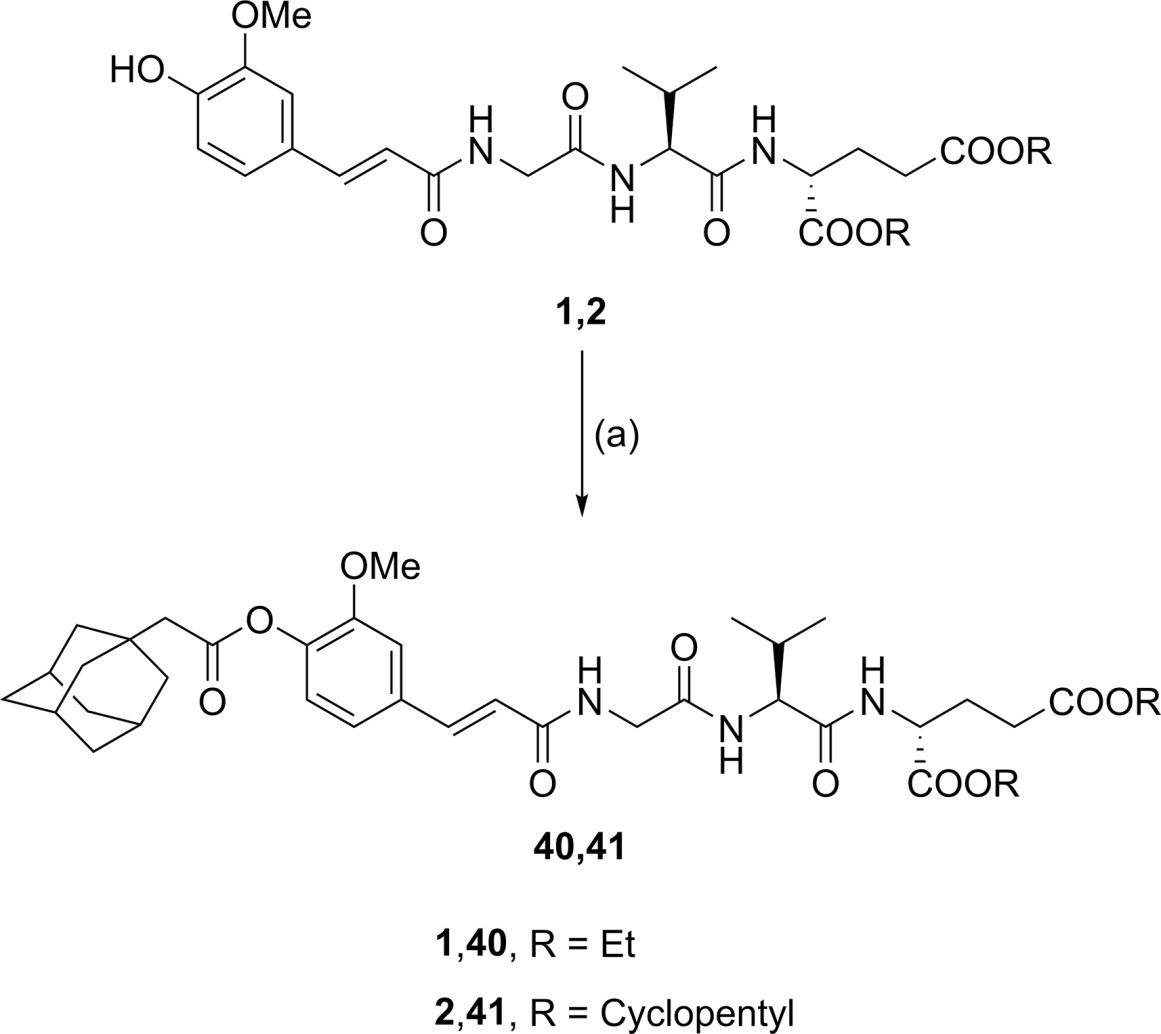
Synthesis of Desmuramylpeptides Decorated with an Adamantyl Group Reagents and conditions: (a) 1-adamantaneacetic acid, EDC, HOBt, DIPEA, DMAP, DMF, rt.

The synthesized desmuramylpeptides were evaluated for their dose-dependent NOD2 activity using the commercially available HEK-Blue NOD2 reporter cell line. This cell line is derived from HEK293 cells by cotransfection of human NOD2 and an NF-κB-inducible secreted embryonic alkaline phosphatase (SEAP). Activation of NOD2 and subsequently of NF-κB leads to the expression and secretion of SEAP, the level of which can be quantified colorimetrically. Of note, none of the compounds tested exhibited any cytotoxicity against HEK-Blue NOD2 cells, as determined by the (3-(4,5-dimethylthiazol-2-yl)-5-(3-carboxymethoxyphenyl)-2-(4-sulfo-phenyl)-2H-tetrazolium) (MTS) method (Figure S1; see the SI).

We first investigated whether the NOD2 activity of desmuramylpeptides can be improved by modifications of the amino acid structure. The results summarized in Table 1 show that increasing the size of the amino acid side chain beyond the isopropyl fragment of l-valine present in both lead compounds (**1** [EC_50_ = 89 nM], **2** [EC_50_ = 45 nM]) resulted in a significant decrease in NOD2 agonistic activity. The low micromolar activity of the l-cyclohexylalanine derivative (**14**; EC_50_ = 1.09 μM) and the inactivity of the (*S*)-adamantylglycine derivative (**16**; EC_50_ > 10 μM) suggest that the lipophilic side pocket may not be deep enough to accommodate bulkier side chains. This is in part contrasted by the nanomolar activity of the l-homophenylalanine derivative (**17**; EC_50_ = 420 nM). However, the additional methylene group likely allows for greater flexibility and thus better positioning of the bulky aromatic ring. Furthermore, despite our previous success with desmuramylpeptides incorporating l-phenylalanine, the l-pyridylalanine derivative (**15**; EC_50_ > 10 μM) exhibited only minor NOD2 agonistic activity at the highest concentration tested (20 μM), confirming our observations that the pocket is only accessible to strictly hydrophobic groups.

**Table 1:**
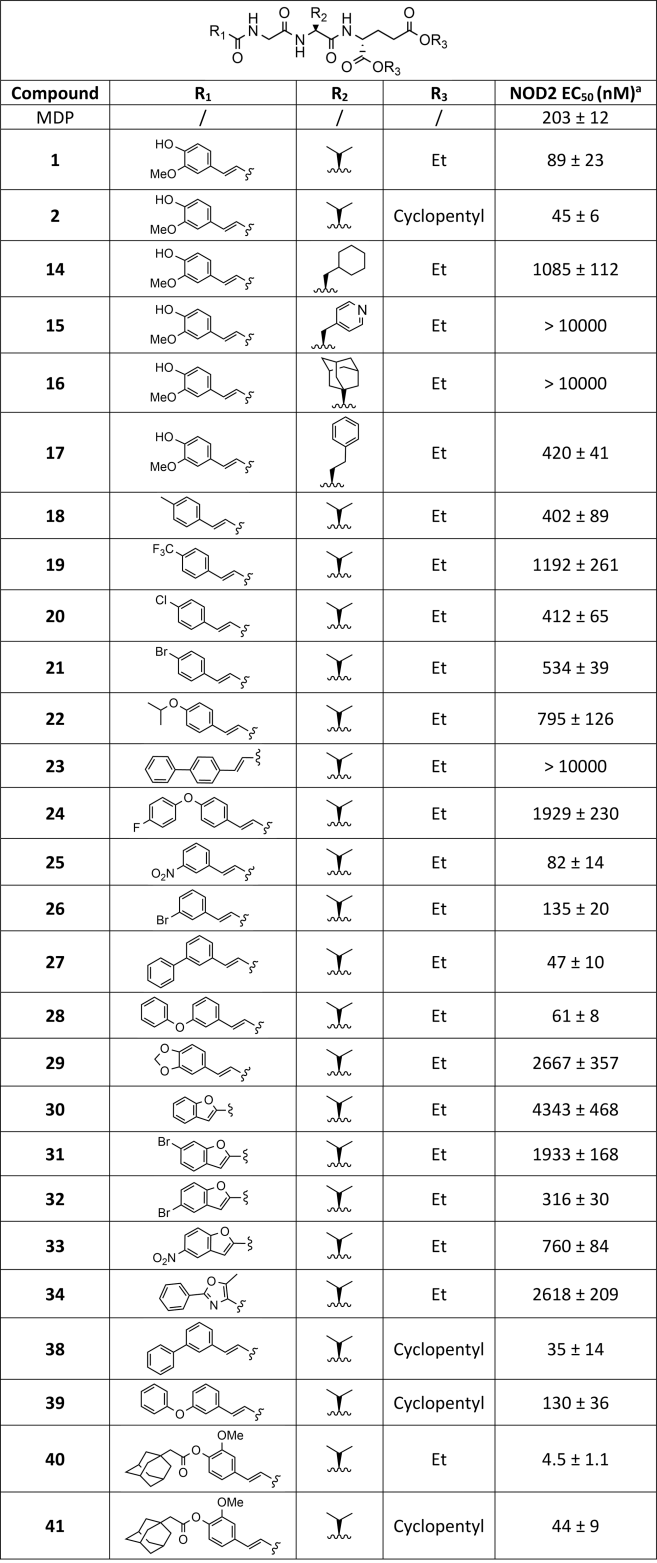
NOD2 Agonistic Activities of Novel Desmuramylpeptides

a EC_50_ values are means ± SEMs of at least three independent experiments with 8 or 16 concentrations used (from 0.1 nM to 20 μM).

We then conducted a focused SAR exploration around the cinnamoyl moiety. Desmuramylpeptides incorporating 4-substituted cinnamic acid showed lower activity compared to the 3-methoxy-4-hydroxy substitution pattern found in both lead compounds. The potency of these compounds appeared to be related to the steric bulk of the substituent. Derivatives with smaller functional groups (Me, **18** [EC_50_ = 402 nM]; Cl, **20** [EC_50_ = 412 nM]; Br, **21** [EC_50_ = 534 nM]) exhibited the strongest activity, followed by derivatives incorporating an isopropoxy (**22**; EC_50_ = 795 nM) or a CF_3_ group (**19**; EC_50_ = 1.19 μM). A further increase in size to a phenyl group resulted in the inactive derivative **23** (EC_50_ > 10 μM). Interestingly, increasing the flexibility of the phenyl group via an ether bond, resulting in the closely related 4-fluorophenoxy (**24**; EC_50_ = 1.93 μM) derivative, moderately improved the activity.

In contrast to the 4-substituted derivatives, the introduction of a group at the 3-position of the aromatic ring was considerably better tolerated, with the phenyl (**27**; EC_50_ = 47 nM) and phenoxy (**28**; EC_50_ = 61 nM) derivatives exhibiting approximately the same potency as lead compound **2**, closely followed by the nitro (**25**; EC_50_ = 82 nM) and bromo (**26**; EC_50_ = 135 nM) derivatives. Finally, the bridge formation between the 4-hydroxy and 3-methoxy groups in **1**, which gave the 3,4-methylenedioxy derivative **29** (EC_50_ = 2.67 μM), surprisingly resulted in a 30-fold reduction in potency, indicating that the 4-hydroxy group of **1** likely contributes to binding as an H-bond donor.

Conformational constraint of the cinnamoyl moiety using benzofuran (**30**; EC_50_ = 4.34 μM) and 5-methyl-2-phenyloxazole (**34**; EC_50_ = 2.62 μM) proved to be detrimental to NOD2 activation. Decorating the benzofuran with bromine and nitro groups moderately improved this activity with a similar position-dependent trend as for the cinnamic acid derivatives; namely, the introduction of bromine (**32**; EC_50_ = 316 nM) or nitro (**33**; EC_50_ = 760 nM) to the 5-position (corresponding to the 3-position of cinnamic acid) yielded more pronounced NOD2 activity than the introduction of bromine to the 6-position (**31**; EC_50_ = 1.93 μM) (corresponding to the 4-position of cinnamic acid).

In the last group of modifications, we increased the lipophilicity of the most potent 3-phenylcinnamoyl (**27**) and 3-phenoxycinnamoyl (**28**) derivatives by replacing the diethyl-d-glutamic acid moiety with dicyclopentyl-d-glutamic acid in an attempt to reproduce the beneficial effects of this transformation as seen in **1** and **2**. Conversion of **27** (EC_50_ = 47 nM) to its more lipophilic congener **38** (EC_50_ = 35 nM) did not significantly improve NOD2 activation, whereas the conversion of **28** (EC_50_ = 61 nM) to **39** (EC_50_ = 130 nM) resulted in a slight decrease in activity, by a factor of 2. Conversely, increasing the lipophilicity of **1** by attaching an adamantane fragment to the aromatic ring via a cleavable ester bond improved the activity by a factor of 20. The resulting prodrug derivative **40** activated NOD2 with an EC_50_ value of 4.5 nM, making it the first single-digit nanomolar and the most potent NOD2 agonist of its structural type to date. On the other hand, when applied to **2**, the same transformation resulted in the equipotent derivative **41** (EC_50_ = 44 nM).

The specificities of all compounds were determined by pretreating HEK-Blue NOD2 cells with a known NOD2 antagonist (Figure S2A; see the SI),^27^ before the addition of desmuramylpeptides. The comparative reduction in measured activity in response to pretreatment with the NOD2 antagonist confirmed that the NF-κB transcriptional activity after stimulation with all compounds was reliant on the activation of NOD2 (Figure S2B; see the SI). Furthermore, the selectivity of all synthesized desmuramylpeptides against NOD1 was determined in an analogous assay using HEK-Blue NOD1 cells. None of the tested compounds induced any significant NOD1 activation at 2 μM, confirming selectivity for NOD2 (Figure S3; see the SI).

Given that cells of the monocyte-macrophage lineage are among the primary responders to NOD2 stimuli,^28^ we also examined the effect of **40** on the NF-κB transcriptional response in RAW-Blue reporter cells. These cells, which, similarly to the HEK-Blue cells described above, stably express an NF-κB-inducible SEAP reporter gene, are derived from RAW264.7 mouse macrophages, which in addition to NOD2 also express NOD1 and most Toll-like receptors (TLRs). Consistent with the results obtained in HEK-Blue NOD2 cells, **40** was found to activate RAW-Blue cells in a NOD2-dependent manner, as pretreatment with a NOD2 antagonist significantly reduced the observed NF-κB transcriptional response (Figure S4A; see the SI).

Encouraged by the potent NOD2 activity of **40**, we preliminarily evaluated its immunostimulatory potential in human primary peripheral blood mononuclear cells (PBMCs). Specifically, we first investigated the ability of **40** to induce cytokine production in PBMCs. While activation of NOD2 alone is sufficient to elicit cytokine responses, these responses are usually of low intensity. However, NOD2 agonists can act in synergy with ligands of other innate immune receptors and proinflammatory cytokines to induce considerably stronger responses. We were particularly intrigued by the synergistic interactions between NOD2, TLR4, and IFN-γ. The synergy between NOD2 agonists and TLR4 agonists in terms of cytokine secretion, including IFN-γ, is well-established.^29,30^ Similarly, IFN-γ has previously been reported to augment the MDP-mediated activation and cytokine production by dendritic cells and macrophages.^31,32^

As shown in Figure 2, MDP, **2**, and **40** induced modest production of IL-1β, IL-6, IL-8, and TNF-α. Similar to the HEK-Blue NOD2 cellular assays, the effects of **40** were entirely NOD2-dependent, as pretreatment with an NOD2 antagonist restored cytokine production to the levels of untreated control cells (Figure S5; see the SI). While IFN-γ alone induced negligible cytokine secretion, it synergistically increased the **40-**induced production of IL-1β, IL-6, and TNF-α but had no apparent effect on the production of IL-8. Conversely, stimulation with LPS expectedly resulted in a strong increase in cytokine production. This effect was further enhanced by **40** in a superadditive manner, i.e., the levels of IL-1β, IL-6, and TNF-α after simultaneous stimulation with both **40** and LPS were higher than the sum of the responses after stimulation with the individual immunostimulants.

**Figure 2:**
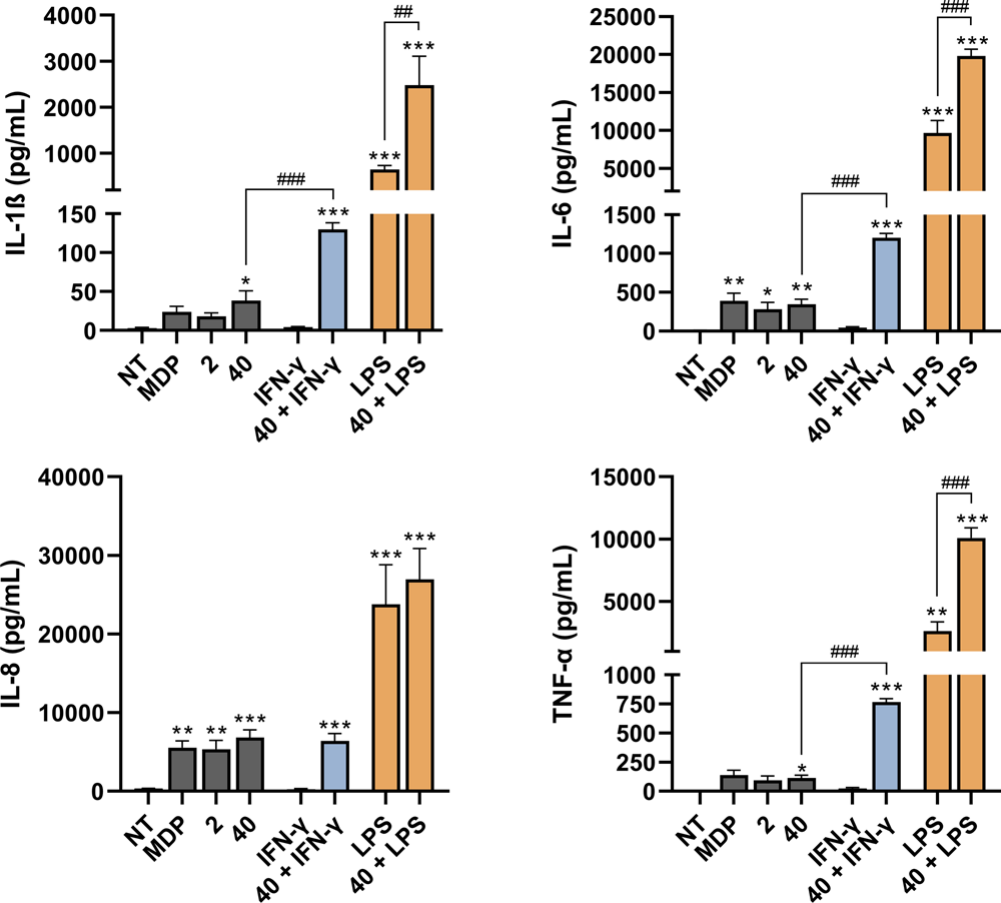
Synergistic effects of **40** with LPS and IFN-γ on induction of cytokine production from PBMCs. Cytokine concentrations were measured after 18 h of stimulation with NOD2 agonists (1 μM) in the presence or absence of LPS (10 ng/mL) and IFN-γ (200 U/mL) or the corresponding vehicle (0.1% DMSO; NT). Data are expressed as mean ± SEM of three independent experiments. Statistical significance was determined by one-way ANOVA followed by Bonferroni’s multiple-comparisons test; *, *p* < 0.05, **, *p* < 0.01, ***, *p* < 0.001 versus vehicle-treated control; *##*, *p* < 0.01, *###*, *p* < 0.001 versus **40** or LPS alone.

In addition, we also investigated the ability of **40**, both alone and in combination with LPS and IFN-γ, to induce direct and nonspecific cytolytic activity of PBMCs against cancer cells. For this purpose, we used a functional cytotoxicity assay based on the coincubation of preactivated PBMCs and fluorescently labeled K562 cancer cells.^33^ Among the heterogeneous subpopulations of PBMCs, natural killer (NK) cells and, to a lesser extent, monocytes represent the main effector cell types in this assay. NK cells play an essential role in tumor and viral immunosurveillance, both through direct cytolytic destruction of aberrant cells and by facilitating the recruitment and activation of other immune cells through the secretion of cytokines.^34^ In addition to their well-established role in cancer immunotherapy, NK cells thus also provide an attractive target in the development of vaccine adjuvants, particularly when Th1 cellular immune responses are preferred.^35^ Other cell populations, however, may contribute to the magnitude of NK cell-mediated responses via cytokine secretion. To account for the contribution of these accessory cells and to more accurately represent *in vivo* conditions, the entire PBMC population was used instead of isolated NK cells.

As illustrated by Figure 3, stimulation of PBMCs with **40** resulted in a 1.28-fold, albeit insignificant, increase in the ratio of dead K562 cells, whereas **2** showed no activity. This is consistent with our previous observations where, despite the low nanomolar NOD2 activity, both **1** and **2** failed to induce the cytotoxic activity of PBMCs, whereas the introduction of lipophilic groups, in particular a C_18_ stearoyl tail, to the aromatic ring significantly enhanced the observed response.^18^

**Figure 3:**
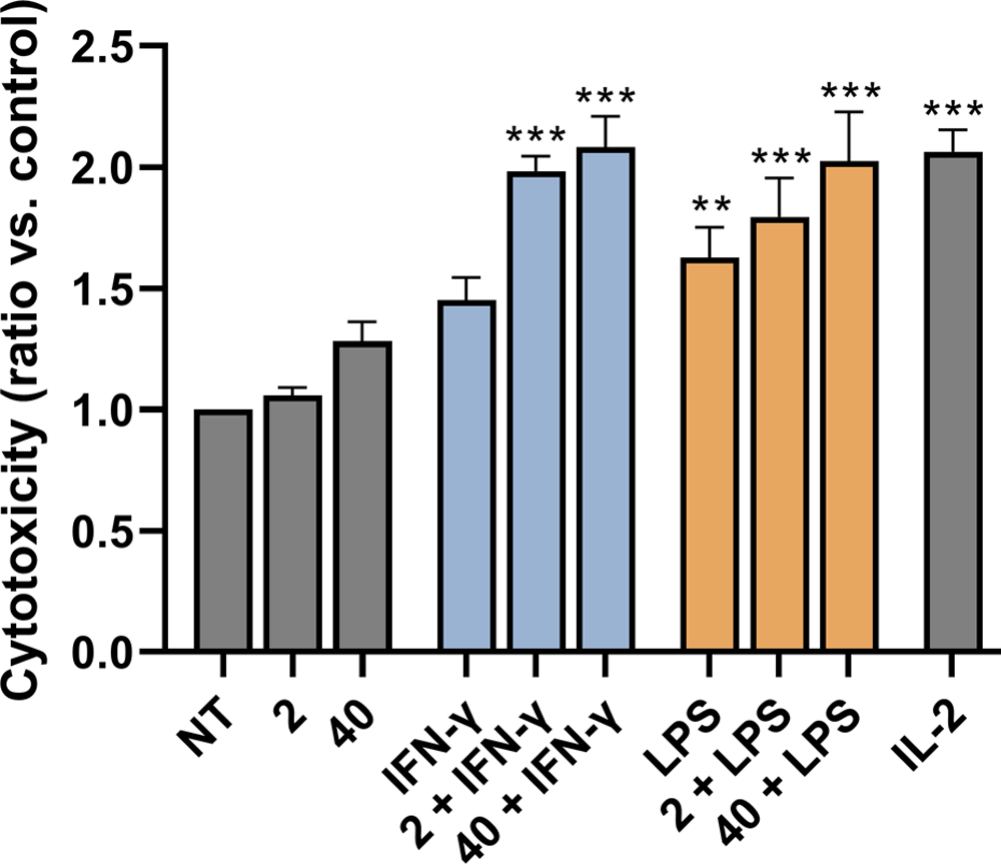
**2** and **40** synergize with LPS and IFN-γ to induce the cytotoxicity of PBMCs against K562 cancer cells. Following 18 h of stimulation of PBMCs with **2** (1 μM) or **40** (1 μM) in the presence or absence of LPS (1 μg/mL) and IFN-γ (200 U/mL) or the corresponding vehicle (0.1% DMSO; NT), K562 cells were added in a 40:1 effector to target cell ratio. Cytotoxicity was determined after 4 h of coincubation. IL-2 (200 U/mL) was used as the positive control. Data are shown as relative activities to vehicle-treated control (0.1% DMSO) and are the means ± SEMs of duplicates of two independent experiments. Statistical significance was determined by one-way ANOVA followed by Dunnet’s multiple-comparisons test; **, *p* < 0.01, ***, *p* < 0.001.

While NK cells express functional NOD2 and respond to stimulation by MDP, this response is suboptimal and requires the contribution of other innate immune receptors, such as TLR4, and accessory cell-derived cytokines, such as IL-12 and IFN-α, to induce optimal functional responses.^34,36^ The combination of a NOD2 agonist and IFN-γ was reported to be directly toxic to acute myeloid leukemia (AML) cells through the induction of apoptosis while also enhancing the maturation and cytokine production of NK cells *in vivo*, leading to a significant reduction of the AML disease burden.^37^ Similarly, activation of NOD2 has been reported to enhance TLR4-mediated NK cell activation *in vivo*.^38^

Costimulation with **2** and **40** significantly enhanced the LPS- and IFN-γ-induced cytotoxic activity of PBMCs, with all combinations showing comparable activity to the positive control IL-2 (Figure 3). Consistent with the results obtained in the absence of costimuli, **40** exhibited slightly stronger activity compared to that of **2**. In contrast to previous reports,^37^ however, the cytotoxicity was entirely PBMC-dependent, i.e., cotreatment with **40** and IFN-γ did not induce apoptosis in K562 cells in the absence of PBMCs (data not shown).

In conclusion, the results presented here shed additional light on the SAR of desmuramylpeptide NOD2 agonists. Accordingly, compound **40** was identified as the first desmuramylpeptide with NOD2 stimulating activity in the single-digit nanomolar range and the most potent NOD2 agonist in its structural class to date. Furthermore, our study highlights the capacity of small molecule NOD2 agonists to synergize with TLR4 agonists and IFN-γ in terms of inducing cytokine production by PBMCs and stimulating their nonspecific cytolytic activity against K562 cancer cells. Although our knowledge of such immune synergies is still limited, they offer opportunities for significant improvement of cancer immunotherapeutic approaches. Moreover, such synergies can also be exploited in the modulation of adaptive immunity and consequently in the development of improved vaccine adjuvants.^39^ The results presented here are noteworthy and deserve further investigation. Therefore, the clinical utility of **40** and other desmuramylpeptides, both alone and in combination with other immunostimulants, will be further evaluated in future studies of *in vivo* adjuvant activity.

## Supporting Information Available

Supporting Figures; Supporting Tables; full experimental details and compound characterization of all compounds as well as experimental conditions of biological assays (PDF)

## Abbreviations

DCM: dichloromethane
DIPEA: *N*,*N*-diisopropylethylamine
DMAP: 4-dimethylaminopyridine
EDC: 1-ethyl-3-(3-(dimethylamino)propyl)carbodiimide
CFSE: carboxyfluorescein succinimidyl ester
GMDP: glucosaminylmuramyl dipeptide
HOBt: 1-hydroxybenzotriazole
IFN: interferon
MDP: muramyl dipeptide
NF-κB: nuclear factor κB
NK: natural killer
NOD: nucleotide-binding oligomerization domain
PBMC: peripheral blood mononuclear cell
SEAP: secreted embryonic alkaline phosphatase
TFA: trifluoroacetic acid
TLR: Toll-like receptor
