# Supplementary material for: Discovery of Desmuramylpeptide NOD2 Agonists with Single-Digit Nanomolar Potency
Source: ACS Med Chem Lett. 2022 Jul 18;13(8):1270–7. doi: 10.1021/acsmedchemlett.2c00121 (PMC9377006; doi:10.1021/acsmedchemlett.2c00121)
Supplement: Supplementary file 1 — ml2c00121_si_001.pdf [file ml2c00121_si_001.pdf]

SUPPORTING INFORMATION

Discovery of desmuramylpeptide NOD2 agonists with single-digit nanomolar potency

Samo Guzelj, Špela Bizjak, Žiga Jakopin*

Faculty of Pharmacy, University of Ljubljana, SI-1000 Ljubljana, Slovenia

*Corresponding author:

Žiga Jakopin

Phone: +386 1 4769 646

Fax: + 386 1 4258 031

E-mail address: ziga.jakopin@ffa.uni-lj.si

Table of Contents

1 Supporting figures 2

2 Supporting tables........ 7

3 Molecular docking .... **Error! Bookmark not defined.**

4 Experimental procedures........ 8

5 Biology 18

6 References........ 47

1 Supporting figures

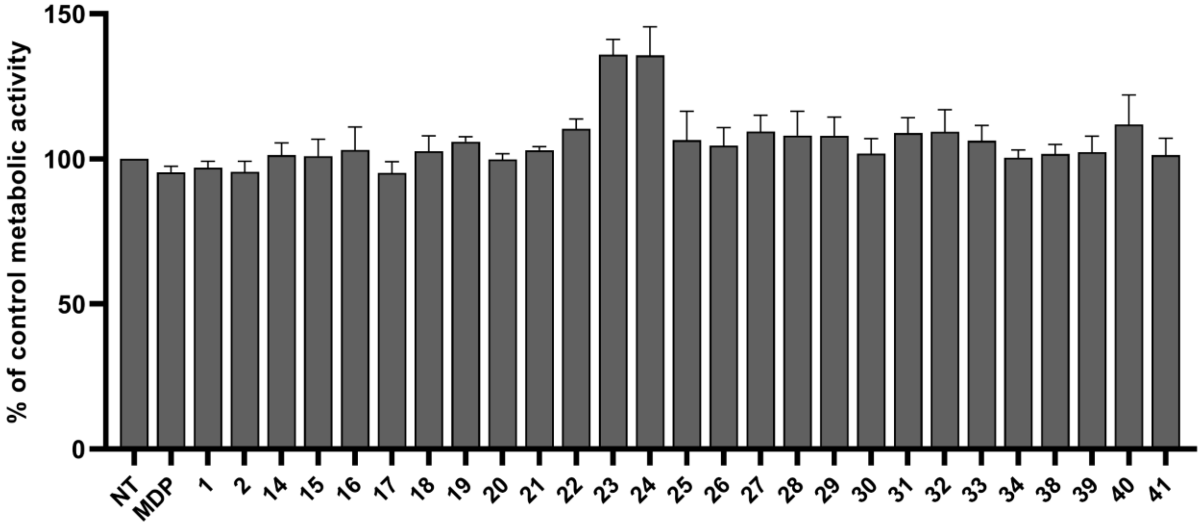

Figure S1. Metabolic activities of HEK-Blue NOD2 cells after 18 h stimulation with MDP (2 µM) or the desmuramylpeptides (2 µM). The results are shown relative to that of the untreated control (0.1% DMSO; NT). Data are means ±SEM of two independent experiments.

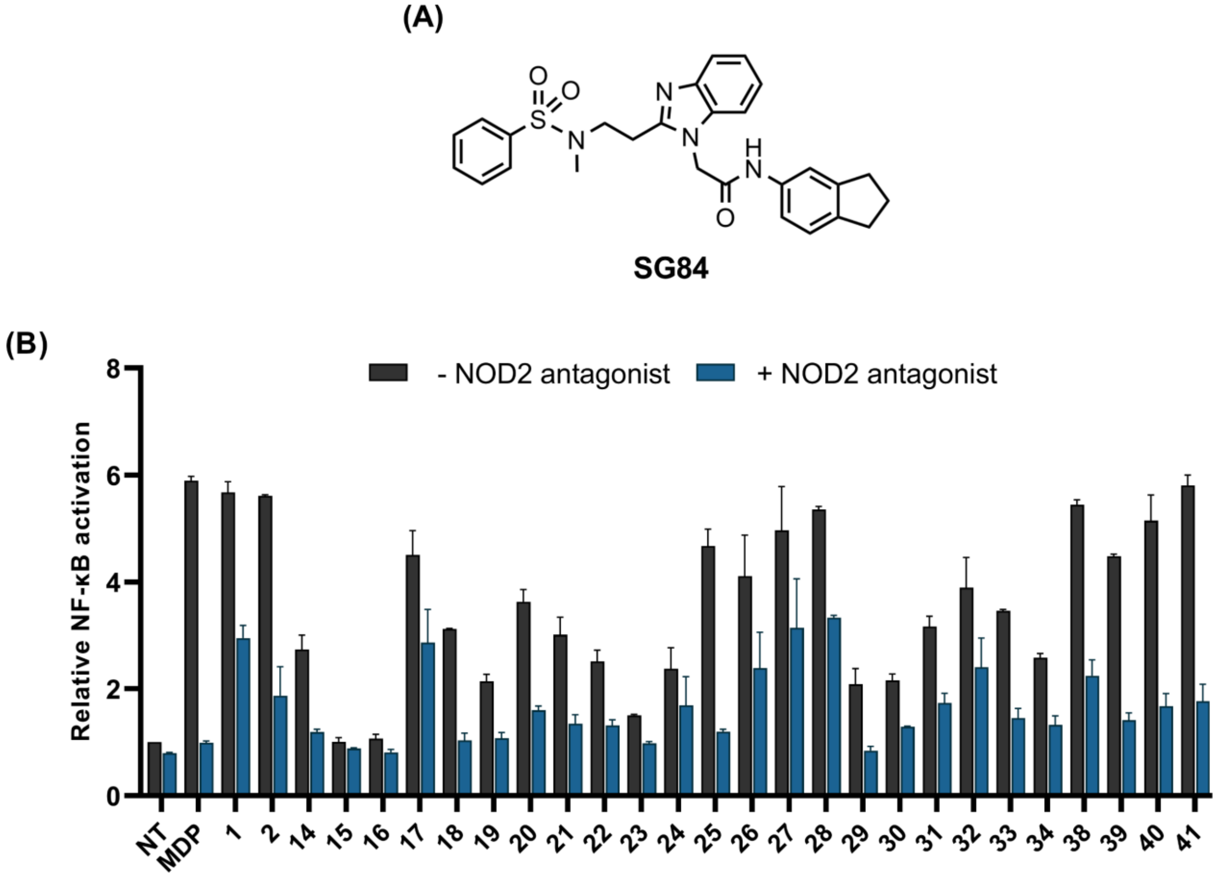

Figure S2. (A) Chemical structure of the NOD2 antagonist **SG84**. (B) Effects of NOD2 antagonist pre-treatment on MDP- and desmuramylpeptide-induced NF-kB transcriptional activities. HEK-Blue NOD2 cells were pre-incubated for 1 h in the presence or absence of **SG84** (10 µM), before the addition of MDP (2 µM) or desmuramylpeptides (2 µM). The SEAP activities after 18 h of incubation are shown relative to that of the untreated control (0.1% DMSO; NT). Data are means ± SEM of two independent experiments.

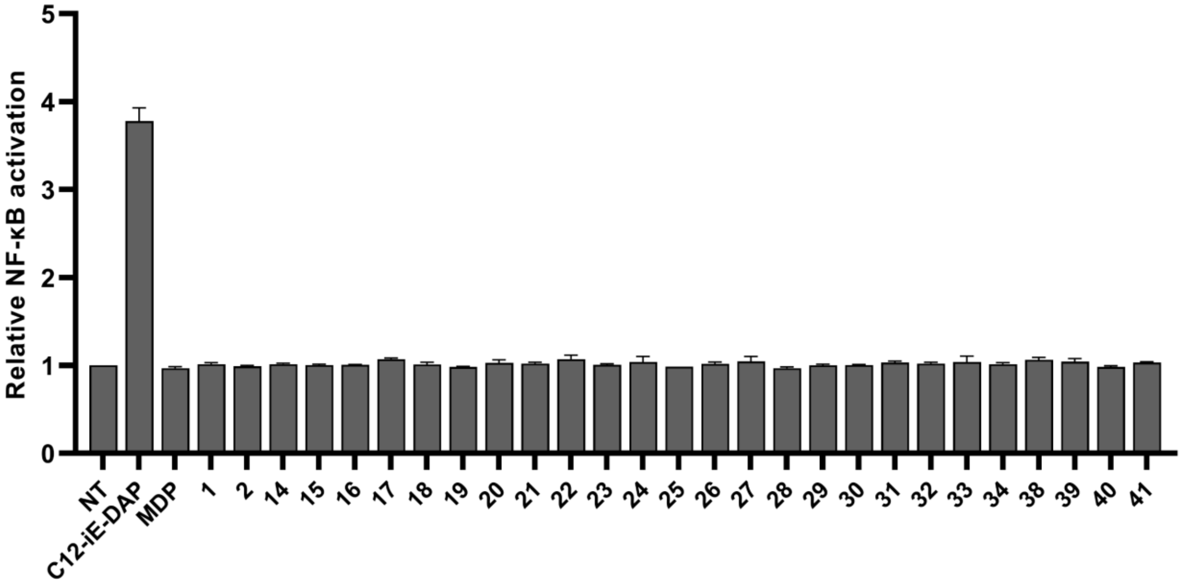

Figure S3. NOD1 agonistic activities of desmuramylpeptides. HEK-Blue NOD1 cells were treated with C12-iE-DAP (100 nM) or desmuramylpeptides (2 µM) for 18 h. The SEAP activities are shown relative to that of the untreated control (0.1% DMSO; NT). Data are means ± SEM of two independent experiments. C12-iE-DAP was used as the positive control.

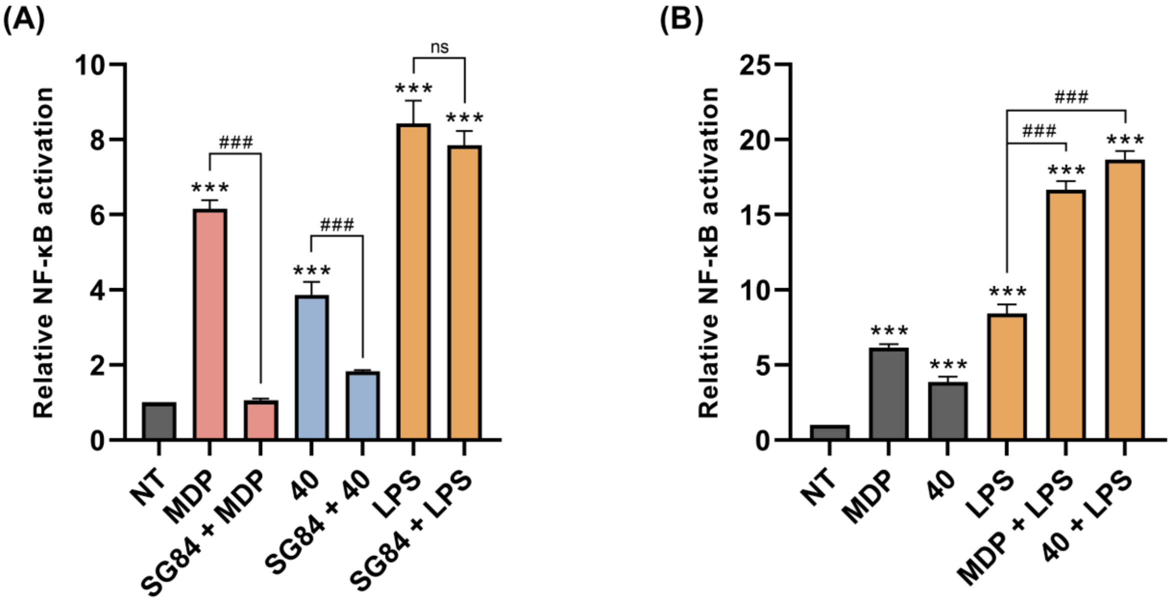

Figure S4. NF-kB transcriptional response in the RAW-Blue mouse macrophage reporter cell line. (A) RAW-Blue cells were pre-incubated for 1 h in the presence or absence of the NOD2 antagonist **SG84** (10 µM), before the addition of MDP (1 µM), **40** (1 µM), or LPS (10 ng/mL). (B) RAW-Blue cells were treated with MDP (1 µM) or **40** (1 µM), in the presence or absence of LPS (10 ng/mL). The SEAP activities after 18 h of incubation are shown relative to that of the untreated control (0.1% DMSO; NT). Data are means ± SEM of four independent experiments. Statistical significance was determined by one-way ANOVA followed by Bonferroni's multiple comparisons test; ***, $p < 0.001$ *versus* vehicle-treated control; *###*, $p < 0.001$ *versus* MDP, **40**, or LPS alone; ns, not significant.

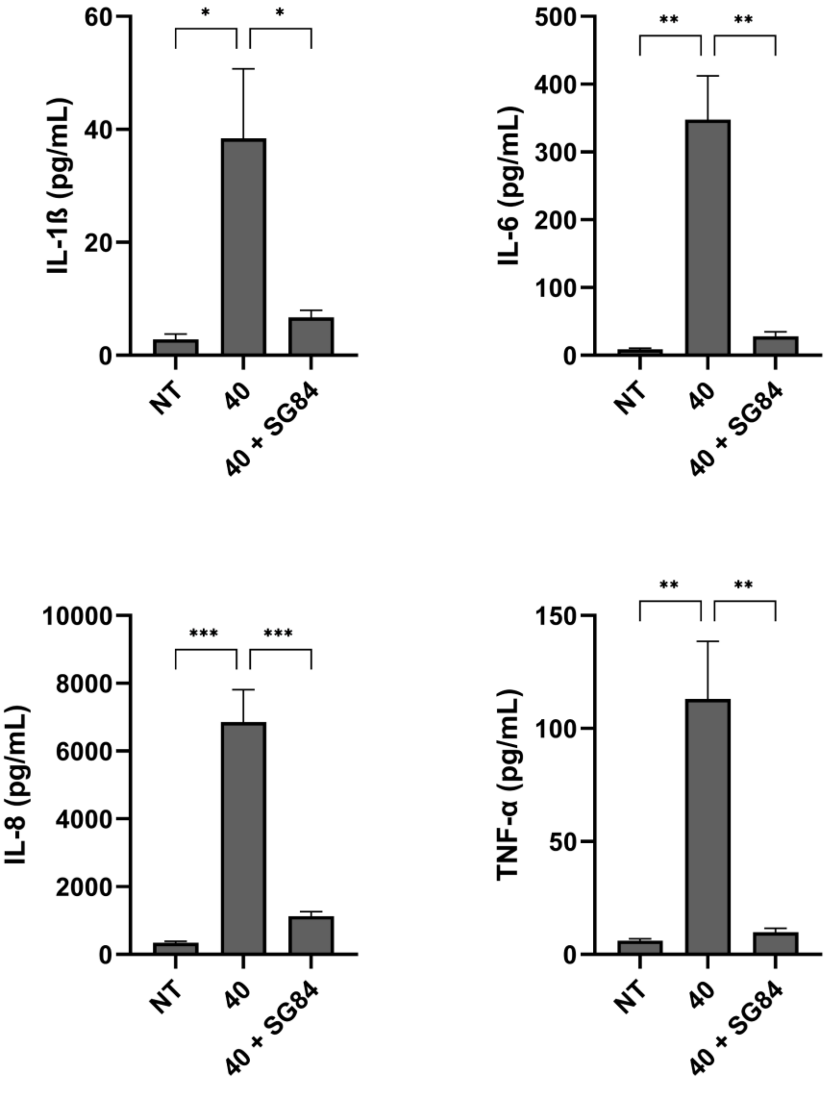

Figure S5. The effect of **40** on the production of cytokines from human PBMCs is NOD2-dependent. PBMCs were pre-incubated for 1 h in the presence or absence of the NOD2 antagonist **SG84** (10 µM), before the addition of **40** (1 µM). Cytokine concentrations in supernatants were measured after 18 h stimulation. Data are expressed as means ± SEM of 3 independent experiments. Statistical significance was determined by one-way ANOVA followed by Bonferroni's multiple comparisons test; **, $p < 0.01$, ***, $p < 0.001$.

2 Supporting tables

Table S1. Calculated logP values using Schrödinger QikProp software.

Compound	ClogP
**1**	3.101
**2**	4.331
**14**	4.306
**15**	3.118
**16**	4.068
**17**	3.830
**18**	3.917
**19**	4.611
**20**	4.097
**21**	4.173
**22**	4.541
**23**	5.294
**24**	5.585
**25**	2.518
**26**	3.811
**27**	4.588
**28**	5.134
**29**	3.055
**30**	3.229
**31**	3.799
**32**	3.796
**33**	2.404
**34**	3.043
**38**	6.150
**39**	6.856
**40**	5.910
**41**	7.061

3 Experimental procedures

3.1 Safety statement

No unexpected or unusually high safety hazards were encountered.

3.2 Materials

Chemicals were obtained from Sigma-Aldrich (St. Louis, MO, USA), Tokyo Chemical Industry (Tokyo, Japan), Acros Organics (Geel, Belgium), Enamine (Monmouth Junction, NJ, USA), and Apollo (Stockport, UK), and were used without further purification. Analytical TLC was performed on Merck 60 F254 silica gel plates (0.25 mm), with visualization using ultraviolet light, ninhydrin, and potassium permanganate. Flash column chromatography was carried out on Merck silica gel 60 (particle size 240−400 mesh). ^{1}H and ^{13}C NMR spectra were recorded at 400 MHz and 101 MHz, respectively, on an Avance III spectrometer (Bruker Corporation, Billerica, MA, USA) in $CDCl_3$ or DMSO-d_6 with tetramethylsilane as the internal standard. Mass spectra were obtained using an Exactive Plus orbitrap mass spectrometer (Thermo Fisher Scientific, Waltham, MA, USA) or on Expression CMS mass spectrometer (Advion Inc., Ithaca, NY, USA). Analytical UHPLC analyses were performed on a Dionex UltiMate 3000 Rapid Separation Binary System (Thermo Fisher Scientific, Waltham, MA, USA) equipped with an autosampler, a binary pump system, a photodiode array detector, a thermostated column compartment, and the Chromeleon Chromatography data system. The column used was Waters Acquity UPLC BEH C18 (1.7 µm, 2.1 × 50 mm), with a flow rate of 0.3 mL/min. The eluent was a mixture of 0.1% trifluoroacetic acid (TFA) in water (A) and acetonitrile (B), with a gradient of (%B): 0–10 min, 5–95%; 10–12 min, 95%; 12–12.5 min, 95-5%. The columns were thermostated at 40 °C. The purity of all biologically tested compounds was >90%.

Compounds **1**, **2**, **3**, **8**, **13**, **35**, **36**, and **37** were prepared as described previously.[1,2] NOD2 antagonist was synthesized as described previously.[3]

3.3 General synthetic procedures

3.3.1 General procedure A: TFA-Mediated Acidolysis

The Boc-protected compound was added to an ice-chilled stirred mixture of TFA and dichloromethane (DCM) (1:5), and the mixture was allowed to warm to room temperature. After 3 h, the solvent was evaporated off *in vacuo*. The residue was washed three times with diethyl ether.

3.3.2 General procedure B: EDC-Mediated Coupling

To an ice-chilled stirred solution of the requisite amine/phenol (1.0 – 1.2 eq) and carboxylic acid (1.0 – 1.2 eq) in dry DMF, DIPEA (3 eq) was added. After stirring for 15 min, 1-hydroxybenzotriazole (HOBt) (1.2 eq), 1-ethyl-3-(3-(dimethylamino)propyl)carbodiimide (EDC) (1.2 eq) and a catalytic amount of 4-dimethylaminopyridine (DMAP) were added, and the mixture was allowed to warm to room temperature. The stirring was continued overnight, after which the mixture was diluted with EtOAc (30 mL) and washed

with 1 M HCl (2× 15 mL), saturated $NaHCO_3$ solution (2× 15 mL) and brine (15 mL). The organic layer was dried over anhydrous Na_2SO_4 and concentrated *in vacuo*.

3.4 Diethyl ((*S*)-2-((tert-butoxycarbonyl)amino)-3-cyclohexylpropanoyl)-D-glutamate (4)

Synthesized from **3** (0.600 g, 2.50 mmol) and (*S*)-2-((tert-butoxycarbonyl)amino)-3-cyclohexylpropanoic acid (0.639 g, 2.35 mmol) using General procedure B. Yellow oil (0.654 g, 61% yield). ^1H NMR (400 MHz, DMSO-d_6) δ 8.20 (d, *J* = 7.9 Hz, 1H), 6.80 (d, *J* = 8.4 Hz, 1H), 4.26 – 4.16 (m, 1H), 4.12 – 3.96 (m, 5H), 2.33 (t, *J* = 7.6 Hz, 2H), 2.06 – 1.92 (m, 1H), 1.90 – 1.74 (m, 1H), 1.72 – 1.51 (m, 5H), 1.45 – 1.29 (m, 11H), 1.25 – 1.02 (m, 10H), 0.93 – 0.80 (m, 2H).

3.5 Diethyl ((*S*)-2-((tert-butoxycarbonyl)amino)-3-(pyridin-4-yl)propanoyl)-D-glutamate (5)

Synthesized from **3** (0.275 g, 1.15 mmol) and (*S*)-2-((tert-butoxycarbonyl)amino)-3-(pyridin-4-yl)propanoic acid (0.336 g, 1.26 mmol) using General procedure B. Colorless oil (0.289 g, 56% yield). ^1H NMR (400 MHz, DMSO-d_6) δ 8.45 (d, *J* = 6.0 Hz, 2H), 8.40 (d, *J* = 7.8 Hz, 1H), 7.27 (d, *J* = 6.0 Hz, 2H), 7.02 (d, *J* = 8.8 Hz, 1H), 4.36 – 4.19 (m, 2H), 4.16 – 3.98 (m, 4H), 3.01 – 2.89 (m, 1H), 2.80 – 2.72 (m, 1H), 2.29 (t, *J* = 7.6 Hz, 2H), 2.04 – 1.89 (m, 1H), 1.91 – 1.75 (m, 1H), 1.29 (s, 9H), 1.24 – 1.12 (m, 6H).

3.6 Diethyl ((*S*)-2-(adamantan-1-yl)-2-((tert-butoxycarbonyl)amino)acetyl)-D-glutamate (6)

Synthesized from **3** (0.145 g, 0.605 mmol) and (*S*)-2-(adamantan-1-yl)-2-((tert-butoxycarbonyl)amino)acetic acid (0.200 g, 0.646 mmol) using General procedure B. Colorless oil (0.231 g, 77% yield). ^1H NMR (400 MHz, DMSO-d_6) δ 8.26 (d, *J* = 7.2 Hz, 1H), 6.38 (d, *J* = 9.7 Hz, 1H), 4.24 – 4.14 (m, 1H), 4.13 – 3.98 (m, 4H), 3.75 (d, *J* = 9.7 Hz, 1H), 2.37 (t, *J* = 7.6 Hz, 2H), 2.05 – 1.75 (m, 5H), 1.67 – 1.42 (m, 12H), 1.38 (s, 9H), 1.21 – 1.12 (m, 6H).

3.7 Diethyl ((*S*)-2-((tert-butoxycarbonyl)amino)-4-phenylbutanoyl)-D-glutamate (7)

Synthesized from **3** (0.151 g, 0.630 mmol) and (*S*)-2-((tert-butoxycarbonyl)amino)-4-phenylbutanoic acid (0.228 g, 0.816 mmol) using General procedure B. Colorless oil (0.200 g, 68% yield). ^1H NMR (400 MHz, DMSO-d_6) δ 8.19 (d, *J* = 7.8 Hz, 1H), 7.30 – 7.23 (m, 2H), 7.23 – 7.15 (m, 3H), 7.00 (d, *J* = 7.8 Hz, 1H), 4.32 – 4.18 (m, 1H), 4.14 – 3.97 (m, 4H), 3.97 – 3.89 (m, 1H), 2.66 – 2.52 (m, 2H), 2.34 (t, *J* = 7.5 Hz, 2H), 2.05 – 1.94 (m, 1H), 1.93 – 1.75 (m, 3H), 1.39 (s, 9H), 1.22 – 1.08 (m, 6H).

3.8 Diethyl ((*S*)-2-(2-((tert-butoxycarbonyl)amino)acetamido)-3-cyclohexylpropanoyl)-D-glutamate (9)

Compound **4** (0.540 g, 1.18 mmol) was deprotected using General procedure A and coupled to (tert-butoxycarbonyl)glycine (0.174 g, 0.996 mmol) using General procedure B to produce the title compound **9** as a yellow oil, which was used in the next step without further purification (0.432 g, 85% yield).

3.9 Diethyl ((*S*)-2-(2-((tert-butoxycarbonyl)amino)acetamido)-3-(pyridin-4-yl)propanoyl)-D-glutamate (10)

Compound **5** (0.218 g, 0.483 mmol) was deprotected using General procedure A and coupled to (tert-butoxycarbonyl)glycine (0.086 g, 0.49 mmol) using General procedure B to produce the title compound **10** as a yellow oil (0.227 g, 93% yield). ^{1}H NMR (400 MHz, DMSO-d_6) δ 8.52 (d, *J* = 7.8 Hz, 1H), 8.43 (d, *J* = 6.0 Hz, 2H), 8.03 (d, *J* = 8.4 Hz, 1H), 7.23 (d, *J* = 6.0 Hz, 2H), 6.93 (t, *J* = 6.1 Hz, 1H), 4.70 – 4.59 (m, 1H), 4.30 – 4.19 (m, 1H), 4.15 – 3.98 (m, 4H), 3.61 – 3.39 (m, 2H), 3.02 – 2.90 (m, 1H), 2.86 – 2.76 (m, 1H), 2.26 (t, *J* = 7.5 Hz, 2H), 1.98 – 1.88 (m, 1H), 1.89 – 1.72 (m, 1H), 1.37 (s, 9H), 1.22 – 1.12 (m, 6H).

3.10 Diethyl ((*S*)-2-(adamantan-1-yl)-2-(2-((tert-butoxycarbonyl)amino)acetamido)acetyl)-D-glutamate (11)

Compound **6** (0.154 g, 0.311 mmol) was deprotected using General procedure A and coupled to (tert-butoxycarbonyl)glycine (0.060 g, 0.34 mmol) using General procedure B to produce the title compound **11** as a colorless oil (0.161 g, 94% yield). ^{1}H NMR (400 MHz, DMSO-d_6) δ 8.53 (d, *J* = 7.3 Hz, 1H), 7.38 (d, *J* = 9.8 Hz, 1H), 7.15 (t, *J* = 6.1 Hz, 1H), 4.26 – 4.13 (m, 2H), 4.13 – 3.97 (m, 4H), 3.60 – 3.52 (m, 2H), 2.39 (t, *J* = 7.5 Hz, 2H), 2.04 – 1.72 (m, 5H), 1.65 – 1.32 (m, 21H), 1.21 – 1.13 (m, 6H).

3.11 Diethyl ((*S*)-2-(2-((tert-butoxycarbonyl)amino)acetamido)-4-phenylbutanoyl)-D-glutamate (12)

Compound **7** (0.149 g, 0.321 mmol) was deprotected using General procedure A and coupled to (tert-butoxycarbonyl)glycine (0.062 g, 0.35 mmol) using General procedure B to produce the title compound **12** as a colorless oil (0.129 g, 77% yield). ^{1}H NMR (400 MHz, DMSO-d_6) δ 8.36 (d, *J* = 7.8 Hz, 1H), 7.95 (d, *J* = 8.1 Hz, 1H), 7.32 – 7.23 (m, 2H), 7.23 – 7.13 (m, 3H), 7.05 (t, *J* = 6.0 Hz, 1H), 4.38 – 4.31 (m, 1H), 4.31 – 4.22 (m, 1H), 4.13 – 3.97 (m, 4H), 3.59 (d, *J* = 6.1 Hz, 2H), 2.61 – 2.53 (m, 2H), 2.35 (t, *J* = 7.5 Hz, 2H), 2.07 – 1.74 (m, 4H), 1.38 (s, 9H), 1.21 – 1.10 (m, 6H).

3.12 Diethyl ((*S*)-3-cyclohexyl-2-(2-((E)-3-(4-hydroxy-3-methoxyphenyl)acrylamido)acetamido)propanoyl)-D-glutamate (14)

Compound **9** (0.432 g, 0.841 mmol) was deprotected using General procedure A and coupled to *trans*-ferulic acid (0.205 g, 1.06 mmol) using General procedure B. Purification by flash chromatography (3% MeOH in DCM) produced the title compound **14** as a yellow solid (0.062 g, 13% yield). ^{1}H NMR (400 MHz, DMSO-d_6) δ 9.45 (s, 1H), 8.32 (d, *J* = 7.7 Hz, 1H), 8.21 (t, *J* = 5.7 Hz, 1H), 8.09 (d, *J* = 8.3 Hz, 1H), 7.32 (d, *J* = 15.7 Hz, 1H), 7.14 (d, *J* = 2.0 Hz, 1H), 7.00 (dd, *J* = 2.0, 8.1 Hz, 1H), 6.80 (d, *J* = 8.1 Hz, 1H), 6.55 (d, *J* = 15.7 Hz, 1H), 4.43 – 4.33 (m, 1H), 4.29 – 4.18 (m, 1H), 4.12 – 3.96 (m, 4H), 3.88 – 3.78 (m, 5H), 2.34 (t, *J* = 7.4 Hz, 2H), 2.05 – 1.92 (m, 1H), 1.91 – 1.77 (m, 1H), 1.75 – 1.55 (m, 5H), 1.55 – 1.35 (m, 2H), 1.33 – 1.08 (m, 10H), 0.95 – 0.78 (m, 2H). ^{13}C NMR (101 MHz, DMSO-d_6) δ 172.69, 172.57, 172.00, 169.24, 166.33, 148.82, 148.27, 139.88, 126.80, 122.04, 119.00, 116.12, 111.32, 61.02, 60.37, 55.98, 51.57, 50.66, 42.82, 33.89, 33.53, 32.40, 31.16, 30.17, 26.51, 26.37, 26.20, 26.06, 14.51, 14.48. UHPLC (254 nm): 94.2%, t_r = 6.26 min. HRMS calcd for $C_{30}H_{44}N_3O_9$ m/z: 590.3072 (M + H)$^+$, found 590.3055.

3.13 Diethyl ((*S*)-2-(2-((*E*)-3-(4-hydroxy-3-methoxyphenyl)acrylamido)acetamido)-3-(pyridin-4-yl)propanoyl)-D-glutamate (15)

Compound **10** (0.174 g, 0.342 mmol) was deprotected using General procedure A and coupled to *trans*-ferulic acid (0.068 g, 0.351 mmol) using General procedure B. Purification by flash chromatography (5% – 10% MeOH in DCM) produced the title compound **15** as a yellow oil (0.042 g, 21% yield). ^{1}H NMR (400 MHz, DMSO-d_6) δ 9.45 (s, 1H), 8.47 (d, *J* = 7.6 Hz, 1H), 8.43 (d, *J* = 5.4 Hz, 2H), 8.29 (d, *J* = 8.7 Hz, 1H), 8.18 (t, *J* = 5.7 Hz, 1H), 7.32 (d, *J* = 15.7 Hz, 1H), 7.24 (d, *J* = 5.4 Hz, 2H), 7.13 (d, *J* = 1.8 Hz, 1H), 7.00 (dd, *J* = 1.8, 8.2 Hz, 1H), 6.79 (d, *J* = 8.2 Hz, 1H), 6.53 (d, *J* = 15.7 Hz, 1H), 4.72 – 4.61 (m, 1H), 4.31 – 4.17 (m, 1H), 4.15 – 3.97 (m, 4H), 3.88 – 3.67 (m, 5H), 3.10 – 2.95 (m, 1H), 2.87 – 2.74 (m, 1H), 2.28 (t, *J* = 7.5 Hz, 2H), 2.05 – 1.90 (m, 1H), 1.90 – 1.74 (m, 1H), 1.24 – 1.11 (m, 6H). ^{13}C NMR (101 MHz, DMSO-d_6) δ 172.53, 171.87, 171.12, 169.36, 166.32, 149.76, 148.83, 148.27, 146.97, 139.95, 126.78, 125.06, 122.04, 118.91, 116.12, 111.35, 61.14, 60.40, 55.98, 53.19, 51.71, 42.70, 40.79, 30.11, 26.49, 14.53, 14.47. UHPLC (254 nm): 95.6%, t_r = 4.21 min. HRMS calcd for $C_{29}H_{37}N_4O_9$ m/z: 585.2555 $(M + H)^+$, found 585.2538.

3.14 Diethyl ((S)-2-(adamantan-1-yl)-2-(2-((*E*)-3-(4-hydroxy-3-methoxyphenyl)acrylamido)acetamido)acetyl)-D-glutamate (16)

Compound **11** (0.118 g, 0.214 mmol) was deprotected using General procedure A and coupled to *trans*-ferulic acid (0.046 g, 0.235 mmol) using General procedure B. Purification by flash chromatography (3% MeOH in DCM) produced the title compound **16** as a yellow oil (0.077 g, 58% yield). ^{1}H NMR (400 MHz, DMSO-d_6) δ 9.45 (s, 1H), 8.50 (d, *J* = 7.2 Hz, 1H), 8.20 (t, *J* = 5.8 Hz, 1H), 7.75 (d, *J* = 9.6 Hz, 1H), 7.34 (d, *J* = 15.7 Hz, 1H), 7.15 (d, *J* = 2.0 Hz, 1H), 7.00 (dd, *J* = 2.0, 8.1 Hz, 1H), 6.79 (d, *J* = 8.1 Hz, 1H), 6.57 (d, *J* = 15.7 Hz, 1H), 4.25 – 4.16 (m, 2H), 4.14 – 3.98 (m, 4H), 3.93 – 3.86 (m, 2H), 3.81 (s, 3H), 2.39 (t, *J* = 7.5 Hz, 2H), 2.02 – 1.76 (m, 5H), 1.69 – 1.41 (m, 12H), 1.20 – 1.12 (m, 6H). ^{13}C NMR (101 MHz, DMSO-d_6) δ 172.50, 172.03, 169.80, 169.16, 166.19, 148.82, 148.28, 139.97, 126.80, 122.10, 119.00, 116.10, 111.31, 60.98, 60.40, 55.99, 51.54, 42.70, 41.37, 40.79, 38.62, 36.87, 36.48, 30.20, 28.20, 26.35, 21.53, 14.53, 14.48. UHPLC (254 nm): 94.2%, t_r = 6.58 min. HRMS calcd for $C_{33}H_{46}N_3O_9$ m/z: 628.3229 $(M + H)^+$, found 628.3209.

3.15 Diethyl ((*S*)-2-(2-((*E*)-3-(4-hydroxy-3-methoxyphenyl)acrylamido)acetamido)-4-phenylbutanoyl)-D-glutamate (17)

Compound **12** (0.100 g, 0.192 mmol) was deprotected using General procedure A and coupled to *trans*-ferulic acid (0.041 g, 0.211 mmol) using General procedure B. Purification by flash chromatography (3% MeOH in DCM) produced the title compound **17** as a white solid (0.085 g, 74% yield). ^{1}H NMR (400 MHz, DMSO-d_6) δ 9.46 (s, 1H), 8.36 – 8.17 (m, 3H), 7.39 – 7.23 (m, 3H), 7.23 – 7.10 (m, 4H), 7.00 (dd, *J* = 2.3, 8.2 Hz, 1H), 6.79 (d, *J* = 8.2 Hz, 1H), 6.57 (d, *J* = 15.7 Hz, 1H), 4.38 – 4.21 (m, 2H), 4.14 – 4.04 (m, 2H), 3.99 (q, *J* = 7.2 Hz, 2H), 3.88 (d, *J* = 5.6 Hz, 2H), 3.81 (s, 3H), 2.66 – 2.53 (m, 2H), 2.34 (t, *J* = 7.5 Hz, 2H), 2.08 – 1.76 (m, 4H), 1.22 – 1.07 (m, 6H). ^{13}C NMR (101 MHz, DMSO-d_6) δ 172.58, 172.01, 171.97, 169.55, 166.43, 148.83, 148.27, 141.80, 139.93, 128.80, 128.73, 126.78, 126.33, 122.06, 118.95, 116.12, 111.33, 61.07, 60.37, 55.98, 52.64, 51.63, 42.98, 34.56, 31.75, 30.20, 26.36, 14.48. UHPLC (254 nm): 92.3%, t_r = 5.97 min. HRMS calcd for $C_{31}H_{40}N_3O_9$ m/z: 598.2759 $(M + H)^+$, found 598.2739.

3.16 Diethyl ((*E*)-3-(p-tolyl)acryloyl)glycyl-L-valyl-D-glutamate (18)

Compound **13** (110 mg, 0.239 mmol) was deprotected using General procedure A and coupled to (*E*)-3-(p-tolyl)acrylic acid (43 mg, 0.263 mmol) using General procedure B. The residue was washed twice with diethyl ether to produce the title compound **18** as a white solid (81 mg, 68% yield). ^{1}H NMR (400 MHz, DMSO-d_6) δ 8.38 (d, *J* = 7.6 Hz, 1H), 8.33 (t, *J* = 5.9 Hz, 1H), 7.93 (d, *J* = 8.9 Hz, 1H), 7.46 (d, *J* = 8.2 Hz, 2H), 7.39 (d, *J* = 15.8 Hz, 1H), 7.23 (d, *J* = 8.2 Hz, 2H), 6.68 (d, *J* = 15.8 Hz, 1H), 4.32 – 4.20 (m, 2H), 4.15 – 3.98 (m, 4H), 3.90 (d, *J* = 6.7 Hz, 2H), 2.39 – 2.29 (m, 5H), 2.05 – 1.94 (m, 2H), 1.90 – 1.76 (m, 1H), 1.25 – 1.09 (m, 6H), 0.85 (t, *J* = 6.5 Hz, 6H). ^{13}C NMR (101 MHz, DMSO-d_6) δ 172.53, 172.01, 171.49, 169.39, 165.92, 139.74, 139.43, 132.55, 130.00, 128.01, 121.22, 61.05, 60.39, 57.84, 51.54, 42.69, 31.22, 30.17, 26.38, 21.42, 19.58, 18.21, 14.53, 14.46. UHPLC (254 nm): 90.0%, t_r = 6.35 min. HRMS calcd for $C_{26}H_{38}N_3O_7$ m/z: 504.2704 $(M + H)^+$, found 504.2700.

3.17 Diethyl ((*E*)-3-(4-(trifluoromethyl)phenyl)acryloyl)glycyl-L-valyl-D-glutamate (19)

Compound **13** (110 mg, 0.239 mmol) was deprotected using General procedure A and coupled to (*E*)-3-(4-(trifluoromethyl)phenyl)acrylic acid (57 mg, 0.263 mmol) using General procedure B. The residue was washed twice with diethyl ether to produce the title compound **19** as a white solid (85 mg, 64% yield). ^{1}H NMR (400 MHz, DMSO-d_6) δ 8.47 (t, *J* = 5.8 Hz, 1H), 8.39 (d, *J* = 7.6 Hz, 1H), 7.98 (d, *J* = 8.9 Hz, 1H), 7.83 – 7.73 (m, 4H), 7.51 (d, *J* = 15.9 Hz, 1H), 6.89 (d, *J* = 15.9 Hz, 1H), 4.32 – 4.20 (m, 2H), 4.13 – 3.97 (m, 4H), 3.93 (d, *J* = 5.9 Hz, 2H), 2.35 (t, *J* = 7.5 Hz, 2H), 2.06 – 1.93 (m, 2H), 1.90 – 1.77 (m, 1H), 1.21 – 1.11 (m, 6H), 0.86 (t, *J* = 6.3 Hz, 6H). ^{13}C NMR (101 MHz, DMSO-d_6) δ 172.54, 172.01, 171.48, 169.20, 165.27, 139.40, 137.79, 129.31, 128.67, 126.30, 126.27, 125.93, 125.11, 61.05, 60.39, 57.87, 51.54, 42.68, 31.24, 30.18, 26.39, 19.59, 18.25, 14.53, 14.46. UHPLC (254 nm): 91.0%, t_r = 6.79 min. HRMS calcd for $C_{26}H_{35}F_3N_3O_7$ m/z: 558.2422 $(M + H)^+$, found 558.2417.

3.18 Diethyl ((*E*)-3-(4-chlorophenyl)acryloyl)glycyl-L-valyl-D-glutamate (20)

Compound **13** (110 mg, 0.239 mmol) was deprotected using General procedure A and coupled to (*E*)-3-(4-chlorophenyl)acrylic acid (48 mg, 0.263 mmol) using General procedure B. White solid (74 mg, 59% yield). ^{1}H NMR (400 MHz, DMSO-d_6) δ 8.42 – 8.33 (m, 2H), 7.96 (d, *J* = 8.9 Hz, 1H), 7.60 (d, *J* = 8.6 Hz, 2H), 7.48 (d, *J* = 8.6 Hz, 2H), 7.42 (d, *J* = 15.8 Hz, 1H), 6.76 (d, *J* = 15.8 Hz, 1H), 4.32 – 4.21 (m, 2H), 4.14 – 3.98 (m, 4H), 3.91 (d, *J* = 5.9 Hz, 2H), 2.35 (t, *J* = 7.5 Hz, 2H), 2.07 – 1.94 (m, 2H), 1.90 – 1.77 (m, 1H), 1.23 – 1.11 (m, 6H), 0.85 (t, *J* = 6.4 Hz, 6H). ^{13}C NMR (101 MHz, DMSO-d_6) δ 172.53, 172.01, 171.48, 169.28, 165.55, 138.11, 134.40, 134.28, 129.74, 129.44, 123.09, 61.05, 60.39, 57.85, 51.54, 42.67, 31.24, 30.17, 26.38, 19.58, 18.23, 14.53, 14.46. UHPLC (254 nm): 92.0%, t_r = 6.52 min. HRMS calcd for $C_{25}H_{35}ClN_3O_7$ m/z: 524.2158 $(M + H)^+$, found 524.2154.

3.19 Diethyl ((*E*)-3-(4-bromophenyl)acryloyl)glycyl-L-valyl-D-glutamate (21)

Compound **13** (150 mg, 0.326 mmol) was deprotected using General procedure A and coupled to (*E*)-3-(4-bromophenyl)acrylic acid (82 mg, 0.358 mmol) using General procedure B. Purification by flash chromatography (3% MeOH in DCM) produced the title compound **21** as a white solid (141 mg, 76% yield).

^{1}H NMR (400 MHz, DMSO-d_6) δ 8.43 – 8.35 (m, 2H), 7.96 (d, J = 8.9 Hz, 1H), 7.63 (d, J = 8.5 Hz, 2H), 7.53 (d, J = 8.5 Hz, 2H), 7.40 (d, J = 15.8 Hz, 1H), 6.77 (d, J = 15.8 Hz, 1H), 4.32 – 4.20 (m, 2H), 4.14 – 3.97 (m, 4H), 3.91 (d, J = 5.9 Hz, 2H), 2.35 (t, J = 7.5 Hz, 2H), 2.06 – 1.93 (m, 2H), 1.91 – 1.76 (m, 1H), 1.21 – 1.11 (m, 6H), 0.85 (t, J = 6.4 Hz, 6H). ^{13}C NMR (101 MHz, DMSO-d_6) δ 172.53, 172.01, 171.47, 169.26, 165.54, 138.19, 134.62, 132.37, 129.98, 123.15, 61.05, 60.39, 57.84, 51.53, 42.67, 31.24, 30.17, 26.38, 19.59, 18.24, 14.53, 14.47. UHPLC (254 nm): 95.0%, t_r = 6.63 min. HRMS calcd for $C_{25}H_{35}BrN_3O_7$ m/z: 568.1653 $(M + H)^+$, found 568.1637.

3.20 Diethyl ((*E*)-3-(4-isopropoxyphenyl)acryloyl)glycyl-L-valyl-D-glutamate (22)

Compound **13** (150 mg, 0.326 mmol) was deprotected using General procedure A and coupled to (*E*)-3-(4-isopropoxyphenyl)acrylic acid (74 mg, 0.358 mmol) using General procedure B. The residue was washed twice with diethyl ether to produce the title compound **22** as a white solid (140 mg, 78% yield). ^{1}H NMR (400 MHz, DMSO-d_6) δ 8.38 (d, J = 7.6 Hz, 1H), 8.28 (t, J = 5.8 Hz, 1H), 7.92 (d, J = 8.9 Hz, 1H), 7.49 (d, J = 8.7 Hz, 2H), 7.36 (d, J = 15.8 Hz, 1H), 6.95 (d, J = 8.7 Hz, 2H), 6.58 (d, J = 15.8 Hz, 1H), 4.66 (p, J = 6.1 Hz, 1H), 4.32 – 4.20 (m, 2H), 4.14 – 3.98 (m, 4H), 3.89 (d, J = 5.7 Hz, 2H), 2.35 (t, J = 7.5 Hz, 2H), 2.05 – 1.93 (m, 2H), 1.91 – 1.75 (m, 1H), 1.27 (d, J = 6.1 Hz, 6H), 1.21 – 1.10 (m, 6H), 0.85 (t, J = 6.6 Hz, 6H). ^{13}C NMR (101 MHz, DMSO-d_6) δ 172.53, 172.01, 171.49, 169.47, 166.12, 159.11, 139.23, 129.67, 127.53, 119.59, 116.25, 69.75, 61.05, 60.38, 57.83, 51.54, 42.69, 31.22, 30.18, 26.38, 22.23, 19.59, 18.20, 14.53, 14.46. UHPLC (254 nm): 91.2%, t_r = 6.70 min. HRMS calcd for $C_{28}H_{42}N_3O_8$ m/z: 548.2966 $(M + H)^+$, found 548.2951.

3.21 Diethyl ((*E*)-3-([1,1'-biphenyl]-4-yl)acryloyl)glycyl-L-valyl-D-glutamate (23)

Compound **13** (100 mg, 0.218 mmol) was deprotected using General procedure A and coupled to (*E*)-3-([1,1'-biphenyl]-4-yl)acrylic acid (54 mg, 0.239 mmol) using General procedure B. The residue was washed twice with diethyl ether to produce the title compound **23** as a yellow solid (100 mg, 81% yield). ^{1}H NMR (400 MHz, DMSO-d_6) δ 8.44 – 8.34 (m, 2H), 7.96 (d, J = 8.9 Hz, 1H), 7.78 – 7.63 (m, 6H), 7.55 – 7.45 (m, 3H), 7.45 – 7.35 (m, 1H), 6.80 (d, J = 15.8 Hz, 1H), 4.33 – 4.20 (m, 2H), 4.14 – 3.99 (m, 4H), 3.92 (d, J = 6.1 Hz, 2H), 2.36 (t, J = 7.4 Hz, 2H), 2.07 – 1.93 (m, 2H), 1.91 – 1.78 (m, 1H), 1.25 – 1.08 (m, 6H), 0.86 (t, J = 6.2 Hz, 6H). ^{13}C NMR (101 MHz, DMSO-d_6) δ 172.54, 172.02, 171.49, 169.36, 165.78, 141.51, 139.81, 138.97, 134.45, 129.49, 128.68, 128.29, 127.61, 127.10, 122.27, 61.06, 60.40, 57.84, 51.53, 42.69, 31.24, 30.18, 26.40, 19.59, 18.24, 14.53, 14.47. UHPLC (254 nm): 95.9%, t_r = 7.18 min. HRMS calcd for $C_{31}H_{40}N_3O_7$ m/z: 566.2861 $(M + H)^+$, found 566.2842.

3.22 Diethyl ((*E*)-3-(4-(4-fluorophenoxy)phenyl)acryloyl)glycyl-L-valyl-D-glutamate (24)

Compound **13** (120 mg, 0.261 mmol) was deprotected using General procedure A and coupled to (*E*)-3-(4-(4-fluorophenoxy)phenyl)acrylic acid (74 mg, 0.287 mmol) using General procedure B. The residue was washed twice with diethyl ether to produce the title compound **24** as a white solid (135 mg, 87% yield). ^{1}H NMR (400 MHz, DMSO-d_6) δ 8.42 – 8.31 (m, 2H), 7.94 (d, J = 9.0 Hz, 1H), 7.59 (d, J = 8.6 Hz, 2H), 7.41 (d, J = 15.8 Hz, 1H), 7.32 – 7.22 (m, 2H), 7.18 – 7.08 (m, 2H), 7.00 (d, J = 8.6 Hz, 2H), 6.65 (d, J = 15.8 Hz, 1H), 4.30 – 4.21 (m, 2H), 4.15 – 3.97 (m, 4H), 3.90 (d, J = 6.1 Hz, 2H), 2.35 (t, J = 7.5 Hz, 2H), 2.04 – 1.93

(m, 2H), 1.91 – 1.78 (m, 1H), 1.22 – 1.11 (m, 6H), 0.85 (t, *J* = 6.5 Hz, 6H). ^{13}C NMR (101 MHz, DMSO-*d*$_6$) δ 172.53, 172.01, 171.48, 169.38, 165.84, 160.21, 158.84, 157.82, 152.30, 138.69, 130.33, 129.93, 121.85, 121.77, 121.18, 118.46, 117.32, 117.09, 61.05, 60.39, 57.82, 51.54, 42.66, 31.24, 30.17, 26.38, 19.59, 18.22, 14.53, 14.47. UHPLC (254 nm): 95.9%, t_r = 7.27 min. HRMS calcd for $C_{31}H_{39}FN_3O_8$ m/z: 600.2716 (M + H)$^+$, found 600.2697.

3.23 Diethyl ((*E*)-3-(3-nitrophenyl)acryloyl)glycyl-L-valyl-D-glutamate (25)

Compound **13** (150 mg, 0.326 mmol) was deprotected using General procedure A and coupled to (*E*)-3-(3-nitrophenyl)acrylic acid (69 mg, 0.358 mmol) using General procedure B. Purification by flash chromatography (4% MeOH in DCM) produced the title compound **25** as a yellow solid (112 mg, 64% yield). ^{1}H NMR (400 MHz, DMSO-*d*$_6$) δ 8.47 – 8.37 (m, 3H), 8.22 (dd, *J* = 2.7, 8.2 Hz, 1H), 8.08 – 7.96 (m, 2H), 7.72 (t, *J* = 8.0 Hz, 1H), 7.57 (d, *J* = 15.8 Hz, 1H), 6.98 (d, *J* = 15.8 Hz, 1H), 4.32 – 4.22 (m, 2H), 4.13 – 4.00 (m, 4H), 3.94 (d, *J* = 5.7 Hz, 2H), 2.36 (t, *J* = 7.5 Hz, 2H), 2.05 – 1.94 (m, 2H), 1.92 – 1.78 (m, 1H), 1.22 – 1.11 (m, 6H), 0.86 (t, *J* = 6.2 Hz, 6H). ^{13}C NMR (101 MHz, DMSO-*d*$_6$) δ 172.54, 172.02, 171.48, 169.16, 165.16, 148.77, 137.23, 137.14, 134.35, 130.98, 125.22, 124.26, 122.14, 61.05, 60.39, 57.86, 51.54, 42.69, 31.24, 30.18, 26.39, 19.59, 18.25, 14.53, 14.46. UHPLC (254 nm): 97.3%, t_r = 5.99 min. HRMS calcd for $C_{25}H_{35}N_4O_9$ m/z: 535.2399 (M + H)$^+$, found 535.2384.

3.24 Diethyl ((*E*)-3-(3-bromophenyl)acryloyl)glycyl-L-valyl-D-glutamate (26)

Compound **13** (150 mg, 0.326 mmol) was deprotected using General procedure A and coupled to (*E*)-3-(3-bromophenyl)acrylic acid (82 mg, 0.358 mmol) using General procedure B. Purification by flash chromatography (4% MeOH in DCM) produced the title compound **26** as a white solid (121 mg, 65% yield). ^{1}H NMR (400 MHz, DMSO-*d*$_6$) δ 8.42 – 8.31 (m, 2H), 7.97 (d, *J* = 8.9 Hz, 1H), 7.80 – 7.78 (m, 1H), 7.63 – 7.54 (m, 2H), 7.45 – 7.32 (m, 2H), 6.81 (d, *J* = 15.9 Hz, 1H), 4.34 – 4.19 (m, 2H), 4.14 – 3.98 (m, 4H), 3.92 (d, *J* = 5.9 Hz, 2H), 2.35 (t, *J* = 7.6 Hz, 2H), 2.06 – 1.92 (m, 2H), 1.92 – 1.77 (m, 1H), 1.22 – 1.12 (m, 6H), 0.85 (t, *J* = 6.4 Hz, 6H). ^{13}C NMR (101 MHz, DMSO-*d*$_6$) δ 172.54, 172.02, 171.48, 169.23, 165.38, 137.92, 137.82, 132.50, 131.51, 130.57, 126.91, 123.98, 122.73, 61.05, 60.39, 57.85, 51.54, 42.68, 31.22, 30.18, 26.38, 19.59, 18.23, 14.53, 14.47. UHPLC (254 nm): 95.1%, t_r = 6.59 min. HRMS calcd for $C_{25}H_{35}BrN_3O_7$ m/z: 568.1653 (M + H)$^+$, found 568.1637.

3.25 Diethyl ((*E*)-3-([1,1'-biphenyl]-3-yl)acryloyl)glycyl-L-valyl-D-glutamate (27)

Compound **13** (150 mg, 0.326 mmol) was deprotected using General procedure A and coupled to (*E*)-3-([1,1'-biphenyl]-3-yl)acrylic acid (81 mg, 0.358 mmol) using General procedure B. Purification by flash chromatography (5% MeOH in DCM) produced the title compound **27** as a white solid (152 mg, 83% yield). ^{1}H NMR (400 MHz, DMSO-*d*$_6$) δ 8.39 (d, *J* = 7.6 Hz, 1H), 8.35 (t, *J* = 5.7 Hz, 1H), 7.97 (d, *J* = 9.0 Hz, 1H), 7.86 (s, 1H), 7.74 – 7.63 (m, 3H), 7.61 – 7.47 (m, 5H), 7.45 – 7.36 (m, 1H), 6.88 (d, *J* = 15.9 Hz, 1H), 4.32 – 4.21 (m, 2H), 4.13 – 3.98 (m, 4H), 3.93 (d, *J* = 5.9 Hz, 2H), 2.36 (t, *J* = 7.5 Hz, 2H), 2.06 – 1.93 (m, 2H), 1.91 – 1.78 (m, 1H), 1.25 – 1.08 (m, 6H), 0.86 (t, *J* = 6.2 Hz, 6H). ^{13}C NMR (101 MHz, DMSO-*d*$_6$) δ 172.54, 172.02, 171.49, 169.32, 165.75, 141.27, 140.13, 139.35, 136.01, 130.04, 129.44, 128.28, 128.21, 127.24, 126.84, 126.56,

122.80, 61.05, 60.39, 57.85, 51.55, 42.72, 31.23, 30.19, 26.39, 19.59, 18.23, 14.53, 14.46. UHPLC (254 nm): 94.1%, t_r = 7.21 min. HRMS calcd for $C_{31}H_{40}N_3O_7$ m/z: 566.2861 $(M + H)^+$, found 566.2844.

3.26 Diethyl ((*E*)-3-(3-phenoxyphenyl)acryloyl)glycyl-L-valyl-D-glutamate (28)

Compound **13** (150 mg, 0.326 mmol) was deprotected using General procedure A and coupled to (*E*)-3-(3-phenoxyphenyl)acrylic acid (86 mg, 0.359 mmol) using General procedure B. The residue was washed twice with diethyl ether to produce the title compound **28** as a white solid (157 mg, 83% yield). ^1H NMR (400 MHz, DMSO-d_6) δ 8.37 (dd, *J* = 3.6, 6.8 Hz, 2H), 7.95 (d, *J* = 8.9 Hz, 1H), 7.48 – 7.31 (m, 5H), 7.26 – 7.13 (m, 2H), 7.11 – 6.97 (m, 3H), 6.71 (d, *J* = 15.8 Hz, 1H), 4.31 – 4.20 (m, 2H), 4.13 – 3.97 (m, 4H), 3.89 (d, *J* = 7.1 Hz, 2H), 2.35 (t, *J* = 7.5 Hz, 2H), 2.06 – 1.92 (m, 2H), 1.88 – 1.77 (m, 1H), 1.22 – 1.08 (m, 6H), 0.84 (t, *J* = 6.5 Hz, 6H). ^{13}C NMR (101 MHz, DMSO-d_6) δ 172.54, 172.01, 171.49, 169.28, 165.56, 157.64, 156.81, 138.71, 137.38, 131.08, 130.62, 124.19, 123.42, 123.16, 120.05, 119.27, 117.38, 61.05, 60.38, 57.85, 51.54, 42.66, 31.21, 30.17, 26.38, 19.59, 18.23, 14.53, 14.46. UHPLC (254 nm): 96.5%, t_r = 7.23 min. HRMS calcd for $C_{31}H_{40}N_3O_8$ m/z: 582.2810 $(M + H)^+$, found 582.2807.

3.27 Diethyl ((*E*)-3-(benzo[d][1,3]dioxol-5-yl)acryloyl)glycyl-L-valyl-D-glutamate (29)

Compound **13** (120 mg, 0.261 mmol) was deprotected using General procedure A and coupled to (*E*)-3-(benzo[d][1,3]dioxol-5-yl)acrylic acid (55 mg, 0.287 mmol) using General procedure B. Purification by flash chromatography (2% MeOH in DCM) produced the title compound **29** as a yellow solid (123 mg, 88% yield). ^1H NMR (400 MHz, DMSO-d_6) δ 8.39 (d, *J* = 7.6 Hz, 1H), 8.24 (t, *J* = 5.9 Hz, 1H), 7.93 (d, *J* = 9.0 Hz, 1H), 7.34 (d, *J* = 15.8 Hz, 1H), 7.16 (s, 1H), 7.07 (d, *J* = 8.1 Hz, 1H), 6.95 (d, *J* = 8.1 Hz, 1H), 6.58 (d, *J* = 15.8 Hz, 1H), 6.07 (s, 2H), 4.34 – 4.19 (m, 2H), 4.15 – 3.97 (m, 4H), 3.89 (d, *J* = 6.0 Hz, 2H), 2.35 (t, *J* = 7.5 Hz, 2H), 2.08 – 1.93 (m, 2H), 1.91 – 1.77 (m, 1H), 1.21 – 1.11 (m, 6H), 0.85 (t, *J* = 6.6 Hz, 6H). ^{13}C NMR (101 MHz, DMSO-d_6) δ 172.53, 172.01, 171.48, 169.40, 165.97, 148.98, 148.41, 139.30, 129.70, 123.82, 120.34, 109.05, 106.68, 101.91, 61.05, 60.39, 57.82, 51.53, 42.69, 31.24, 30.17, 26.38, 19.58, 18.22, 14.53, 14.46. UHPLC (254 nm): 97.2%, t_r = 5.82 min. HRMS calcd for $C_{26}H_{36}N_3O_9$ m/z: 534.2446 $(M + H)^+$, found 534.2442.

3.28 Diethyl (benzofuran-2-carbonyl)glycyl-L-valyl-D-glutamate (30)

Compound **13** (150 mg, 0.326 mmol) was deprotected using General procedure A and coupled to benzofuran-2-carboxylic acid (58 mg, 0.358 mmol) using General procedure B. Purification by flash chromatography (3% MeOH in DCM) produced the title compound **30** as a white solid (101 mg, 62% yield). ^1H NMR (400 MHz, DMSO-d_6) δ 8.89 (t, *J* = 6.0 Hz, 1H), 8.40 (d, *J* = 7.7 Hz, 1H), 8.00 (d, *J* = 8.9 Hz, 1H), 7.79 (d, *J* = 7.8 Hz, 1H), 7.67 (d, *J* = 8.3 Hz, 1H), 7.56 (d, *J* = 0.9 Hz, 1H), 7.50 – 7.45 (m, 1H), 7.37 – 7.32 (m, 1H), 4.33 – 4.21 (m, 2H), 4.14 – 3.93 (m, 6H), 2.35 (t, *J* = 7.5 Hz, 2H), 2.05 – 1.93 (m, 2H), 1.90 – 1.78 (m, 1H), 1.21 – 1.11 (m, 6H), 0.86 (t, *J* = 6.1 Hz, 6H). ^{13}C NMR (101 MHz, DMSO-d_6) δ 172.51, 172.02, 171.46, 168.92, 158.82, 154.69, 149.33, 127.59, 127.38, 124.21, 123.28, 112.27, 110.11, 61.06, 60.38, 57.90, 51.52, 42.45, 31.34, 30.17, 26.41, 19.59, 18.30, 14.50, 14.46. UHPLC (254 nm): 98.2%, t_r = 5.99 min. HRMS calcd for $C_{25}H_{34}N_3O_8$ m/z: 504.2340 $(M + H)^+$, found 504.2325.

3.29 Diethyl (6-bromobenzofuran-2-carbonyl)glycyl-L-valyl-D-glutamate (31)

Compound **13** (120 mg, 0.261 mmol) was deprotected using General procedure A and coupled to 6-bromobenzofuran-2-carboxylic acid (69 mg, 0.287 mmol) using General procedure B. The residue was washed twice with diethyl ether to produce the title compound **31** as a white solid (94 mg, 62% yield). ^{1}H NMR (400 MHz, DMSO-d_6) δ 8.92 (t, *J* = 6.0 Hz, 1H), 8.40 (d, *J* = 7.6 Hz, 1H), 8.04 – 7.95 (m, 2H), 7.76 (d, *J* = 8.4 Hz, 1H), 7.59 (d, *J* = 0.9 Hz, 1H), 7.52 (dd, *J* = 1.7, 8.4 Hz, 1H), 4.33 – 4.21 (m, 2H), 4.13 – 3.91 (m, 6H), 2.35 (t, *J* = 7.5 Hz, 2H), 2.06 – 1.93 (m, 2H), 1.91 – 1.74 (m, 1H), 1.24 – 1.09 (m, 6H), 0.86 (t, *J* = 6.1 Hz, 6H). ^{13}C NMR (101 MHz, DMSO-d_6) δ 172.51, 172.01, 171.45, 168.82, 158.49, 154.99, 149.95, 127.53, 126.96, 124.84, 119.84, 115.44, 110.00, 61.06, 60.38, 57.92, 51.52, 42.44, 31.34, 30.17, 26.41, 19.59, 18.31, 14.53, 14.45. UHPLC (254 nm): 91.9%, t_r = 6.67 min. HRMS calcd for $C_{25}H_{33}BrN_3O_8$ m/z: 582.1446 $(M + H)^+$, found 582.1428.

3.30 Diethyl (5-bromobenzofuran-2-carbonyl)glycyl-L-valyl-D-glutamate (32)

Compound **13** (120 mg, 0.261 mmol) was deprotected using General procedure A and coupled to 5-bromobenzofuran-2-carboxylic acid (69 mg, 0.287 mmol) using General procedure B. The residue was washed twice with diethyl ether to produce the title compound **32** as a yellow solid (101 mg, 66% yield). ^{1}H NMR (400 MHz, DMSO-d_6) δ 8.97 (t, *J* = 6.0 Hz, 1H), 8.40 (d, *J* = 7.7 Hz, 1H), 8.06 – 7.97 (m, 2H), 7.69 – 7.58 (m, 2H), 7.54 (s, 1H), 4.31 – 4.22 (m, 2H), 4.13 – 3.94 (m, 6H), 2.35 (t, *J* = 7.5 Hz, 2H), 2.05 – 1.93 (m, 2H), 1.89 – 1.75 (m, 1H), 1.22 – 1.10 (m, 6H), 0.86 (t, *J* = 6.1 Hz, 6H). ^{13}C NMR (101 MHz, DMSO-d_6) δ 172.51, 172.02, 171.46, 168.81, 158.44, 153.52, 150.49, 129.98, 129.83, 125.73, 116.42, 114.41, 109.52, 61.06, 60.38, 57.92, 51.51, 42.44, 31.35, 30.17, 26.41, 19.59, 18.32, 14.52, 14.45. UHPLC (254 nm): 93.9%, t_r = 6.68 min. HRMS calcd for $C_{25}H_{33}BrN_3O_8$ m/z: 582.1446 $(M + H)^+$, found 582.1429.

3.31 Diethyl (5-nitrobenzofuran-2-carbonyl)glycyl-L-valyl-D-glutamate (33)

Compound **13** (110 mg, 0.239 mmol) was deprotected using General procedure A and coupled to 5-nitrobenzofuran-2-carboxylic acid (54 mg, 0.263 mmol) using General procedure B. Purification by flash chromatography (3% MeOH in DCM) produced the title compound **33** as a yellow solid (75 mg, 57% yield). ^{1}H NMR (400 MHz, DMSO-d_6) δ 9.11 (t, *J* = 6.0 Hz, 1H), 8.80 (d, *J* = 2.8 Hz, 1H), 8.41 (d, *J* = 7.6 Hz, 1H), 8.34 (dd, *J* = 2.7, 9.0 Hz, 1H), 8.04 (d, *J* = 8.9 Hz, 1H), 7.93 (d, *J* = 9.2 Hz, 1H), 7.79 (s, 1H), 4.34 – 4.21 (m, 2H), 4.15 – 3.95 (m, 6H), 2.36 (t, *J* = 7.5 Hz, 2H), 2.06 – 1.91 (m, 2H), 1.91 – 1.77 (m, 1H), 1.24 – 1.12 (m, 6H), 0.89 – 0.83 (m, 6H). ^{13}C NMR (101 MHz, DMSO-d_6) δ 172.51, 172.02, 171.46, 168.71, 158.11, 157.38, 152.06, 144.66, 128.22, 122.71, 120.09, 113.39, 110.89, 61.07, 60.40, 57.93, 51.52, 42.45, 31.36, 30.17, 26.43, 19.59, 18.33, 14.52, 14.45. UHPLC (254 nm): 98.9%, t_r = 6.01 min. HRMS calcd for $C_{25}H_{33}N_4O_{10}$ m/z: 549.2191 $(M + H)^+$, found 549.2189.

3.32 Diethyl (5-methyl-2-phenyloxazole-4-carbonyl)glycyl-L-valyl-D-glutamate (34)

Compound **13** (100 mg, 0.218 mmol) was deprotected using General procedure A and coupled to 5-methyl-2-phenyloxazole-4-carboxylic acid (49 mg, 0.239 mmol) using General procedure B. The residue was washed twice with diethyl ether to produce the title compound **34** as a white solid (85 mg, 71% yield).

^{1}H NMR (400 MHz, DMSO-d_6) δ 8.40 (d, J = 7.7 Hz, 1H), 8.28 (t, J = 5.9 Hz, 1H), 8.03 – 7.97 (m, 3H), 7.59 – 7.53 (m, 3H), 4.32 – 4.22 (m, 2H), 4.14 – 3.99 (m, 4H), 3.96 (d, J = 5.9 Hz, 2H), 2.65 (s, 3H), 2.35 (t, J = 7.4 Hz, 2H), 2.05 – 1.93 (m, 2H), 1.89 – 1.74 (m, 1H), 1.22 – 1.09 (m, 6H), 0.86 (t, J = 6.4 Hz, 6H). ^{13}C NMR (101 MHz, DMSO-d_6) δ 172.51, 172.00, 171.45, 169.07, 161.66, 158.34, 153.21, 131.41, 130.44, 129.71, 126.71, 126.41, 61.06, 60.38, 57.92, 51.51, 42.23, 31.33, 30.17, 26.42, 19.59, 18.28, 14.52, 14.45, 11.91. UHPLC (254 nm): 95.9%, t_r = 6.79 min. HRMS calcd for $C_{27}H_{37}N_4O_8$ m/z: 545.2606 $(M + H)^+$, found 545.2589.

3.33 Dicyclopentyl ((*E*)-3-([1,1'-biphenyl]-3-yl)acryloyl)glycyl-L-valyl-D-glutamate (38)

Compound **37** (93 mg, 0.17 mmol) was deprotected using General procedure A and coupled to (*E*)-3-([1,1'-biphenyl]-3-yl)acrylic acid (42 mg, 0.19 mmol) using General procedure B. Purification by flash chromatography (30% – 50% EtOAc in hexanes) produced the title compound **38** as a white solid (33 mg, 30% yield). ^{1}H NMR (400 MHz, DMSO-d_6) δ 8.41 – 8.27 (m, 2H), 7.98 (d, J = 8.7 Hz, 1H), 7.86 (s, 1H), 7.75 – 7.64 (m, 3H), 7.58 (d, J = 7.6 Hz, 1H), 7.54 – 7.47 (m, 4H), 7.43 – 7.36 (m, 1H), 6.88 (d, J = 15.8 Hz, 1H), 5.13 – 4.97 (m, 2H), 4.30 – 4.14 (m, 2H), 3.92 (d, J = 6.2 Hz, 2H), 2.37 – 2.26 (m, 2H), 2.02 – 1.89 (m, 2H), 1.88 – 1.72 (m, 5H), 1.69 – 1.44 (m, 12H), 0.89 – 0.82 (m, 6H). ^{13}C NMR (101 MHz, DMSO-d_6) δ 172.19, 171.77, 171.46, 169.23, 165.71, 141.27, 140.13, 139.32, 136.02, 130.04, 129.45, 128.27, 128.21, 127.24, 126.86, 126.54, 122.82, 77.65, 76.91, 57.84, 51.63, 42.71, 32.63, 32.61, 32.58, 32.48, 31.27, 30.41, 26.37, 23.71, 23.65, 19.57, 18.33. UHPLC (254 nm): 96.0%, t_r = 8.63 min. HRMS calcd for $C_{37}H_{48}N_3O_7$ m/z: 646.3487 $(M + H)^+$, found 646.3480.

3.34 Dicyclopentyl ((*E*)-3-(3-phenoxyphenyl)acryloyl)glycyl-L-valyl-D-glutamate (39)

Compound **37** (93 mg, 0.17 mmol) was deprotected using General procedure A and coupled to (*E*)-3-(3-phenoxyphenyl)acrylic acid (45 mg, 0.19 mmol) using General procedure B. Purification by flash chromatography (3% MeOH in DCM) produced the title compound **39** as a white solid (39 mg, 34% yield). ^{1}H NMR (400 MHz, DMSO-d_6) δ 8.41 – 8.26 (m, 2H), 7.96 (d, J = 9.0 Hz, 1H), 7.50 – 7.31 (m, 5H), 7.25 – 7.12 (m, 2H), 7.09 – 6.96 (m, 3H), 6.72 (d, J = 15.8 Hz, 1H), 5.12 – 4.97 (m, 2H), 4.29 – 4.15 (m, 2H), 3.89 (d, J = 6.4 Hz, 2H), 2.30 (t, J = 7.4 Hz, 2H), 2.02 – 1.87 (m, 2H), 1.86 – 1.70 (m, 5H), 1.67 – 1.44 (m, 12H), 0.89 – 0.78 (m, 6H). ^{13}C NMR (101 MHz, DMSO-d_6) δ 172.17, 171.76, 171.46, 169.18, 165.51, 157.63, 156.81, 138.68, 137.39, 131.07, 130.61, 124.18, 123.42, 123.19, 120.04, 119.25, 117.38, 77.65, 76.91, 57.84, 51.62, 42.66, 32.61, 32.59, 32.48, 31.25, 30.41, 26.37, 23.71, 23.66, 19.56, 18.33. UHPLC (254 nm): 97.7%, t_r = 8.62 min. HRMS calcd for $C_{37}H_{48}N_3O_8$ m/z: 662.3436 $(M + H)^+$, found 662.3428.

3.35 Diethyl ((*E*)-3-(4-(2-((adamantan-1-yl)acetoxy)-3-methoxyphenyl)acryloyl)glycyl-L-valyl-D-glutamate (40)

Synthesized from **1** (33 mg, 0.062 mmol) and 1-adamantaneacetic acid (14 mg, 0.074 mmol) using General procedure B. The residue was washed twice with diethyl ether to produce the title compound **40** as a white solid (25 mg, 57% yield). ^{1}H NMR (400 MHz, DMSO-d_6) δ 8.40 (d, J = 7.6 Hz, 1H), 8.32 (t, J = 5.9 Hz, 1H), 7.95 (d, J = 9.0 Hz, 1H), 7.43 (d, J = 15.8 Hz, 1H), 7.34 (s, 1H), 7.17 (d, J = 8.2 Hz, 1H), 7.09 (d, J = 8.2 Hz, 1H), 6.76 (d, J = 15.8 Hz, 1H), 4.34 – 4.19 (m, 2H), 4.17 – 3.97 (m, 4H), 3.91 (d, J = 5.9 Hz, 2H), 3.82 (s,

3H), 2.35 (t, *J* = 7.5 Hz, 2H), 2.29 (s, 2H), 2.07 – 1.91 (m, 5H), 1.91 – 1.78 (m, 1H), 1.77 – 1.59 (m, 12H), 1.23 – 1.11 (m, 6H), 0.85 (t, *J* = 6.5 Hz, 6H). ^{13}C NMR (101 MHz, DMSO-d_6) δ 172.53, 172.02, 171.48, 169.29, 169.15, 165.70, 151.51, 140.68, 138.88, 134.30, 123.81, 122.58, 120.62, 112.10, 61.05, 60.40, 57.82, 56.17, 51.54, 48.25, 42.70, 42.07, 36.74, 33.06, 31.27, 30.18, 28.48, 26.40, 19.59, 18.24, 14.53, 14.47. UHPLC (254 nm): 97.3%, t_r = 8.96 min. HRMS calcd for $C_{38}H_{54}N_3O_{10}$ m/z: 712.3809 (M + H)$^+$, found 712.3779.

3.36 Dicyclopentyl ((*E*)-3-(4-(2-(adamantan-1-yl)acetoxy)-3-methoxyphenyl)acryloyl)glycyl-L-valyl-D-glutamate (41)

Synthesized from **2** (90 mg, 0.15 mmol) and 1-adamantaneacetic acid (31 mg, 0.16 mmol) using General procedure B. Purification by flash chromatography (10% – 25% EtOAc in hexanes) produced the title compound **41** as a white solid (50 mg, 43% yield). ^{1}H NMR (400 MHz, DMSO-d_6) δ 8.38 (d, *J* = 7.5 Hz, 1H), 8.30 (t, *J* = 5.7 Hz, 1H), 7.97 (d, *J* = 9.2 Hz, 1H), 7.43 (d, *J* = 15.6 Hz, 1H), 7.34 (d, *J* = 1.6 Hz, 1H), 7.17 (dd, *J* = 1.6, 8.2 Hz, 1H), 7.09 (d, *J* = 8.2 Hz, 1H), 6.78 (d, *J* = 15.8 Hz, 1H), 5.11 – 4.98 (m, 2H), 4.31 – 4.22 (m, 1H), 4.26 – 4.14 (m, 1H), 3.91 (d, *J* = 5.6 Hz, 2H), 3.82 (s, 3H), 2.38 – 2.24 (m, 4H), 2.01 – 1.90 (m, 5H), 1.85 – 1.47 (m, 29H), 0.85 (t, *J* = 6.1 Hz, 6H). ^{13}C NMR (101 MHz, DMSO-d_6) δ 172.16, 171.76, 171.46, 169.20, 169.13, 165.66, 151.51, 140.67, 138.85, 134.32, 123.79, 122.61, 120.62, 112.08, 77.65, 76.92, 57.82, 56.16, 51.62, 48.88, 48.25, 42.26, 42.07, 36.79, 36.74, 33.05, 32.62, 32.59, 32.48, 32.33, 31.30, 30.41, 28.50, 28.43, 26.38, 23.72, 23.66, 19.56, 18.33. UHPLC (254 nm): 90.4%, t_r = 10.15 min. HRMS calcd for $C_{44}H_{62}N_3O_{10}$ m/z: 792.4435 (M + H)$^+$, found 792.4423.

4 Biology

4.1 HEK-Blue NOD1 and NOD2 cells

HEK-Blue NOD1 and NOD2 cells (Invivogen, San Diego, CA, USA) were cultured according to the manufacturer instructions in Dulbecco's modified Eagle's medium (Sigma-Aldrich, St. Louis, MO, USA) supplemented with 10% heat-inactivated fetal bovine serum (Gibco), 2 mM L-glutamine (Sigma-Aldrich), 100 U/mL penicillin (Sigma-Aldrich), 100 µg/mL streptomycin (Sigma-Aldrich), and 100 µg/mL Normocin (Invivogen) for two passages. All subsequent passages were cultured in medium additionally supplemented with 100 µg/mL Zeocin and 30 µg/mL Blasticidin (Invivogen). The cells were incubated in a humidified atmosphere at 37 °C and 5% CO_2.

4.2 RAW-Blue cells

RAW-Blue cells (Invivogen, San Diego, CA, USA) were cultured according to the manufacturer instructions in Dulbecco's modified Eagle's medium (Sigma-Aldrich, St. Louis, MO, USA) supplemented with 10% heat-inactivated fetal bovine serum (Gibco), 2 mM L-glutamine (Sigma-Aldrich), 100 U/mL penicillin (Sigma-Aldrich), 100 µg/mL streptomycin (Sigma-Aldrich), and 100 µg/mL Normocin (Invivogen). After the first

two passages, 200 µg/mL Zeocin (Invivogen) was added to the medium every other passage to maintain selection pressure. The cells were incubated in a humidified atmosphere at 37 °C and 5% CO_2.

4.3 Peripheral blood mononuclear cells (PBMCs)

Human PBMCs from healthy and consenting donors were isolated from heparinized blood by density gradient centrifugation with Ficoll-Paque (Pharmacia, Sweden). The isolated cells were washed twice with PBS, resuspended in RPMI 1640 medium (Sigma-Aldrich, St. Louis, MO, USA) supplemented with 10% heat-inactivated fetal bovine serum (Gibco), 2 mM L-glutamine (Sigma-Aldrich), 100 U/mL penicillin (Sigma-Aldrich), and 100 µg/mL streptomycin (Sigma-Aldrich), and used in the assays.

4.4 K562 cells

K562 cells are a chronic myelogenous leukemia cell line (ATCC, Manassas, VA, USA).[4] K562 cells were cultured in RPMI 1640 medium (Sigma-Aldrich, St. Louis, MO, USA) supplemented with 10% heat-inactivated fetal bovine serum (Gibco), 2 mM L-glutamine (Sigma-Aldrich), 100 U/mL penicillin (Sigma-Aldrich), and 100 µg/mL streptomycin (Sigma-Aldrich).

4.5 Cytotoxicity

The tested compounds were dissolved in DMSO and further diluted in culture medium to the desired final concentrations, such that the final DMSO concentration never exceeded 0.1%. HEK-Blue NOD2 cells were seeded (40,000 cells/well) in 96-well plates in 100 µL culture medium, and treated with 2 µM of each compound or with the corresponding vehicle (0.1% DMSO; control cells). After 18 h of incubation (37 °C, 5 % CO_2), the metabolic activity was assessed using the CellTiter 96 Aqueous One Solution cell proliferation assay (Promega, Madison, WI, USA), according to the manufacturer instructions. The experiments were run in duplicates, and repeated as three independent biological replicates.

4.6 NOD1/NOD2-NF-kB reporter assay

HEK-Blue NOD2 or NOD1 cells were seeded (25,000 cells/well) in 96-well plates in 100 µL HEK-Blue detection medium (Invivogen, San Diego, CA, USA) and treated with compounds (2 µM for fixed concentration assay; 8-16 different concentrations from 0.1 nM to 20 µM for EC_{50} determination) or with the corresponding vehicle (0.1% DMSO). After 18 h of incubation (37 °C, 5% CO_2), secreted embryonic alkaline phosphatase (SEAP) activity was determined spectrophotometrically as absorbance at 630 nm (BioTek Synergy microplate reader; Winooski, VT, USA). EC_{50} values were calculated using Prism software (version 9; GraphPad Software, CA, USA). The experiments were run in duplicates and repeated as at least three independent biological replicates.

For determination of specificity, HEK-Blue NOD2 cells (25,000 cells/well) were first pre-treated for 1 h with the NOD2 antagonist **SG84** (10 µM),[3] before the addition of compounds (2 µM), with incubation for 18 h. SEAP activity in the supernatants was determined as above. The experiments were run in duplicates and repeated as two independent biological replicates.

4.7 Measurement of NF-κB transcriptional activity in RAW-Blue cells

RAW-Blue cells were seeded (100,000 cells/well) in 96-well plates in 200 µL growth medium (without Normocin and Zeocin) and treated with MDP (1 µM) or **40** (1 µM), in the presence or absence of LPS (10 ng/mL), or the corresponding vehicle (0.1% DMSO). In some wells, the cells were pre-treated for 1 h with the NOD2 antagonist **SG84** (10 µM), before the addition of other immunostimulants. After 18 h of incubation (37 °C, 5% CO_2), 10 µL of the induced supernatant was added to 90 µL QUANTI-Blue solution (Invivogen). After 2 h of incubation (37 °C), SEAP activity was determined spectrophotometrically as absorbance at 630 nm (BioTek Synergy microplate reader; Winooski, VT, USA). The experiments were run in duplicates and repeated as four independent biological replicates.

4.8 Peripheral blood mononuclear cell cytotoxicity

The PBMC cytotoxicity assays using K562 cells were performed as described previously, with some modifications.[2] PBMCs were seeded (400,000 cells/well) in duplicates in 96-well U-bottom plates and treated with **2** (1 µM) or **40** (1 µM), in the presence or absence of LPS (1 µg/mL) or IFN-γ (200 U/mL), IL-2 (200 U/mL), or the corresponding vehicle (0.1% DMSO) for 18 h. K562 were stained with carboxyfluorescein succinimidyl ester (CFSE, Invitrogen, Carlsbad, CA, USA), washed twice with complete medium, and added (10,000 cells/well) to the pre-treated PBMCs for a final effector cell to target tumor cell ratio of 40:1. After a 4 h coincubation (37 °C, 5% CO2), cells were stained with Sytox Blue dead cell stain (Invitrogen) and analyzed using an Attune NxT flow cytometer (Thermo Fisher Scientific, Waltham, MA, USA) and FlowJo software (Tree Star, Inc., Ashland, OR, USA). Cells that were positive for both CFSE and Sytox Blue were defined as dead K562 cells. PBMCs alone and CFSE-labeled K562 cells alone were also treated with the compounds at the same concentrations and stained with Sytox Blue to exclude any direct cytotoxicity of the compounds towards the PBMCs and cancer cells.

4.9 Cytokine release from peripheral blood mononuclear cells

Peripheral blood mononuclear cells were seeded (500,000 cells/well) in 24-well plates in 500 µL growth medium and treated with MDP (1 µM), **2** (1 µM), or **40** (1 µM), in the presence or absence of LPS (10 ng/mL) or IFN-γ (200 U/mL), or the corresponding vehicle (0.1% DMSO). In some wells, PBMCs were pre-treated for 1 h with the NOD2 antagonist **SG84** (10 µM), before the addition of **40** (1 µM). Cell-free supernatants were collected after 18 h of incubation (37 °C, 5% CO_2) and stored at -80 °C until tested. Cytokine production was determined with BD Cytometric Bead Array human inflammatory cytokines kit (BD Bioscience) on an Attune NxT flow cytometer (Thermo Fisher Scientific, Waltham, MA, USA). Standard curves were generated using recombinant cytokines contained in the kit. The data were analyzed using the FlowJo (Tree Star, Inc., Ashland, OR, USA) and Prism (GraphPad, San Diego, CA, USA) software.

4.10 Statistics

Data analysis was performed using Prism software (version 9; GraphPad Software, CA, USA). Statistical significance was determined by one-way ANOVA followed with Dunnett's or Bonferroni's multiple comparisons test. A p value <0.05 was considered statistically significant.

5 ^{1}H and ^{13}C NMR spectra

Compound **14**: ^{1}H, 400 MHz, DMSO-d_6

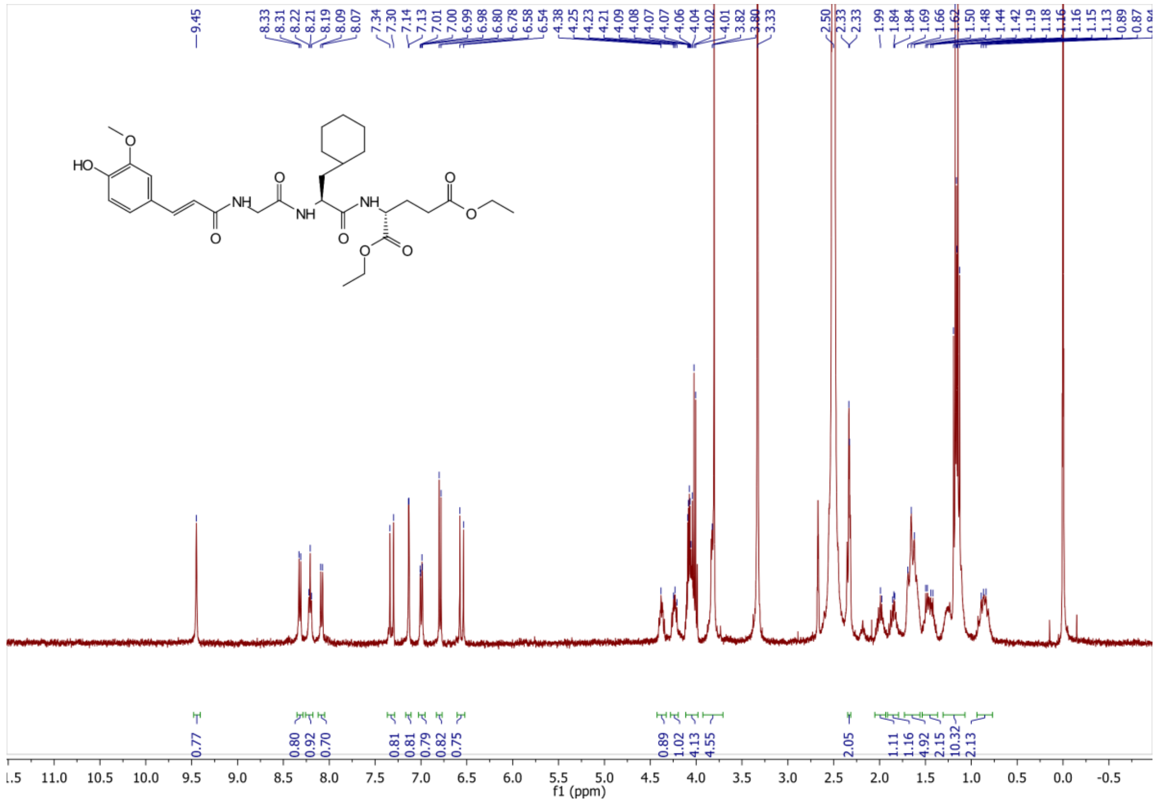

Compound **14**: ^{13}C, 101 MHz, DMSO-d_6

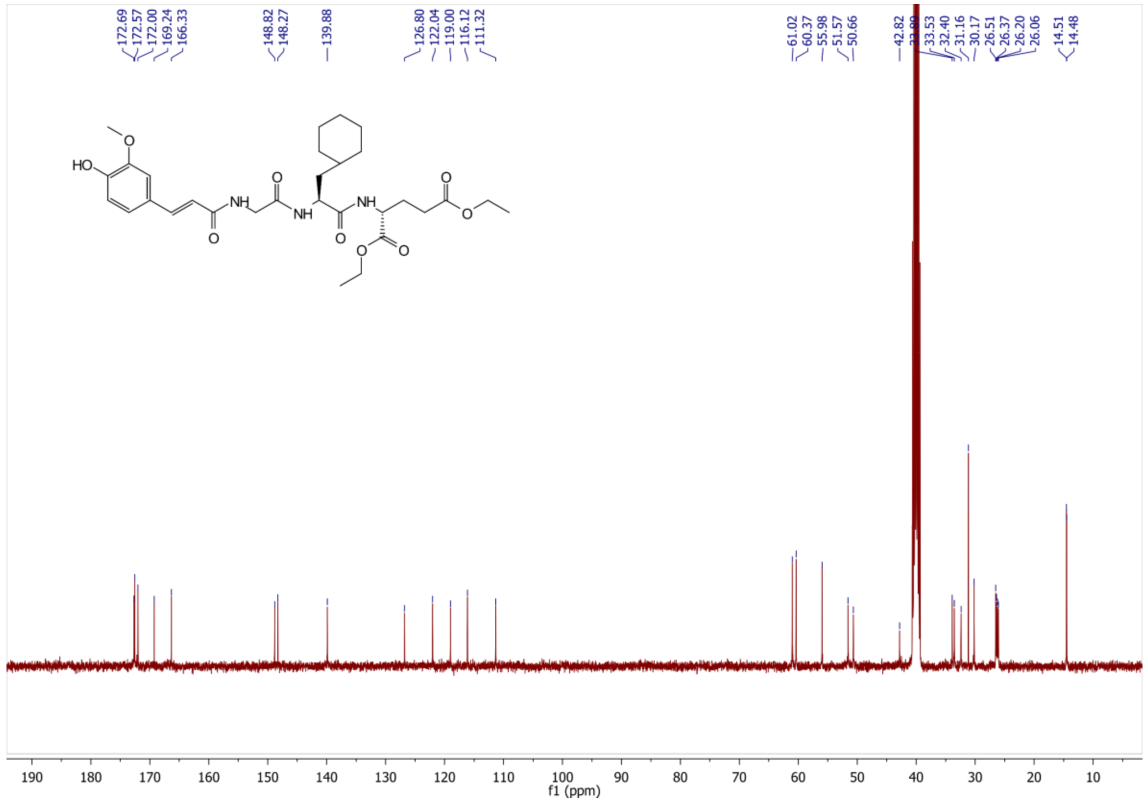

Compound **15**: ^{1}H, 400 MHz, DMSO-d_6

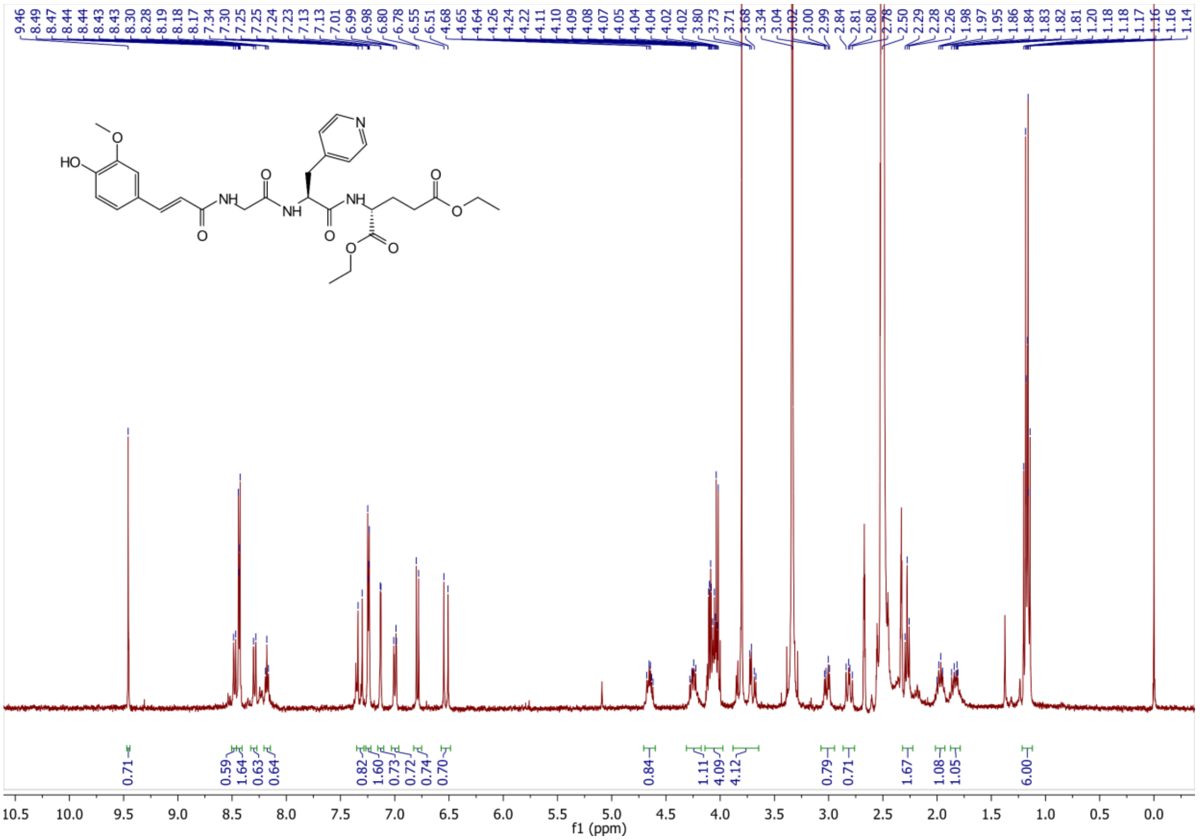

Compound **15**: ^{13}C, 101 MHz, DMSO-d_6

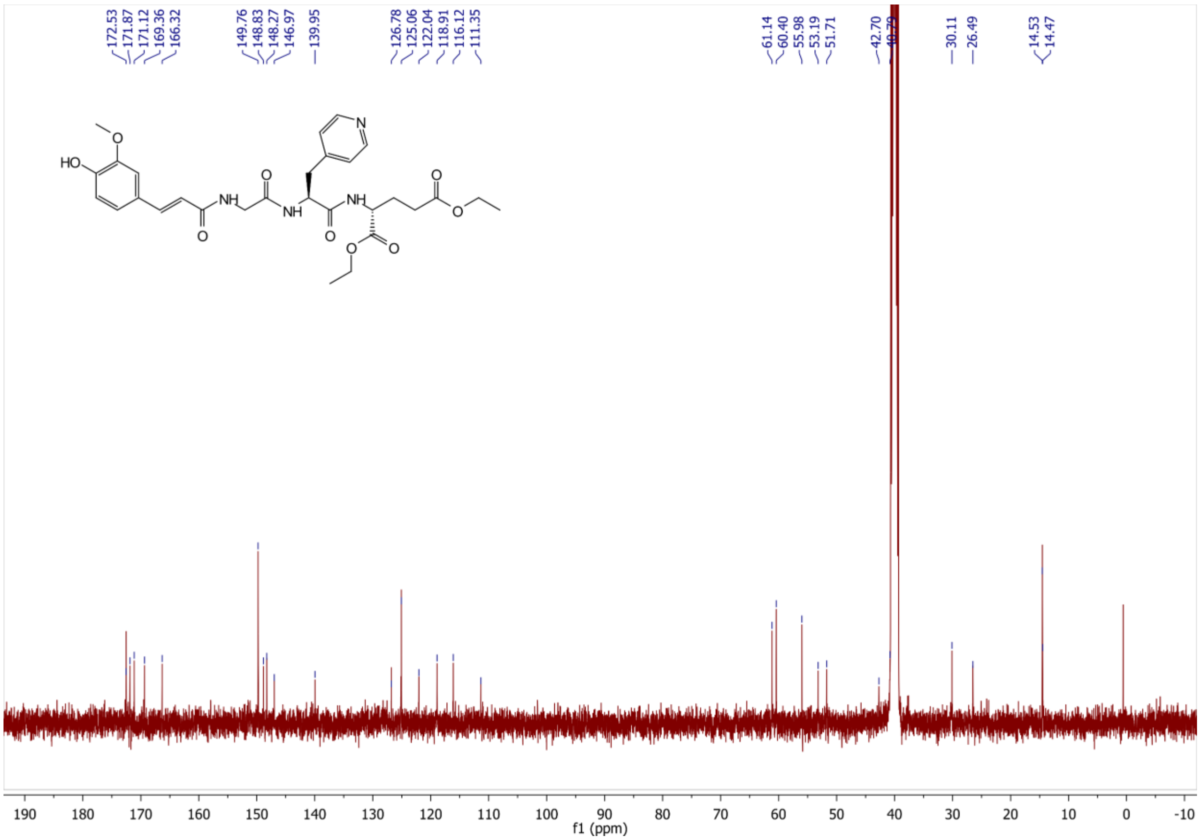

Compound **16**: ^{1}H, 400 MHz, DMSO-d_6

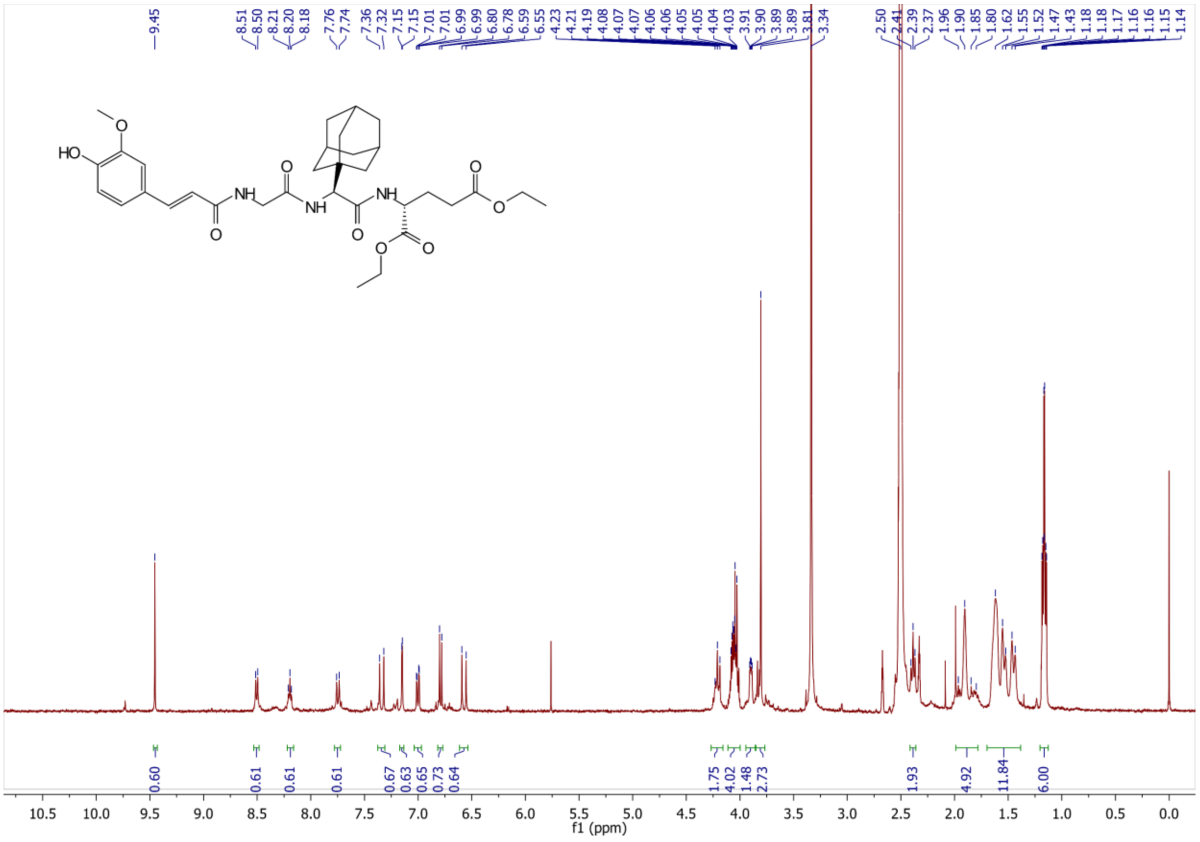

Compound **16**: ^{13}C, 101 MHz, DMSO-d_6

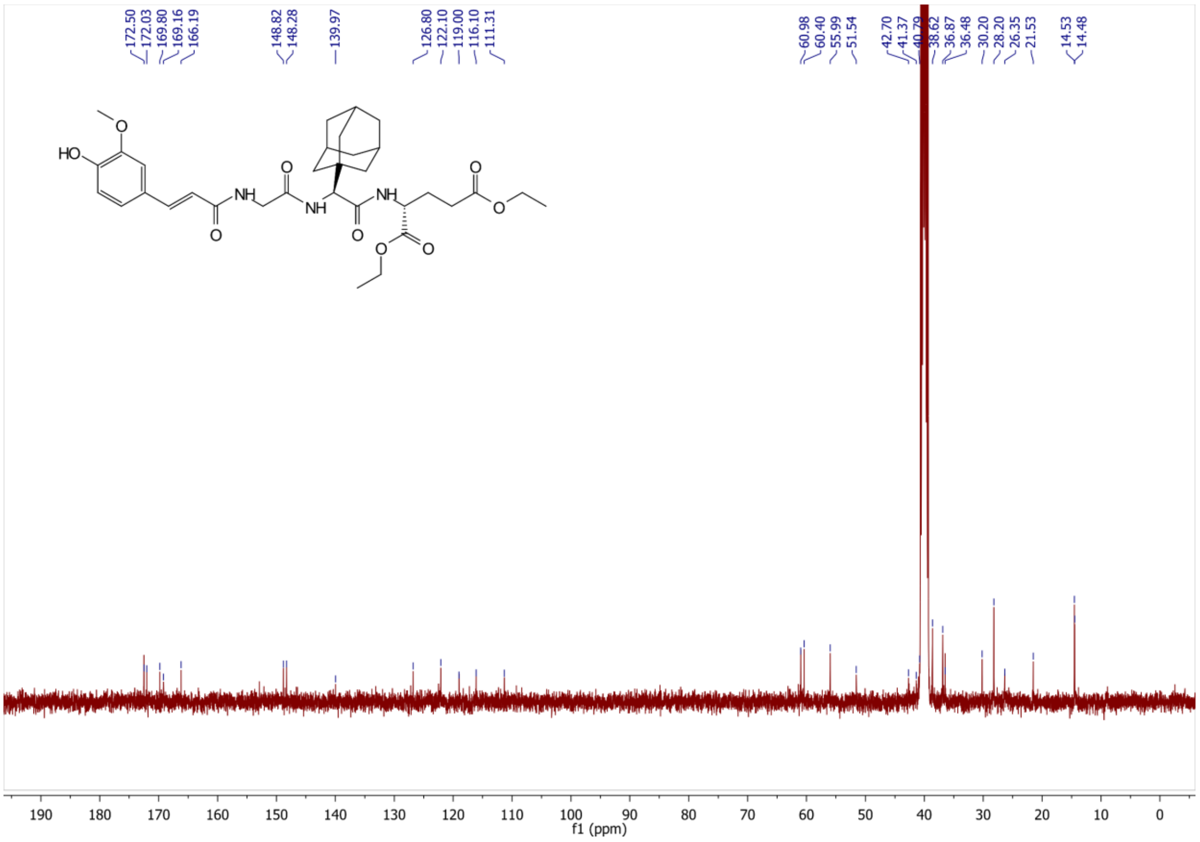

Compound **17**: ^{1}H, 400 MHz, DMSO-d_6

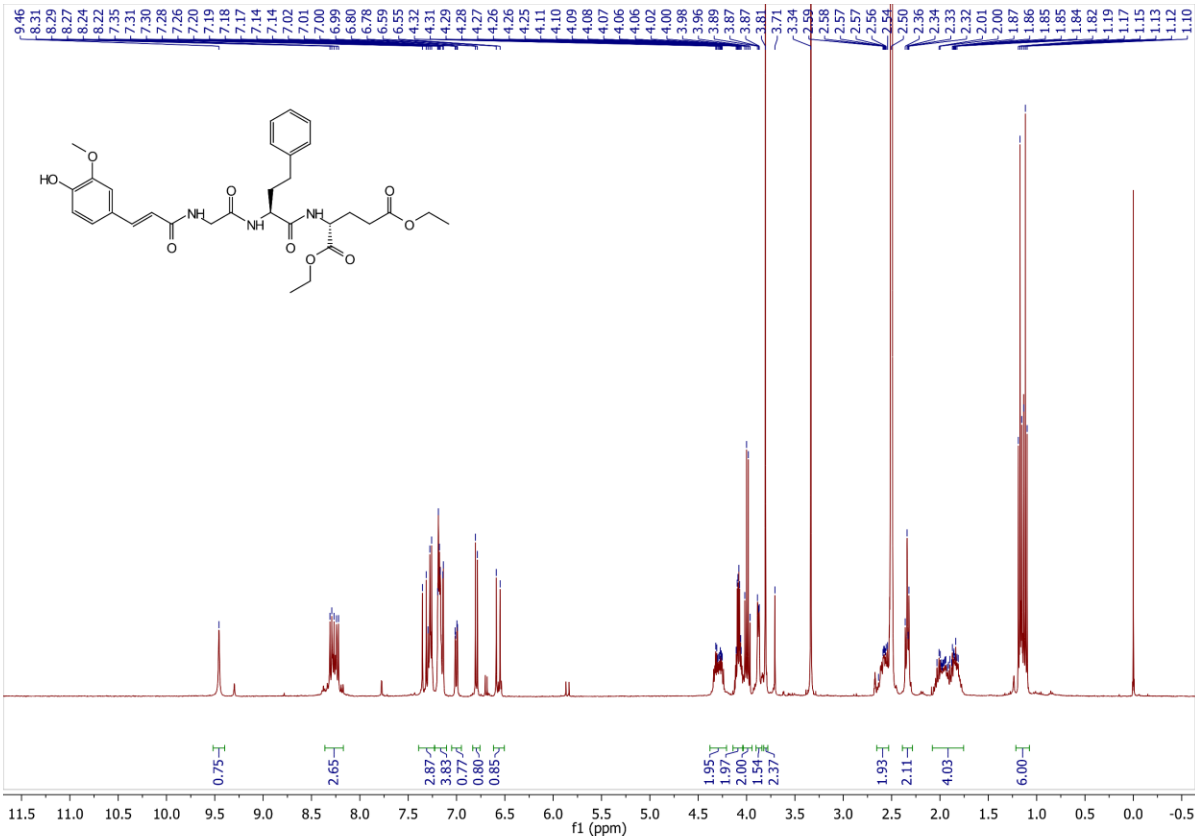

Compound **17**: ^{13}C, 101 MHz, DMSO-d_6

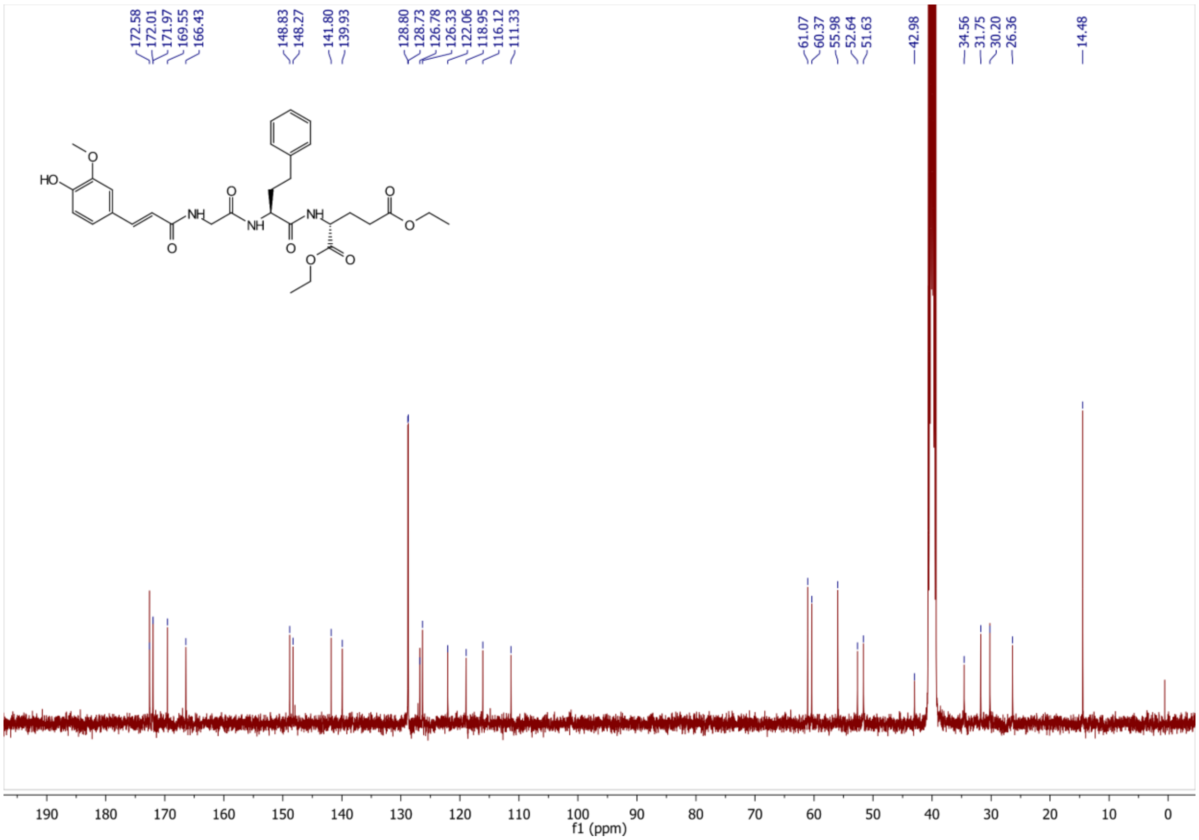

Compound **18**: ^{1}H, 400 MHz, DMSO-d_6

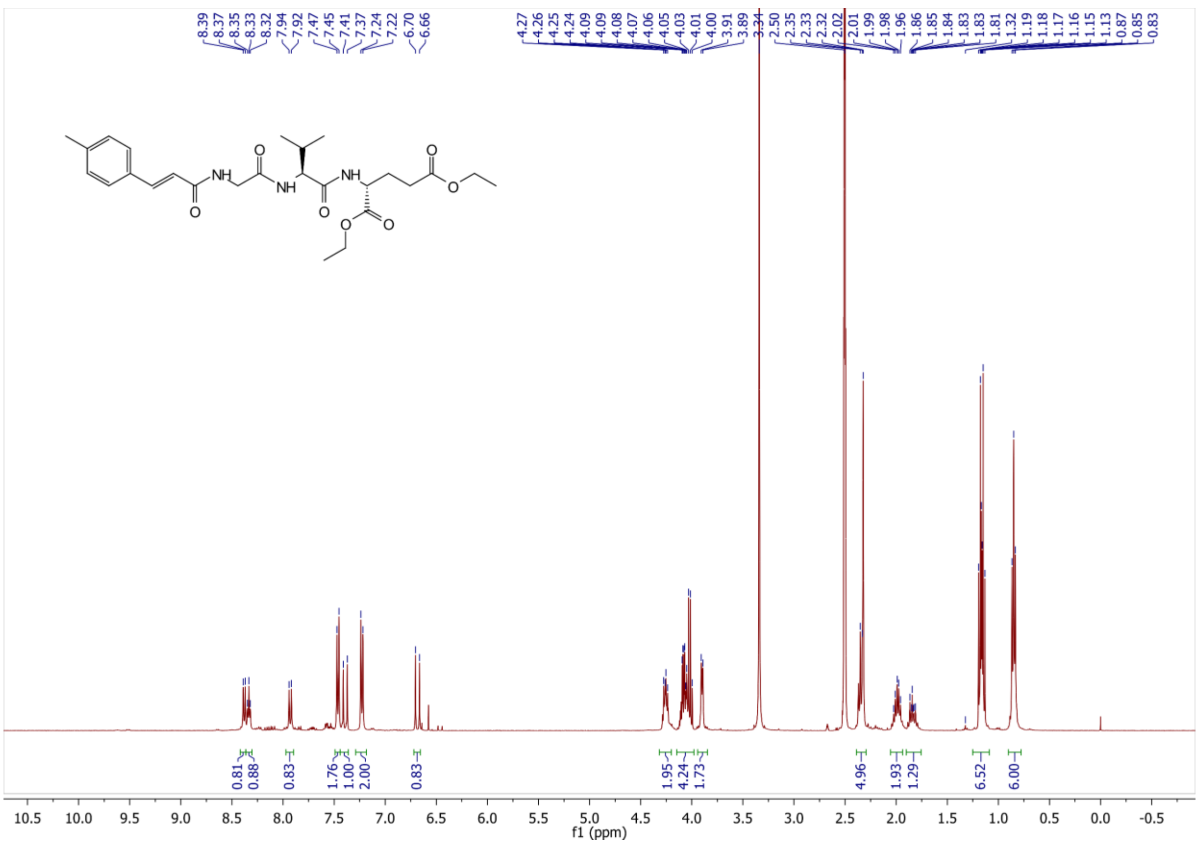

Compound **18**: ^{13}C, 101 MHz, DMSO-d_6

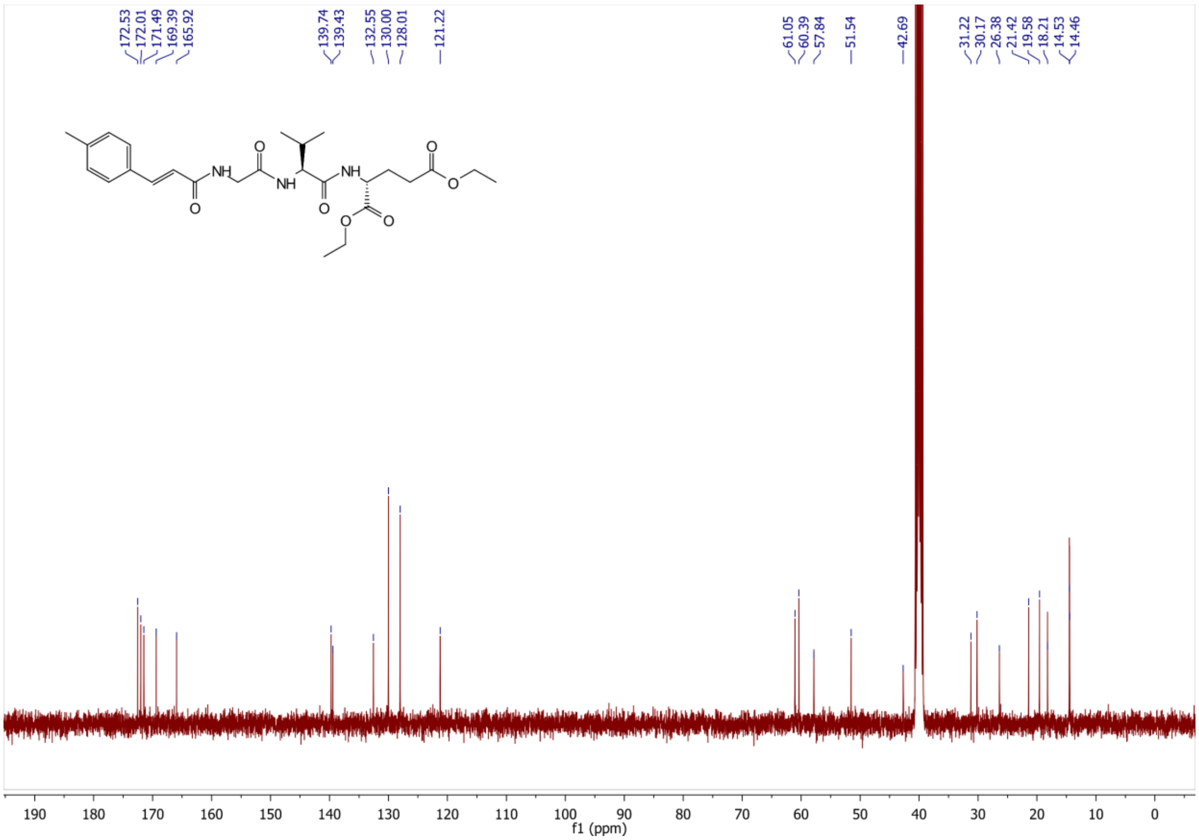

Compound **19**: ^{1}H, 400 MHz, DMSO-d_6

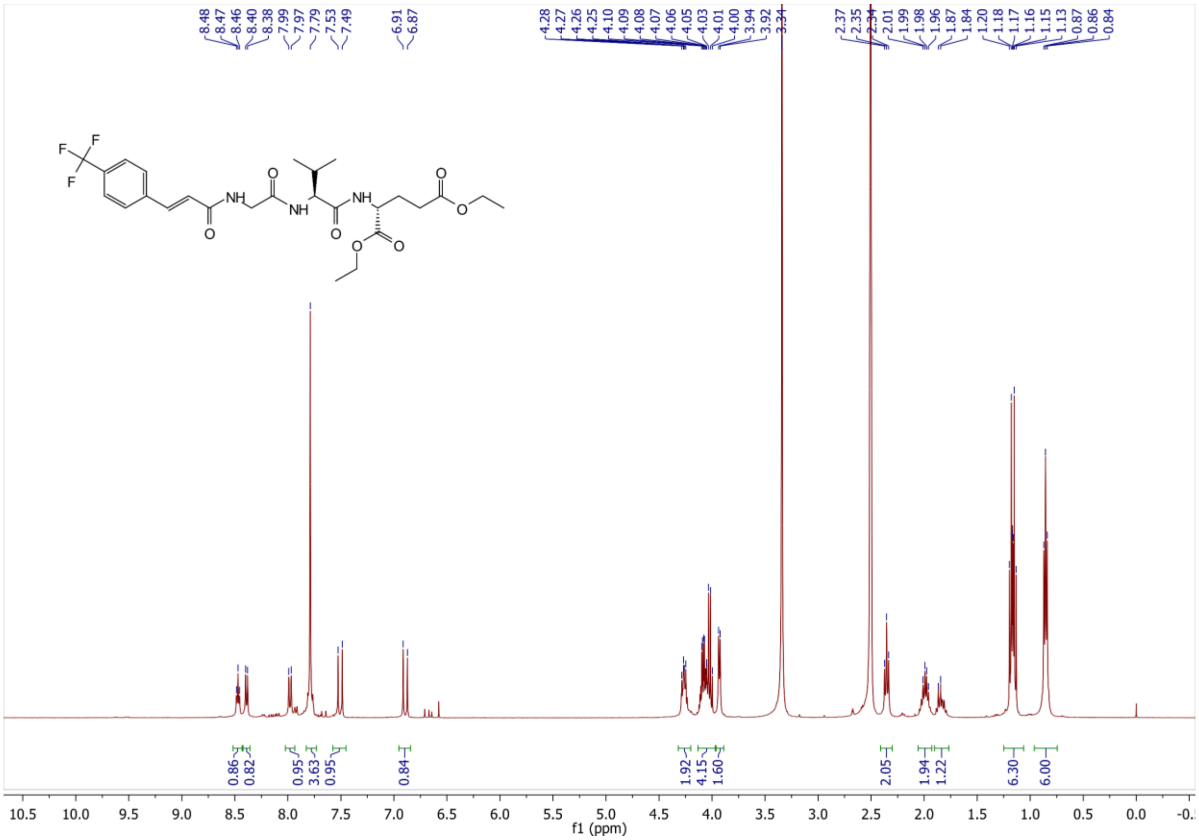

Compound **19**: ^{13}C, 101 MHz, DMSO-d_6

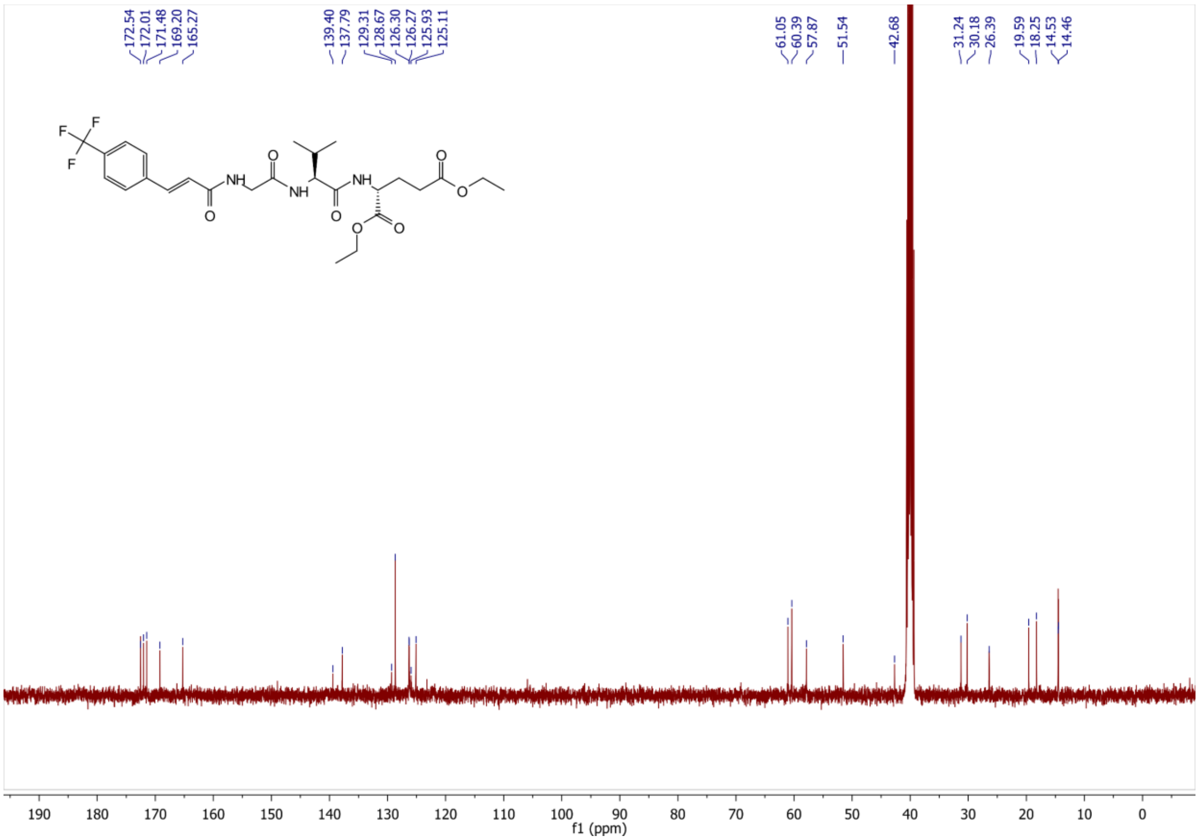

Compound **20**: ^{1}H, 400 MHz, DMSO-d_6

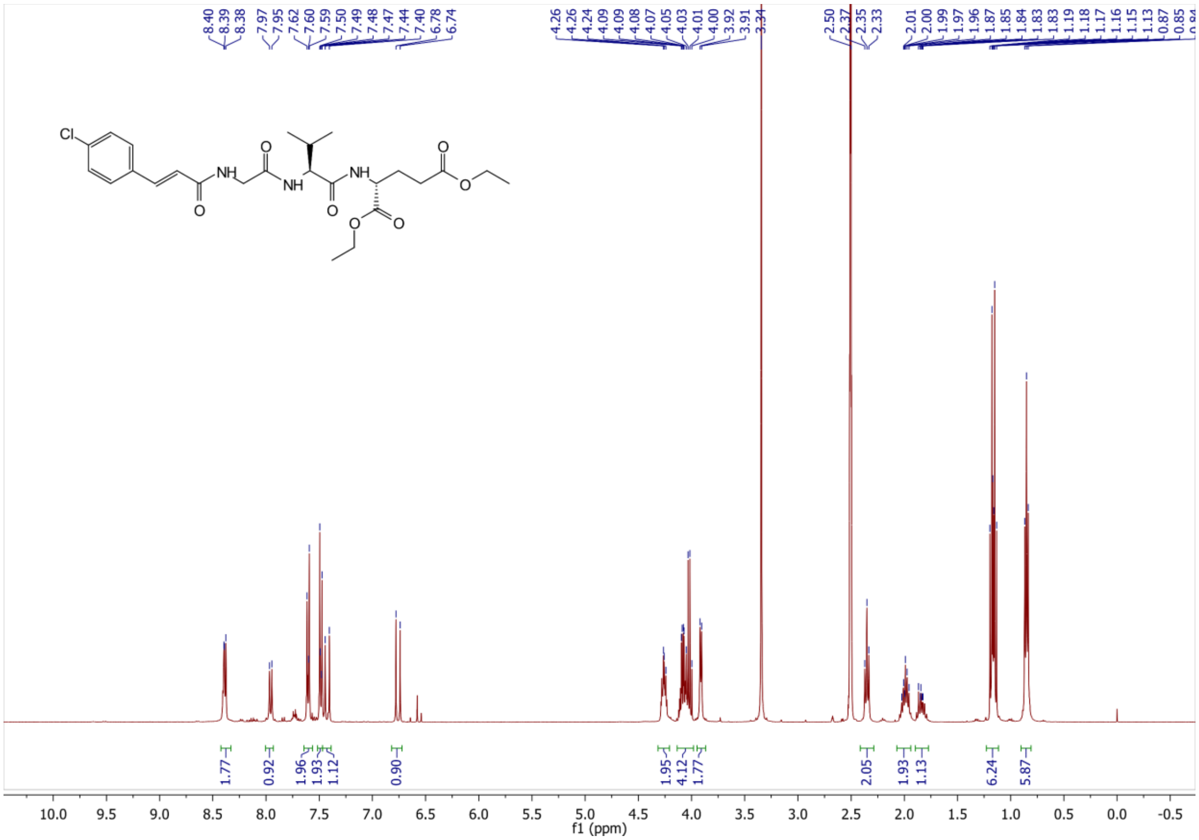

Compound **20**: ^{13}C, 101 MHz, DMSO-d_6

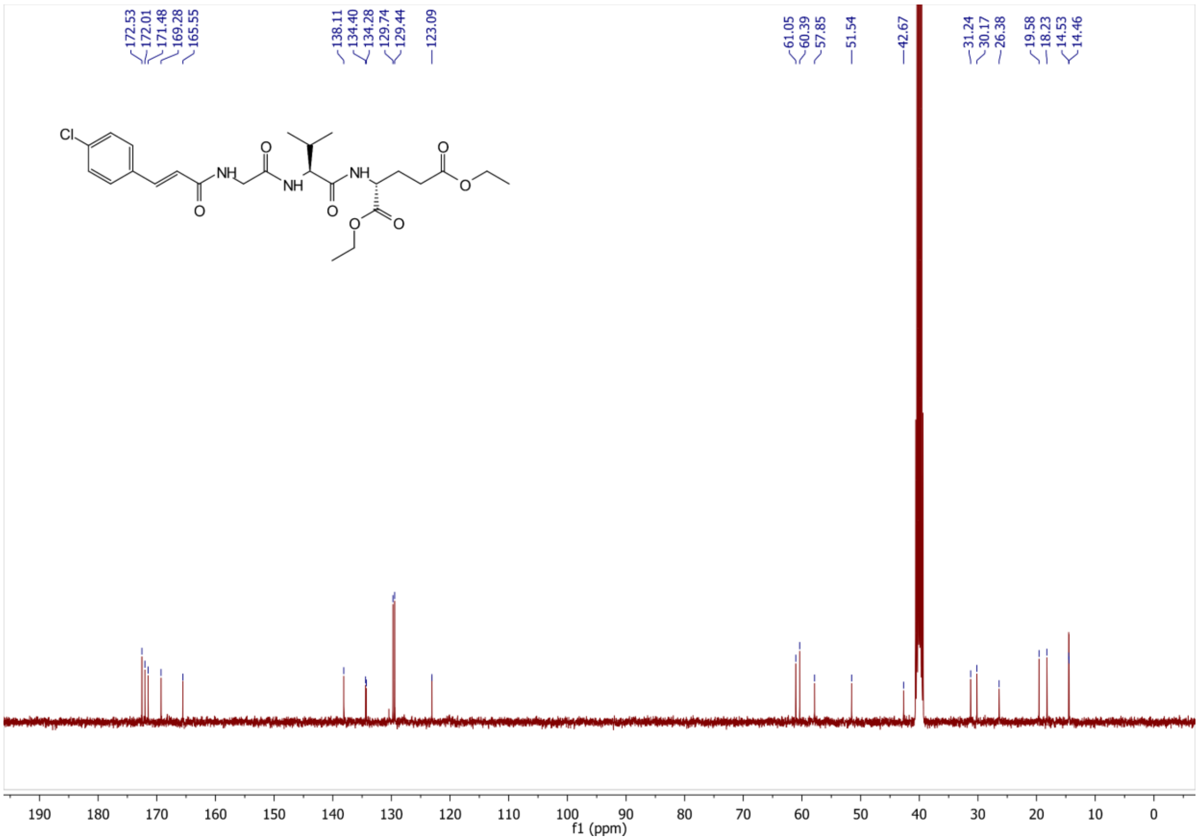

Compound **21**: ^{1}H, 400 MHz, DMSO-d_6

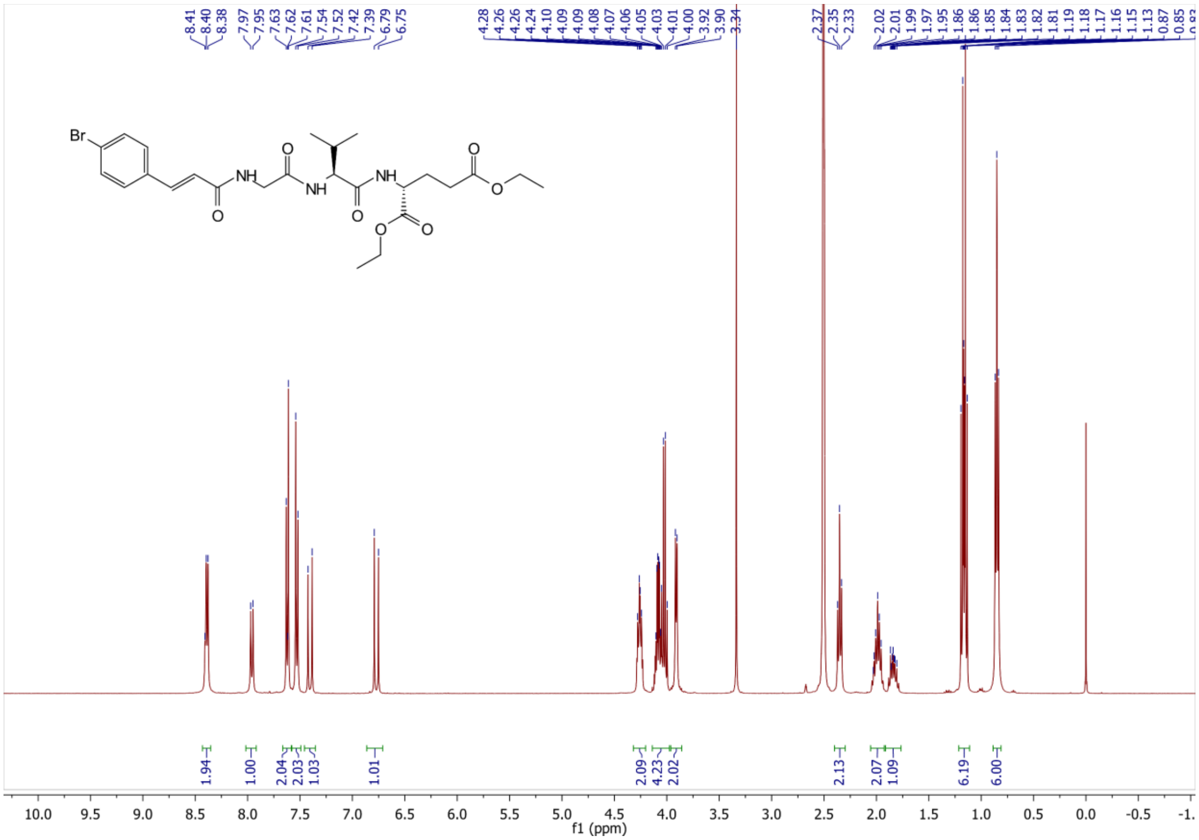

Compound **21**: ^{13}C, 101 MHz, DMSO-d_6

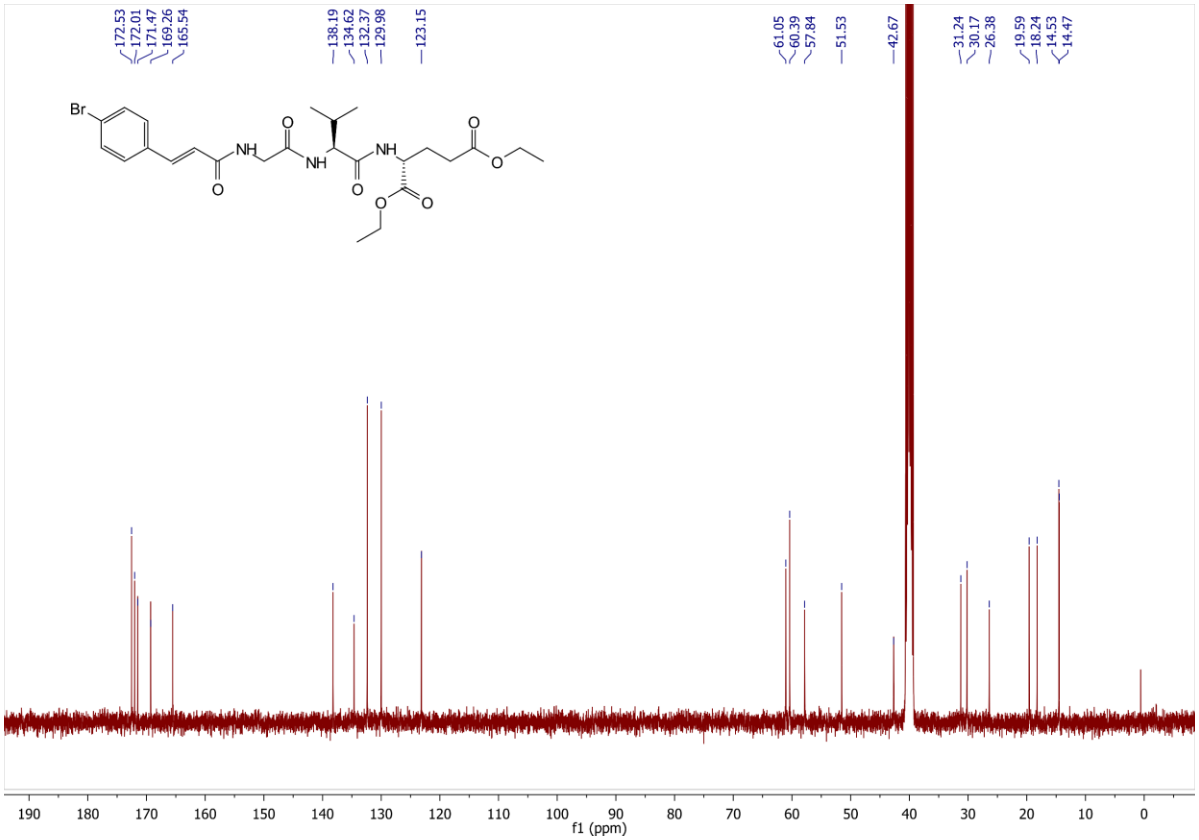

Compound **22**: ^{1}H, 400 MHz, DMSO-d_6

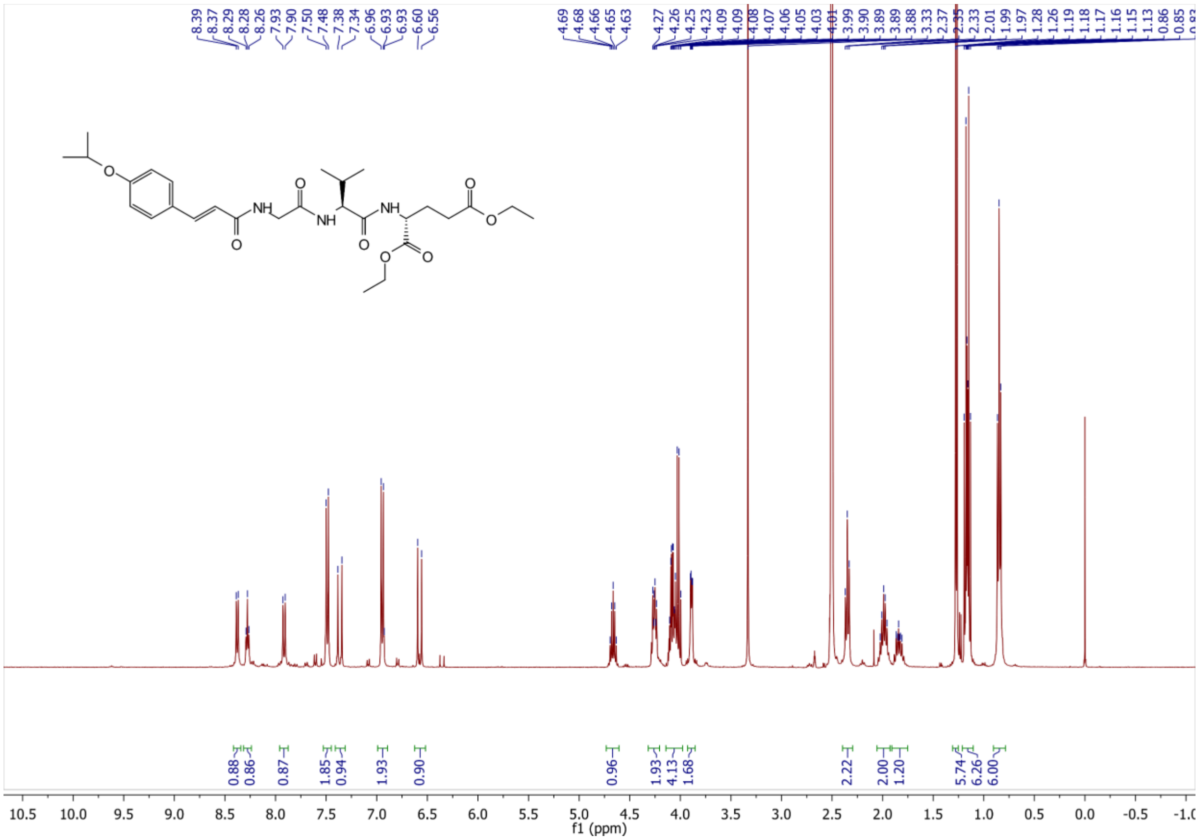

Compound **22**: ^{13}C, 101 MHz, DMSO-d_6

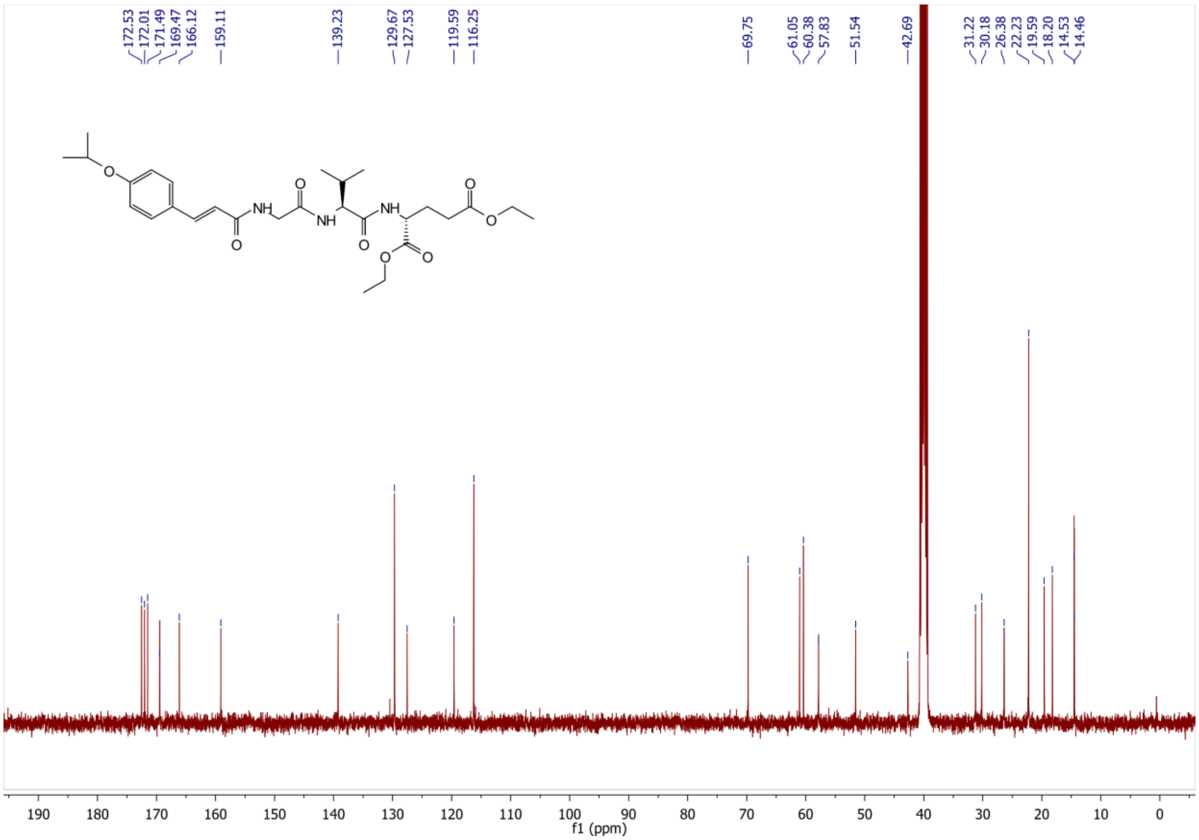

Compound **23**: ^{1}H, 400 MHz, DMSO-d_6

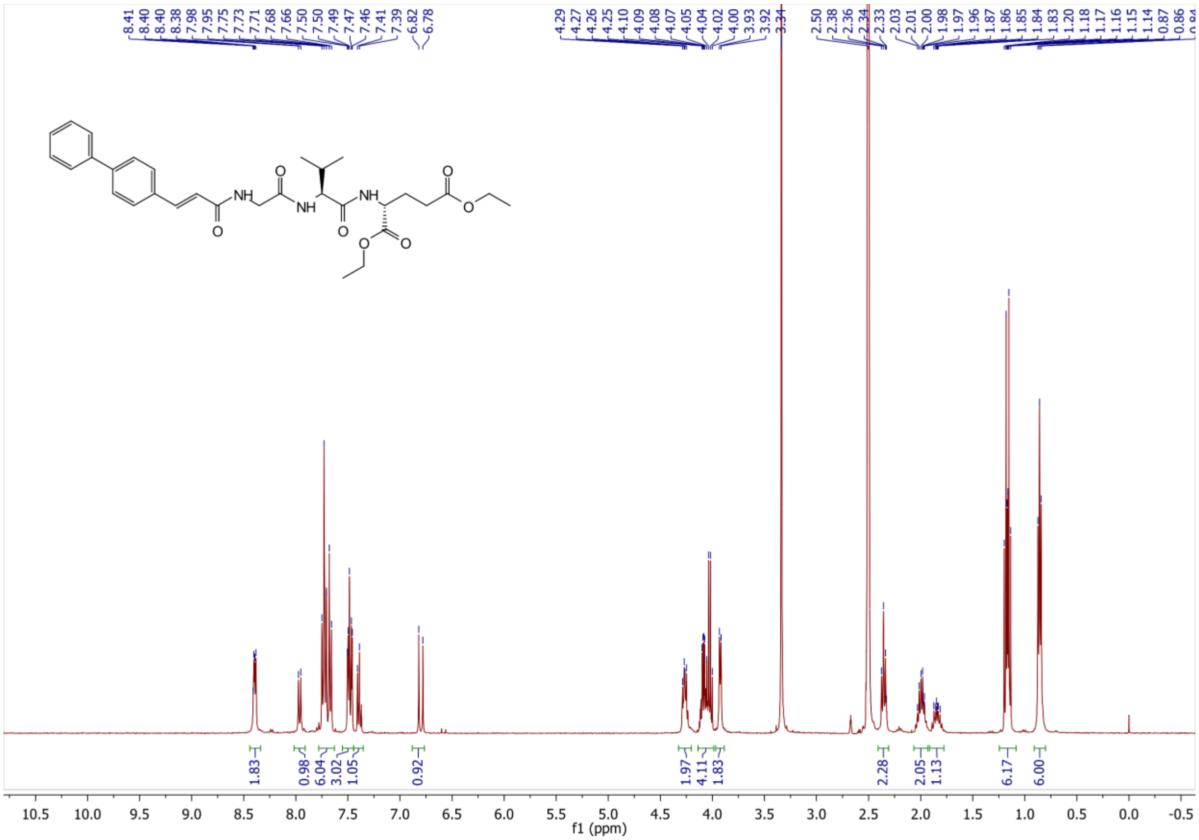

Compound **23**: ^{13}C, 101 MHz, DMSO-d_6

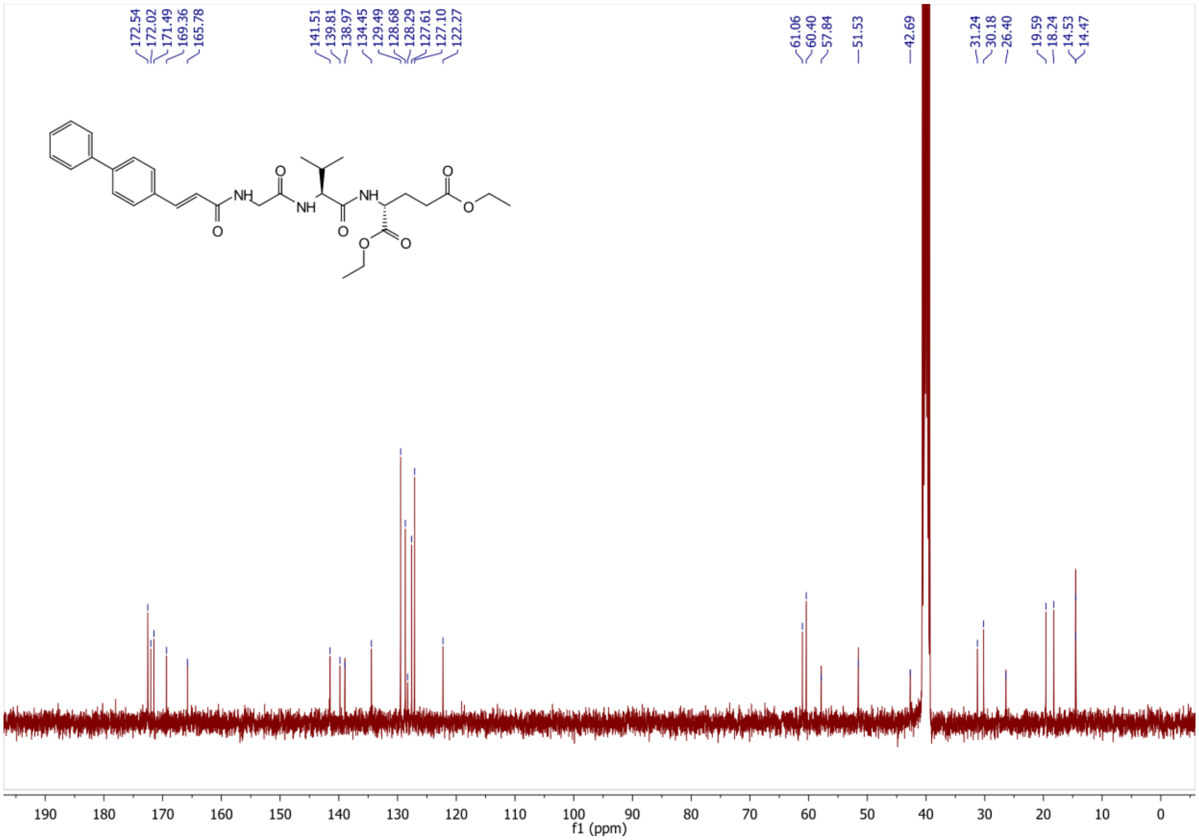

Compound **24**: ^{1}H, 400 MHz, DMSO-d_6

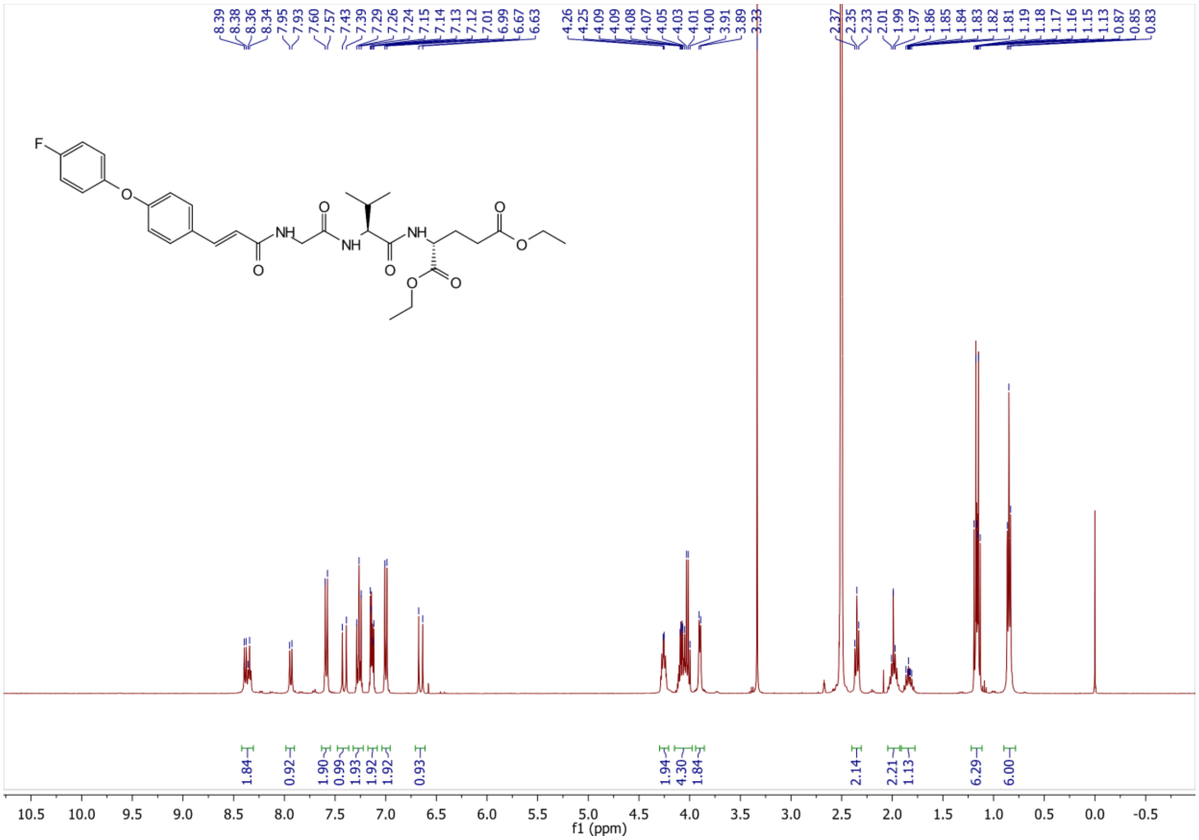

Compound **24**: ^{13}C, 101 MHz, DMSO-d_6

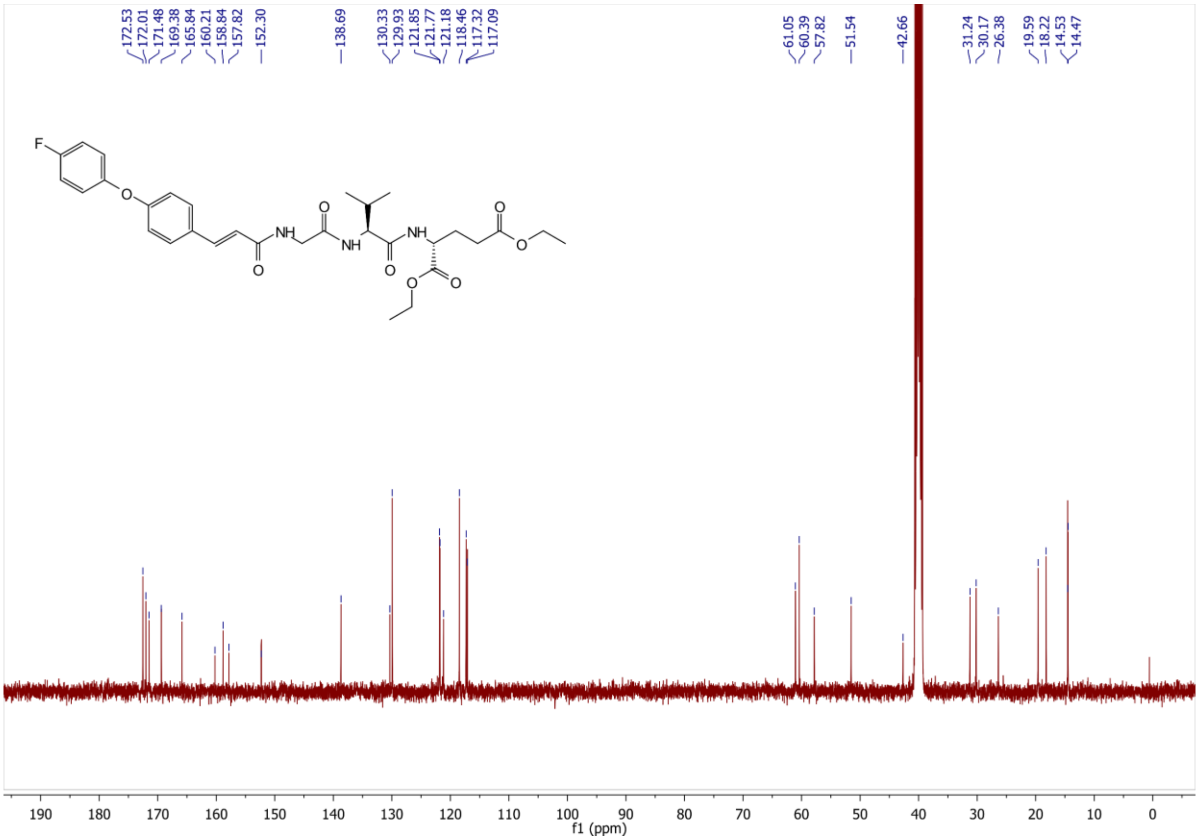

Compound **25**: ^{1}H, 400 MHz, DMSO-d_6

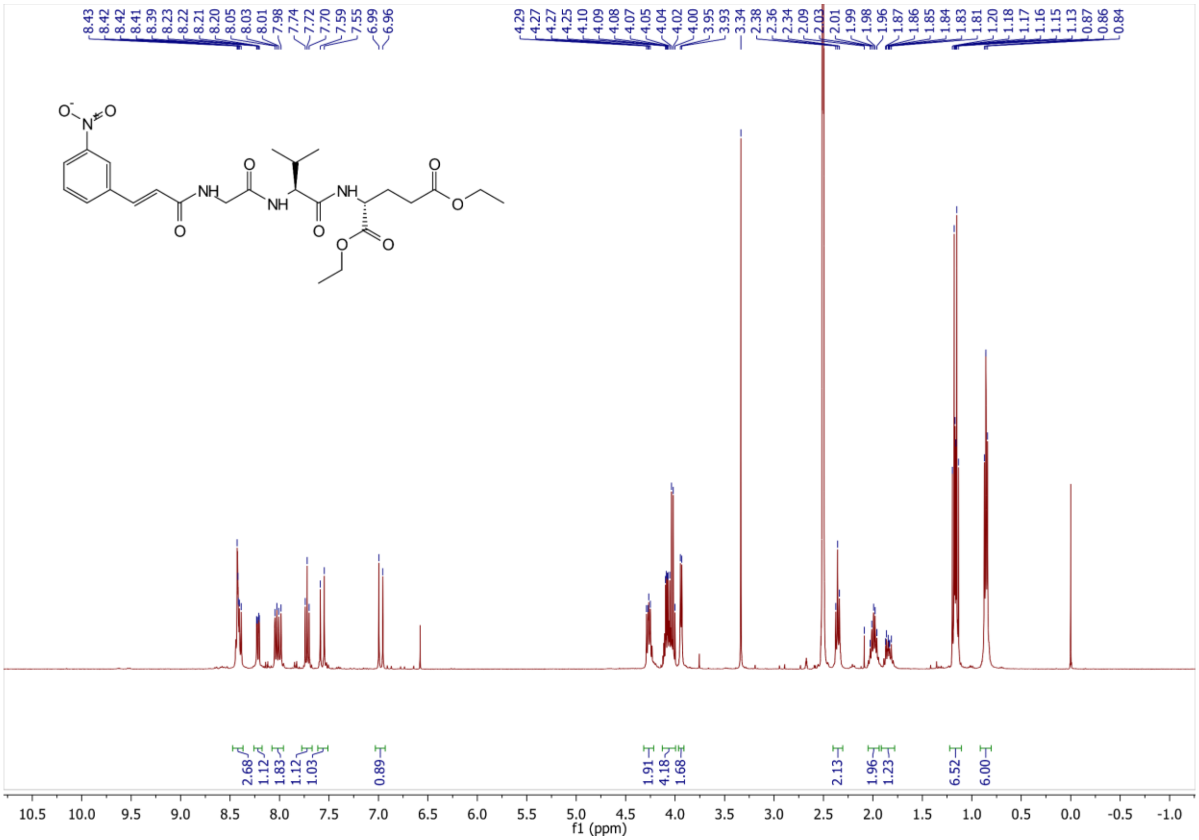

Compound **25**: ^{13}C, 101 MHz, DMSO-d_6

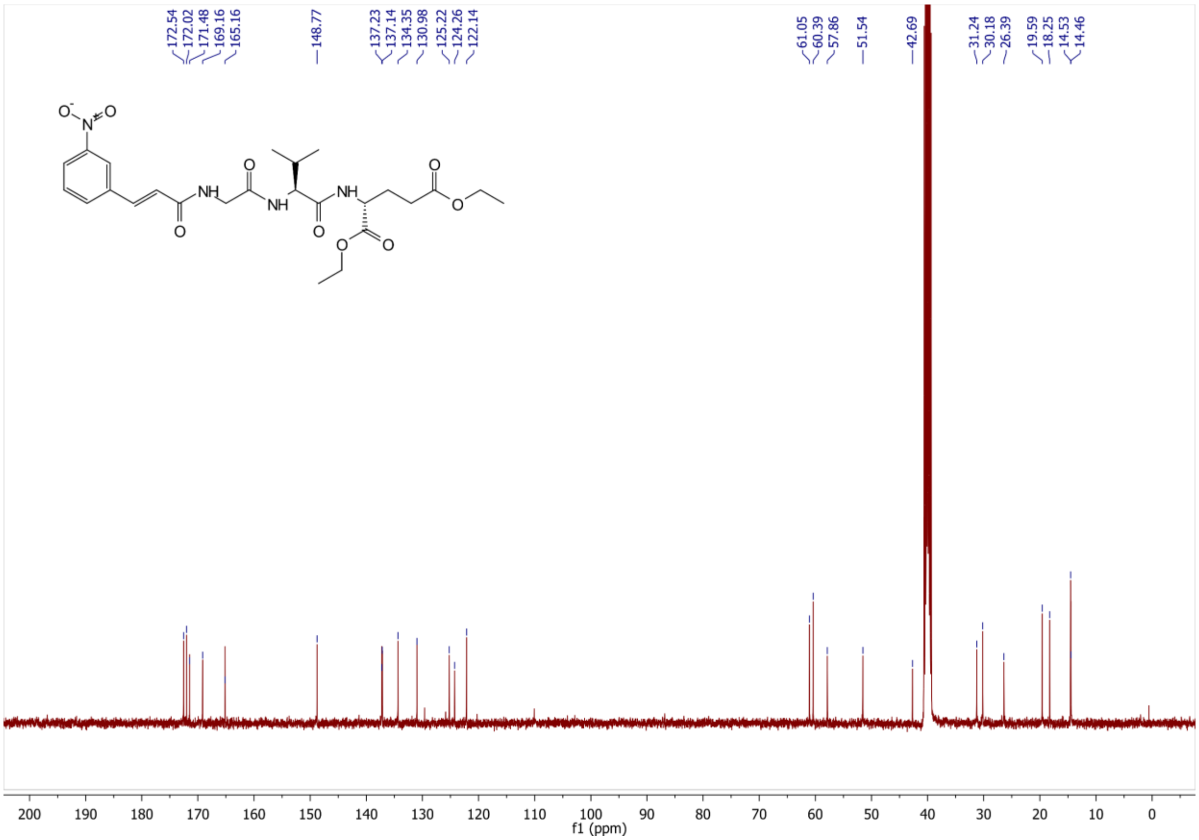

Compound **26**: ^{1}H, 400 MHz, DMSO-d_6

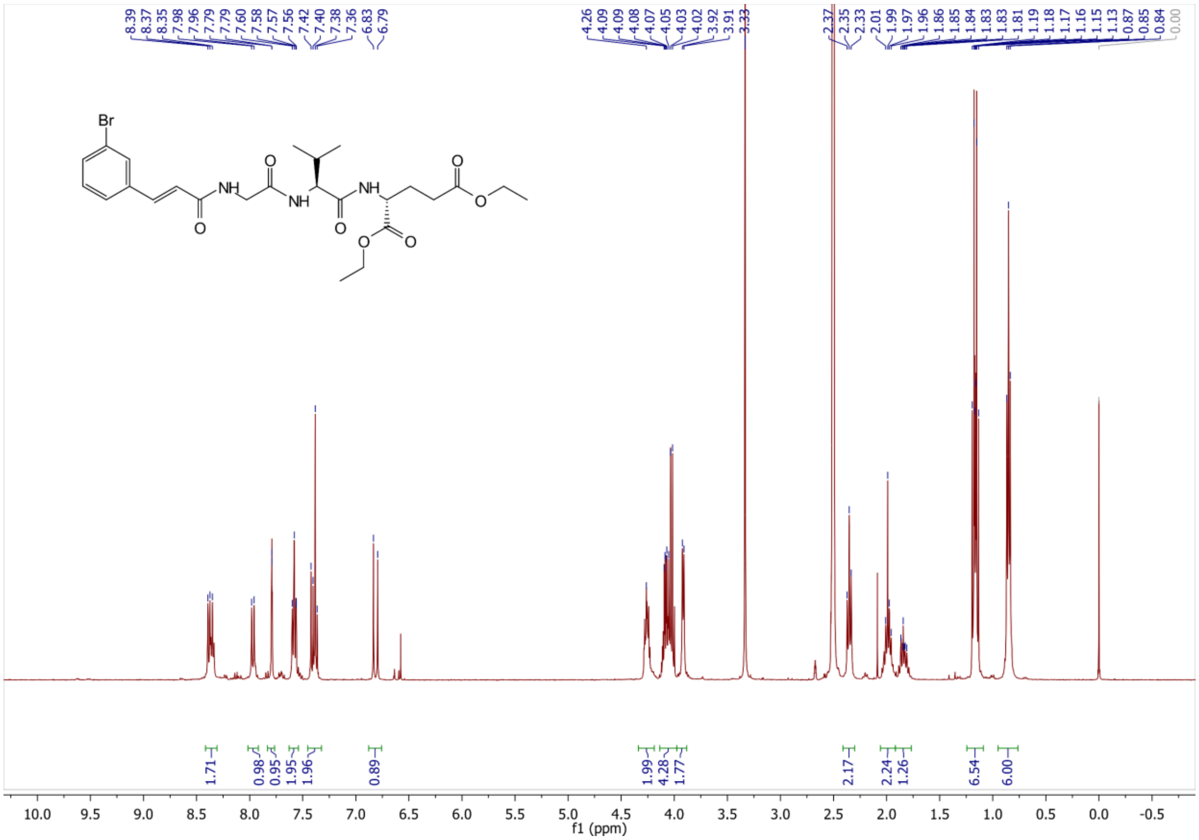

Compound **26**: ^{13}C, 101 MHz, DMSO-d_6

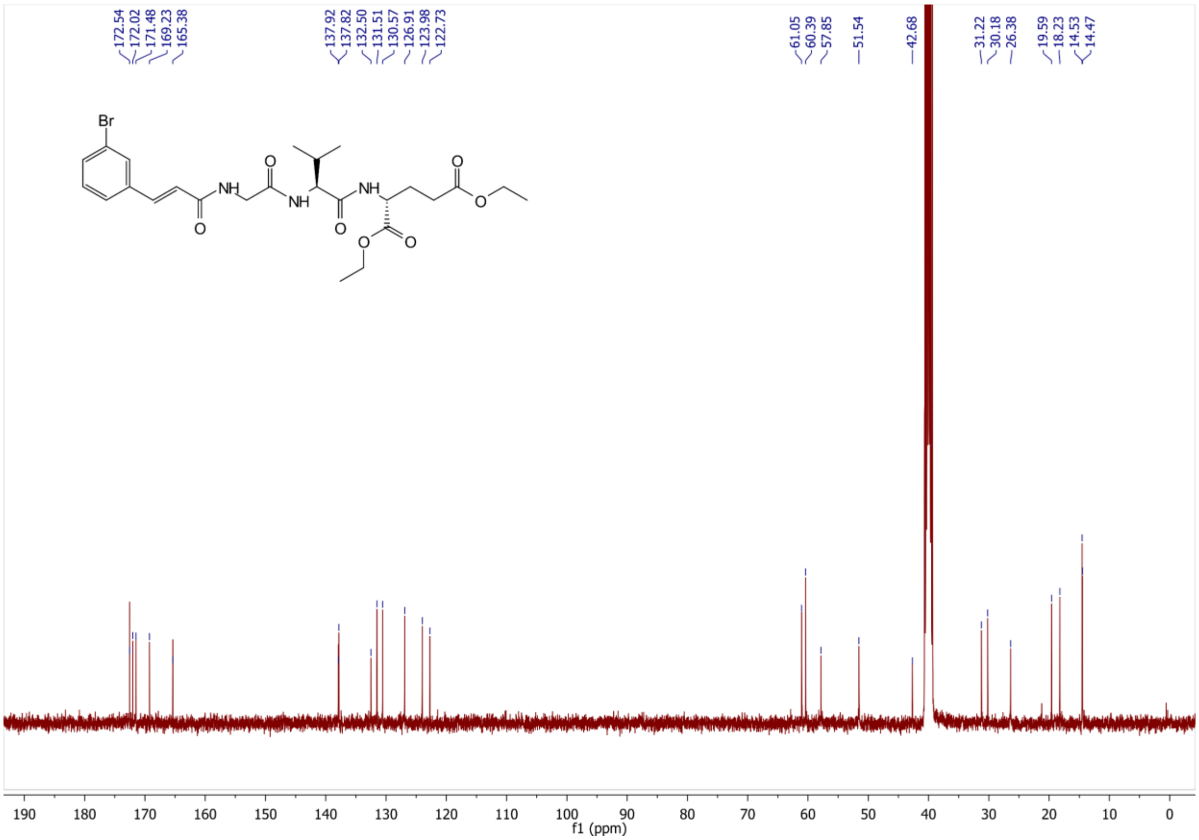

Compound **27**: ^{1}H, 400 MHz, DMSO-d_6

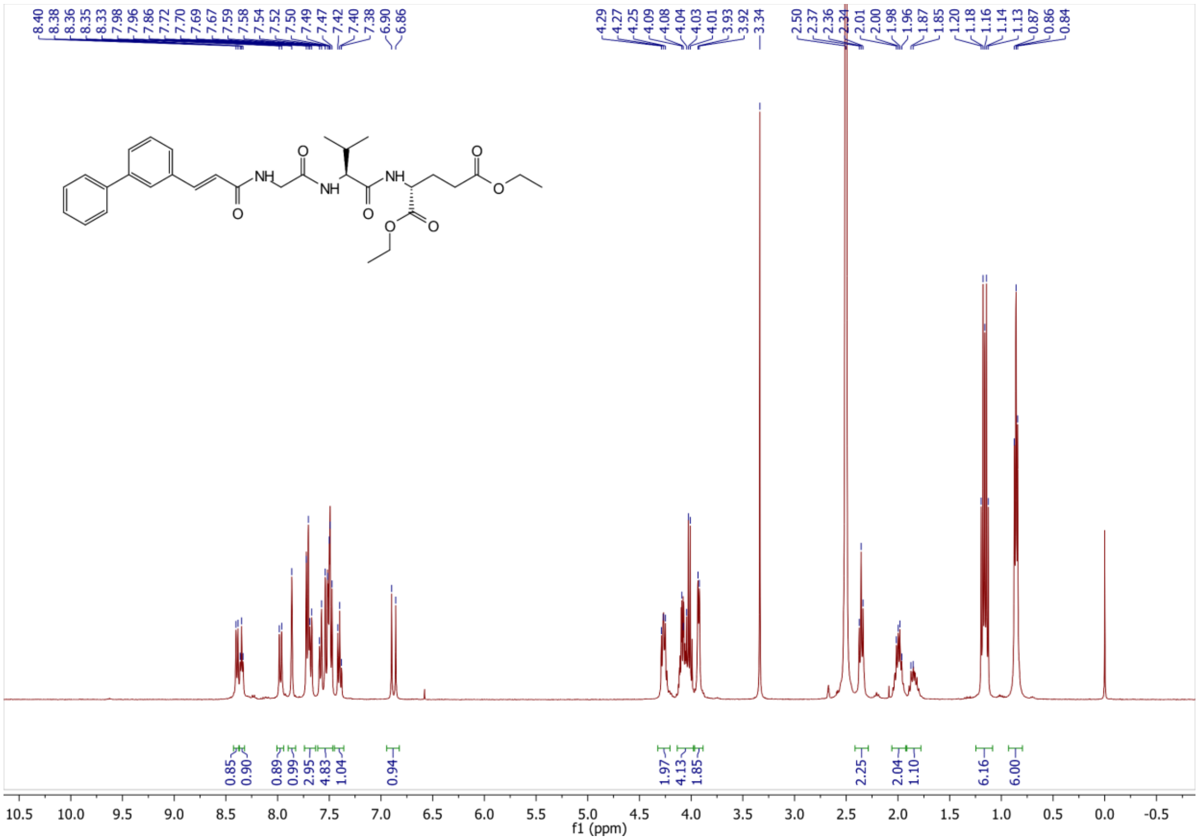

Compound **27**: ^{13}C, 101 MHz, DMSO-d_6

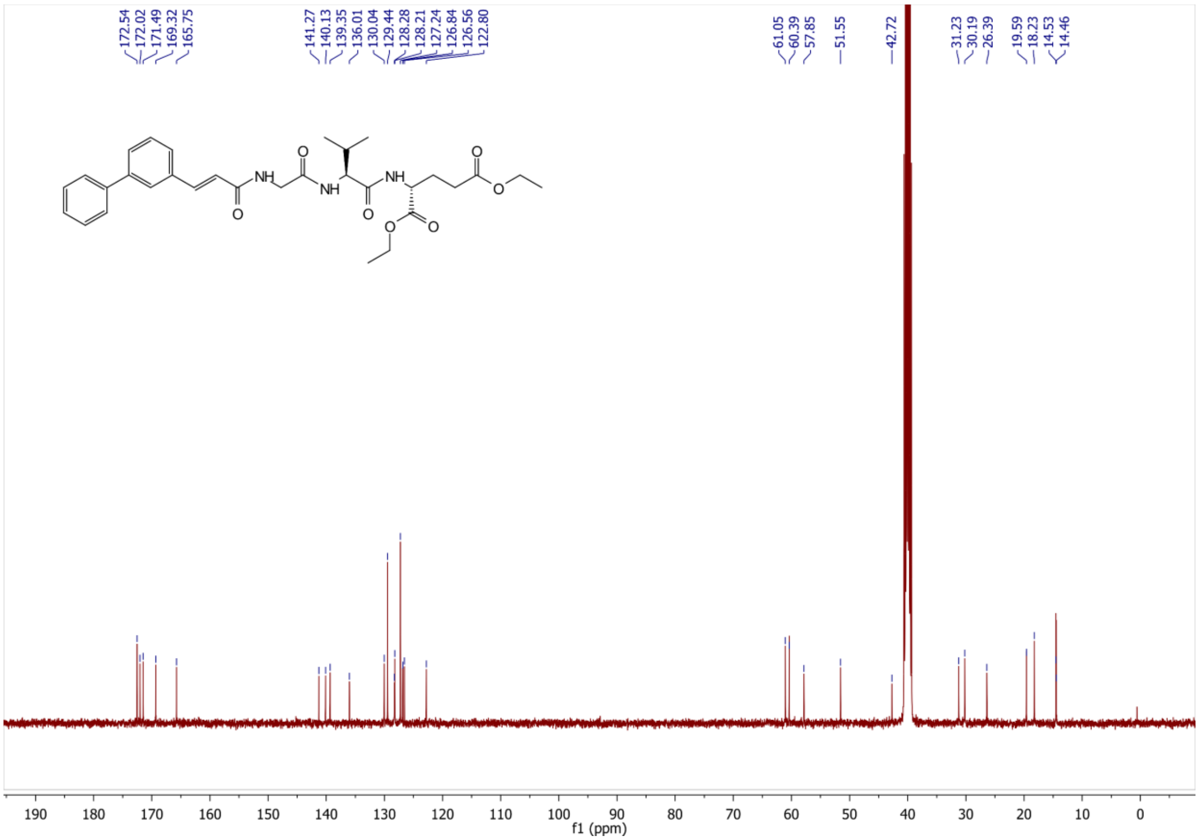

Compound **28**: ^{1}H, 400 MHz, DMSO-d_6

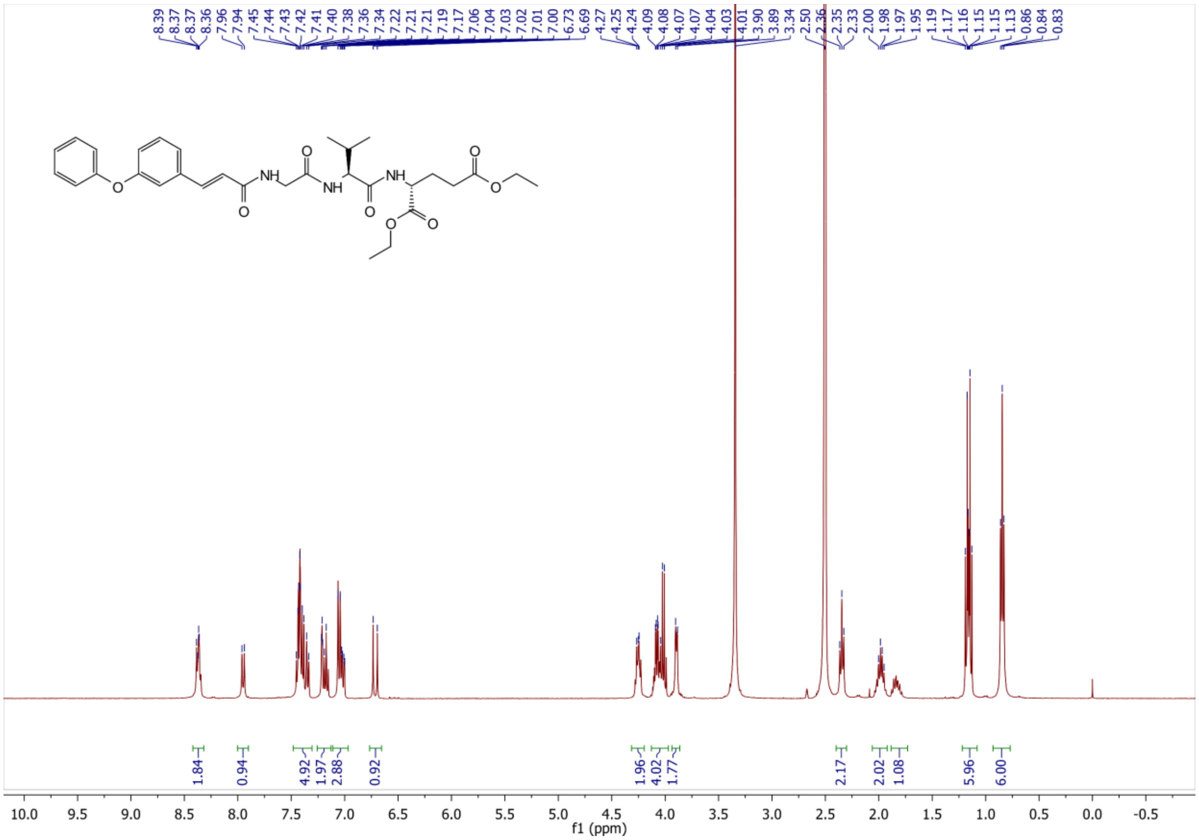

Compound **28**: ^{13}C, 101 MHz, DMSO-d_6

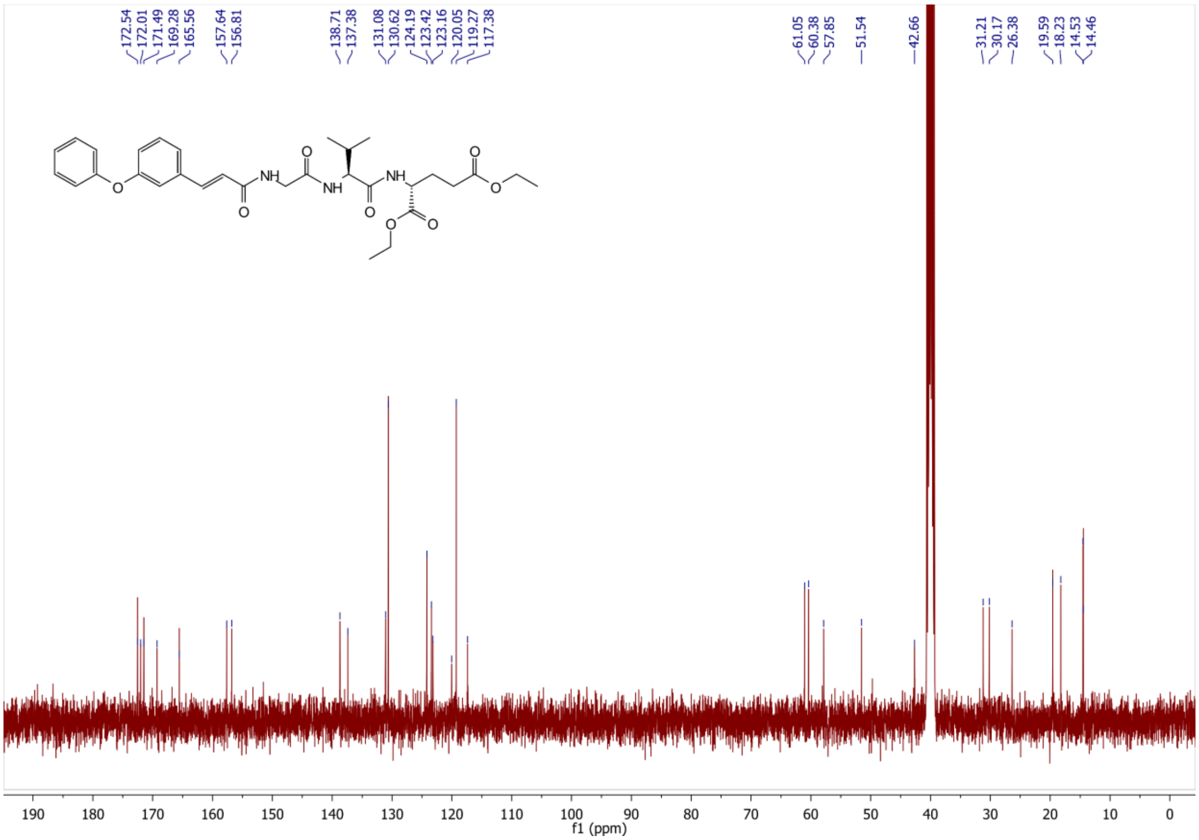

Compound **29**: ^{1}H, 400 MHz, DMSO-d_6

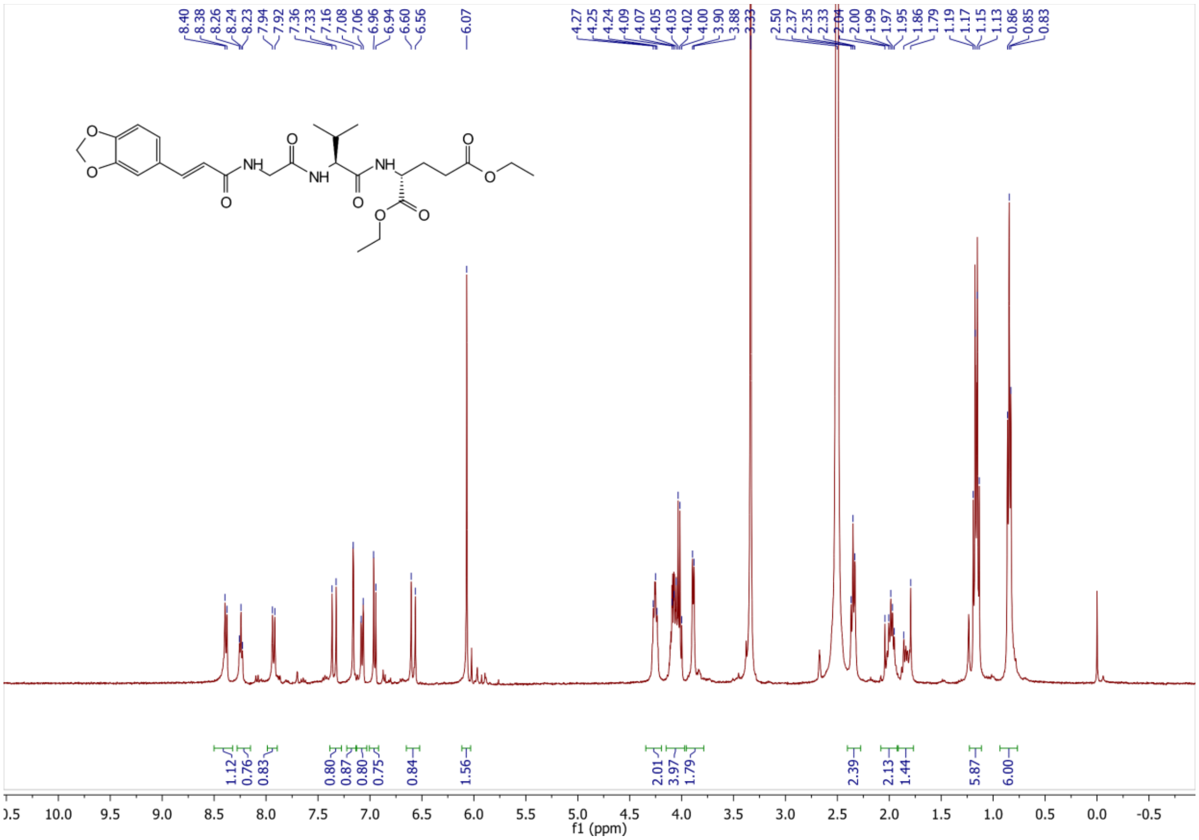

Compound **29**: ^{13}C, 101 MHz, DMSO-d_6

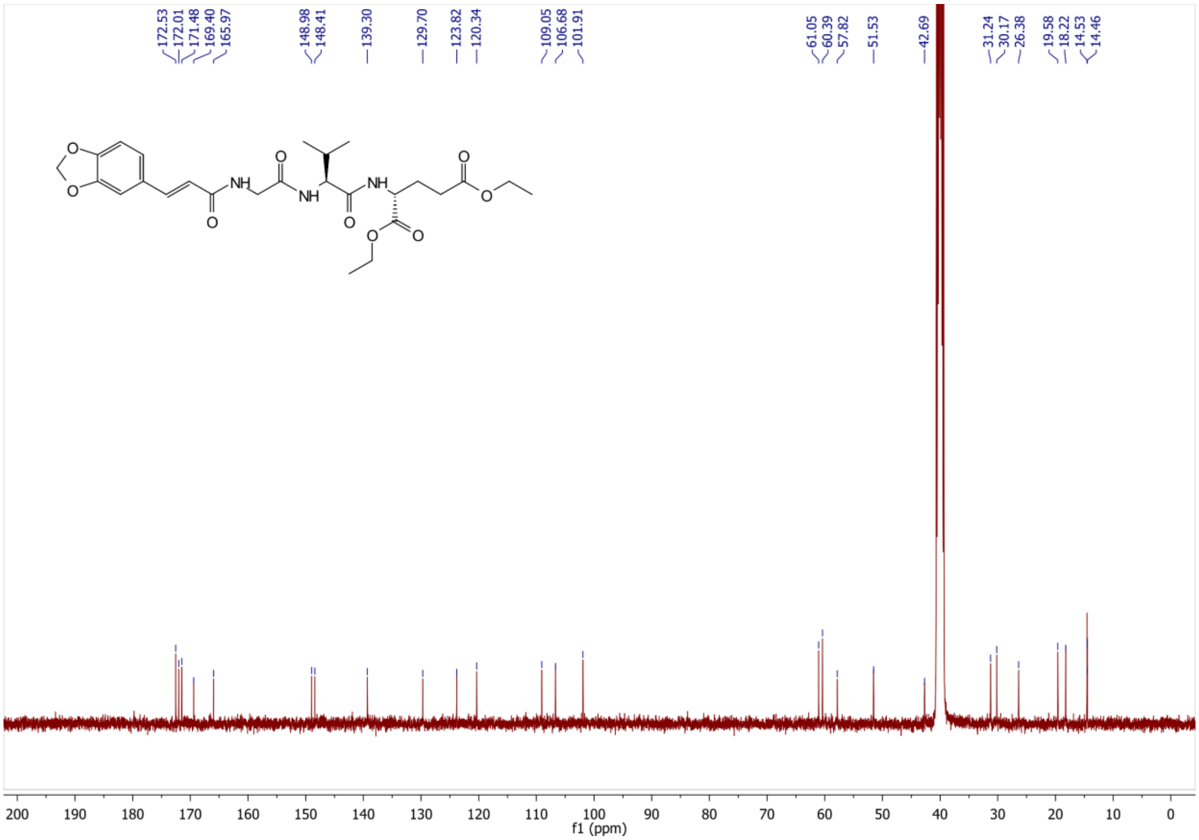

Compound **30**: ^{1}H, 400 MHz, DMSO-d_6

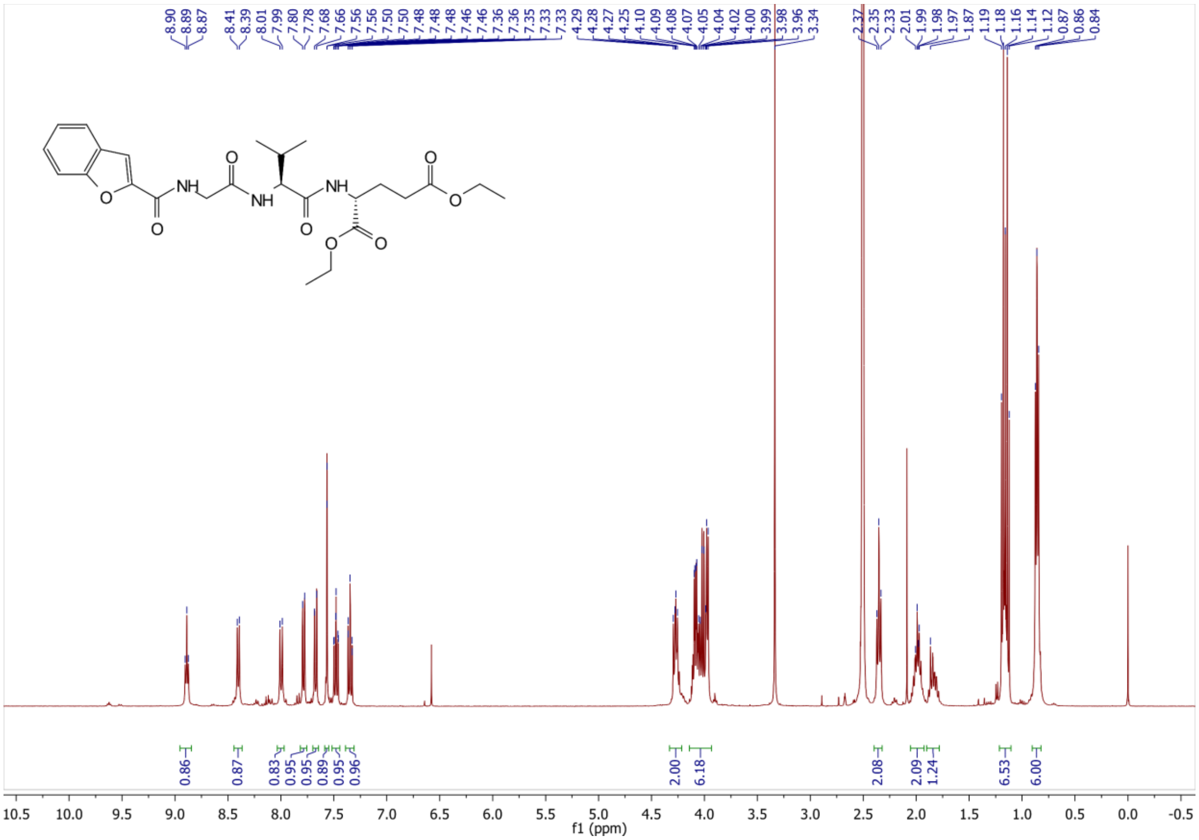

Compound **30**: ^{13}C, 101 MHz, DMSO-d_6

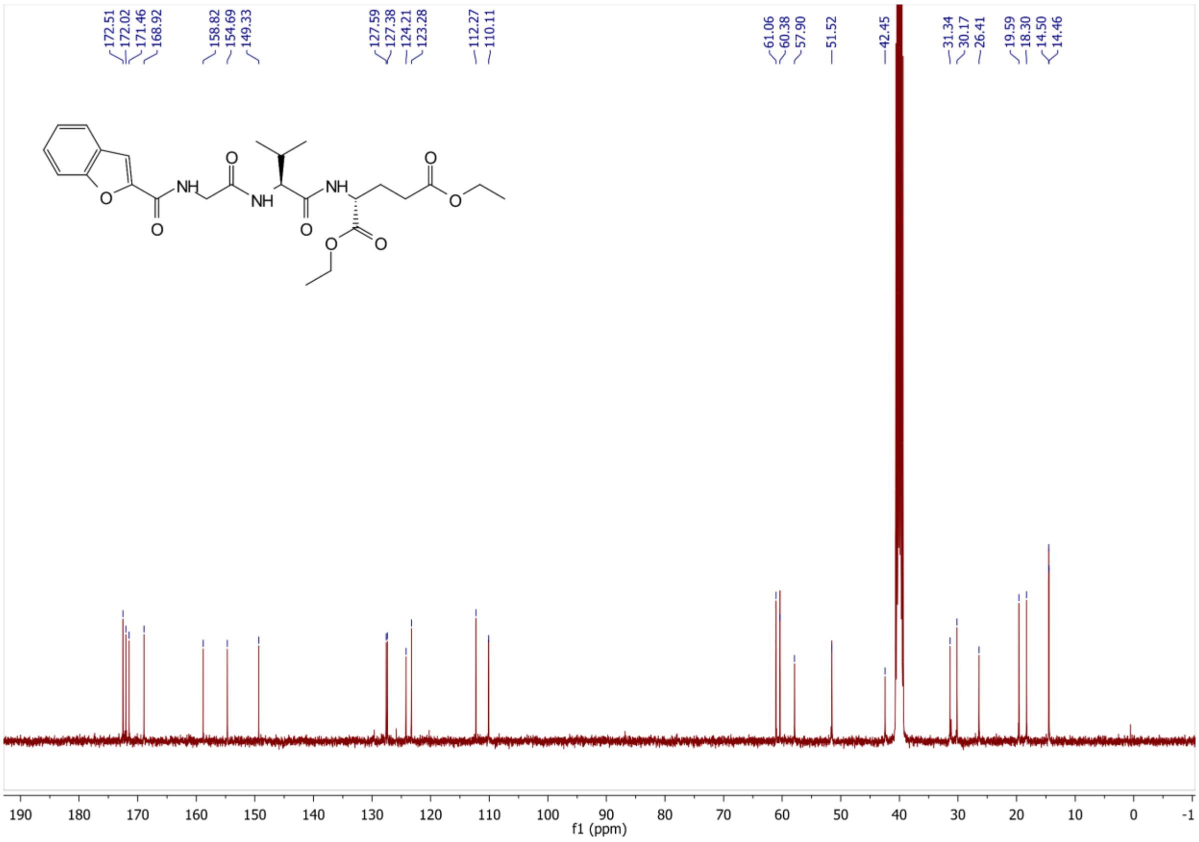

Compound **31**: ^{1}H, 400 MHz, DMSO-d_6

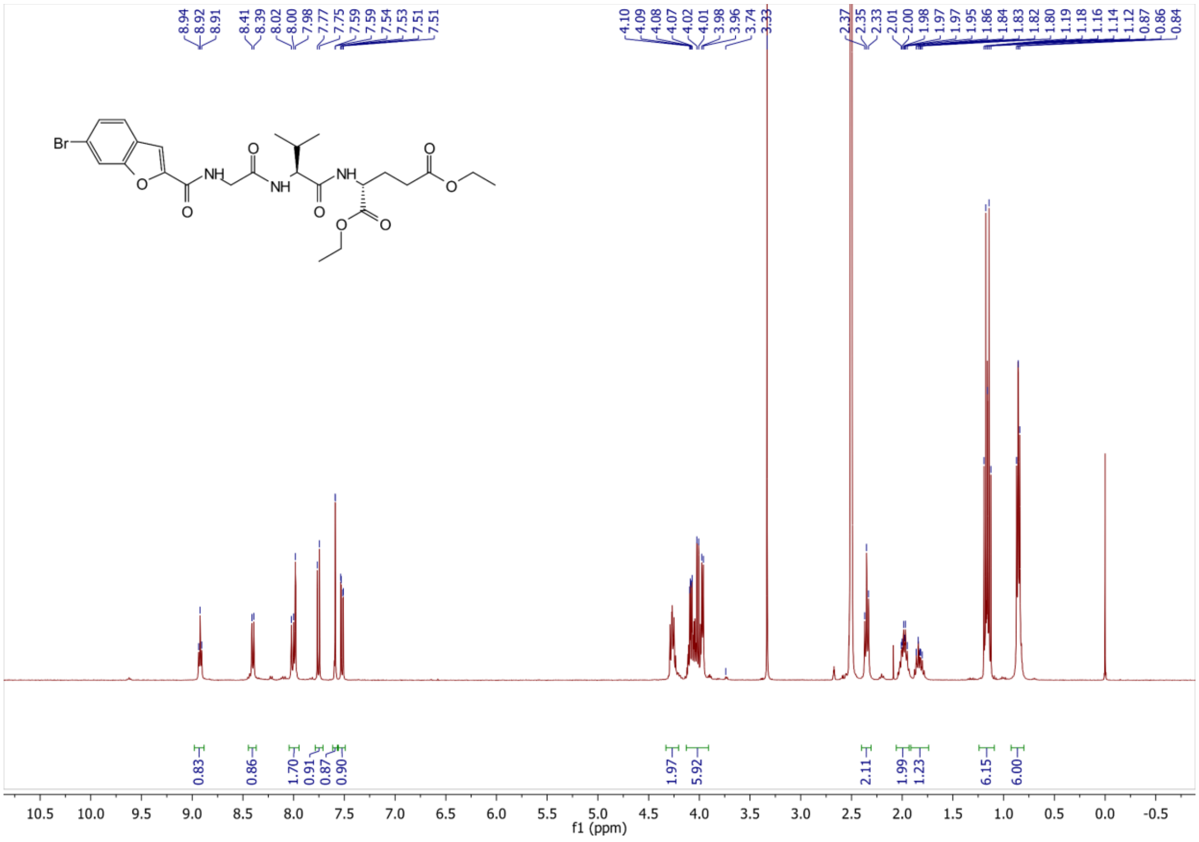

Compound **31**: ^{13}C, 101 MHz, DMSO-d_6

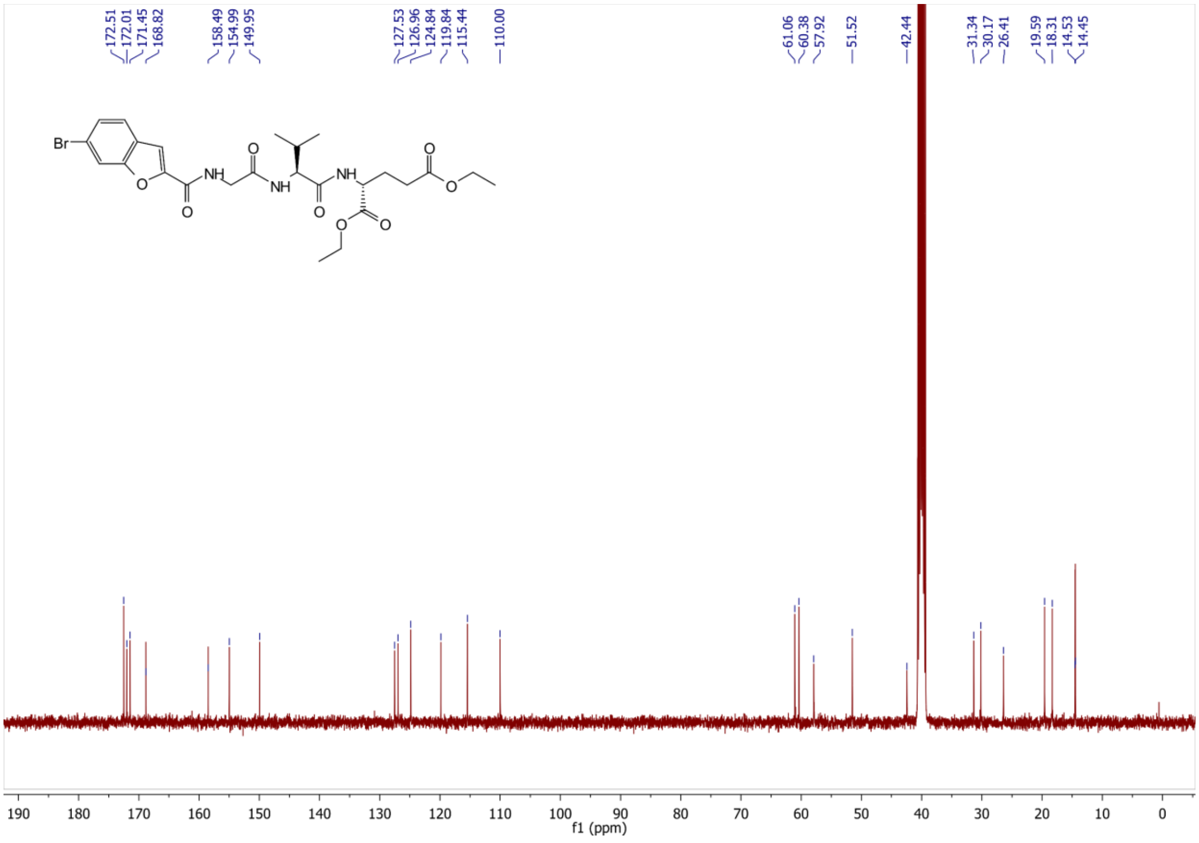

Compound **32**: ^{1}H, 400 MHz, DMSO-d_6

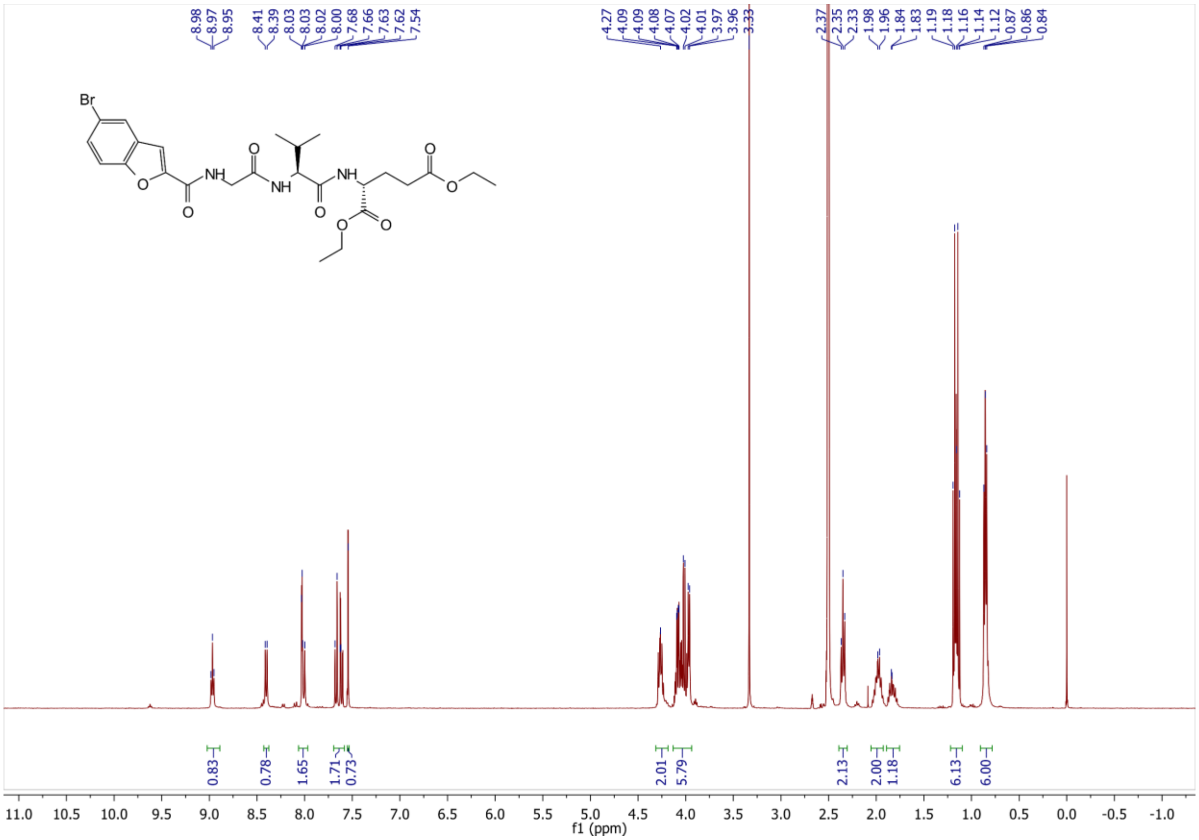

Compound **32**: ^{13}C, 101 MHz, DMSO-d_6

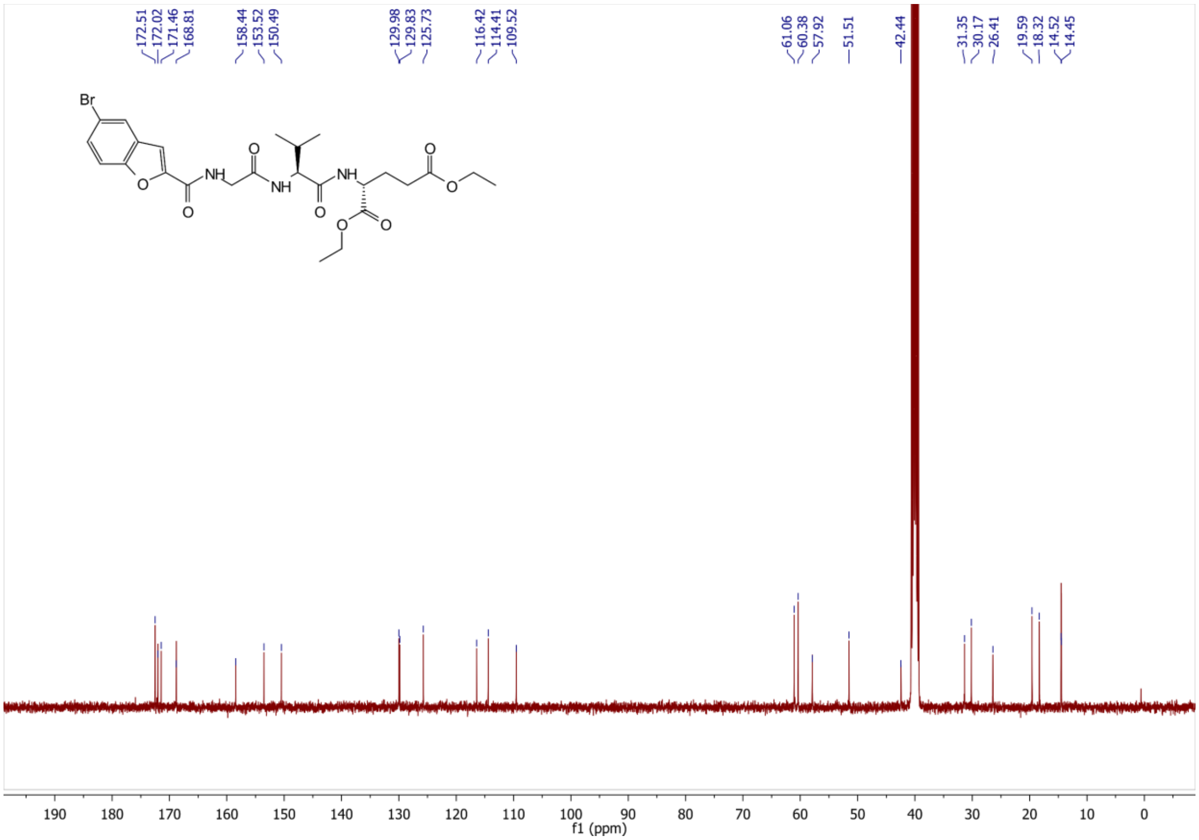

Compound **33**: ^{1}H, 400 MHz, DMSO-d_6

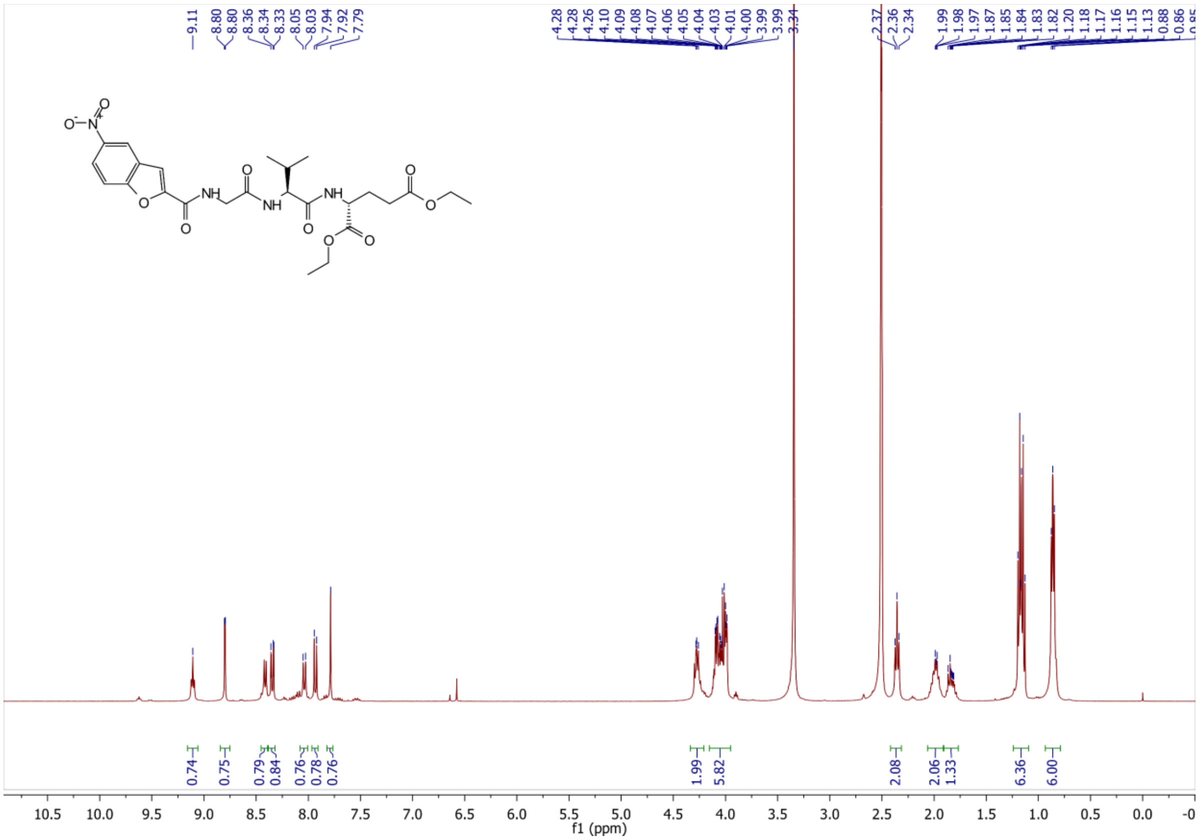

Compound **33**: ^{13}C, 101 MHz, DMSO-d_6

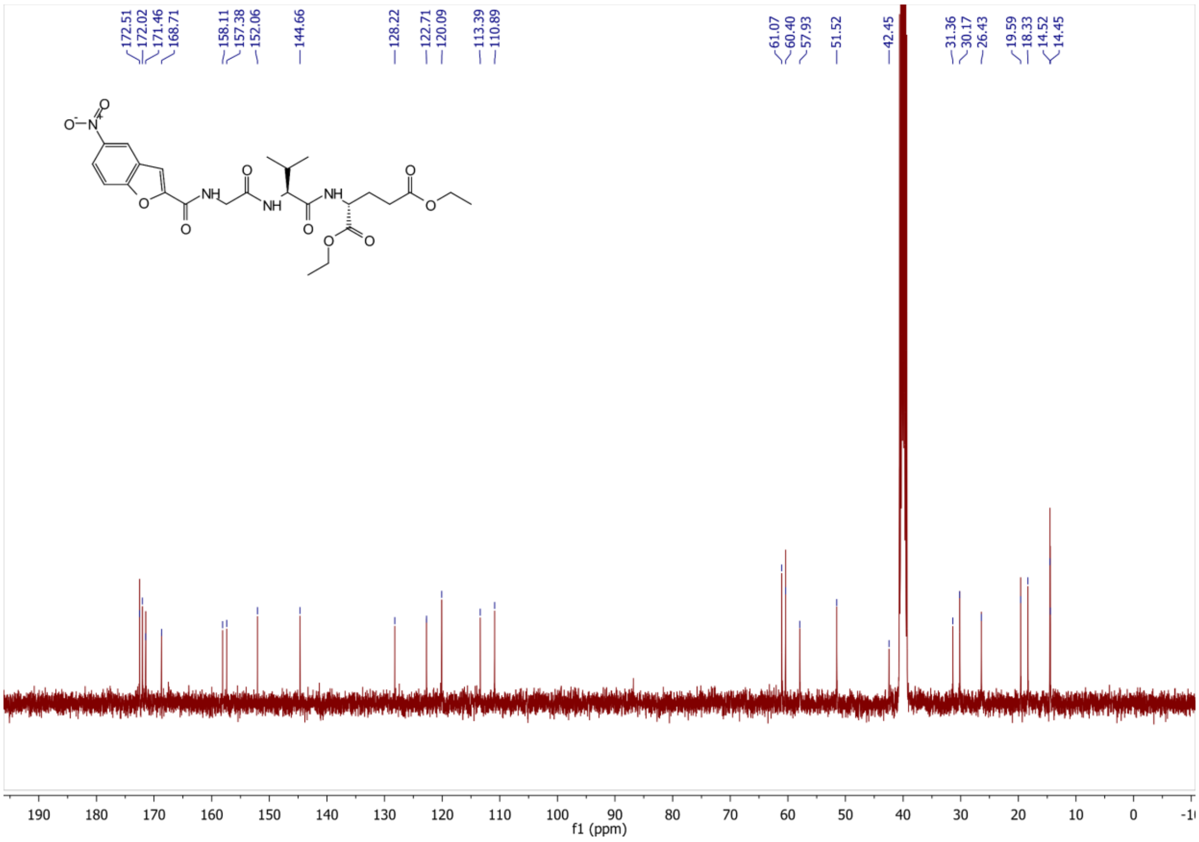

Compound **34**: ^{1}H, 400 MHz, DMSO-d_6

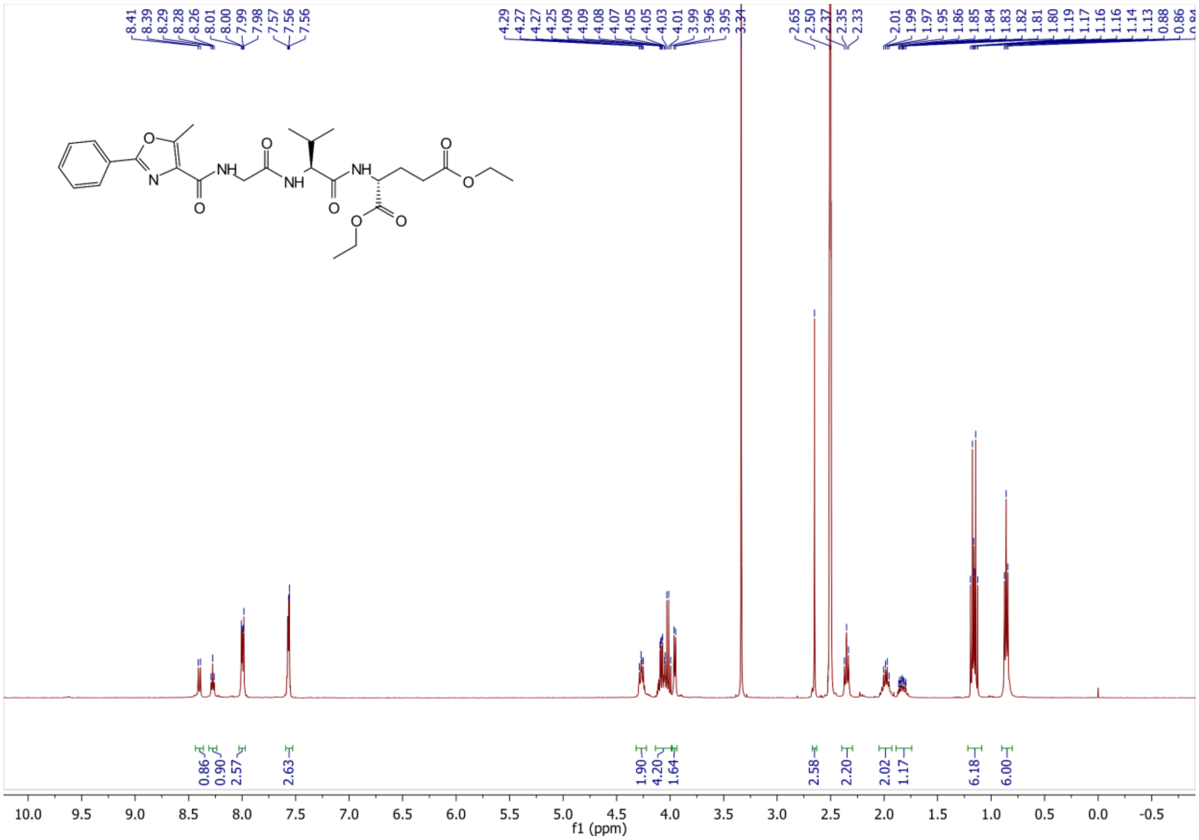

Compound **34**: ^{13}C, 101 MHz, DMSO-d_6

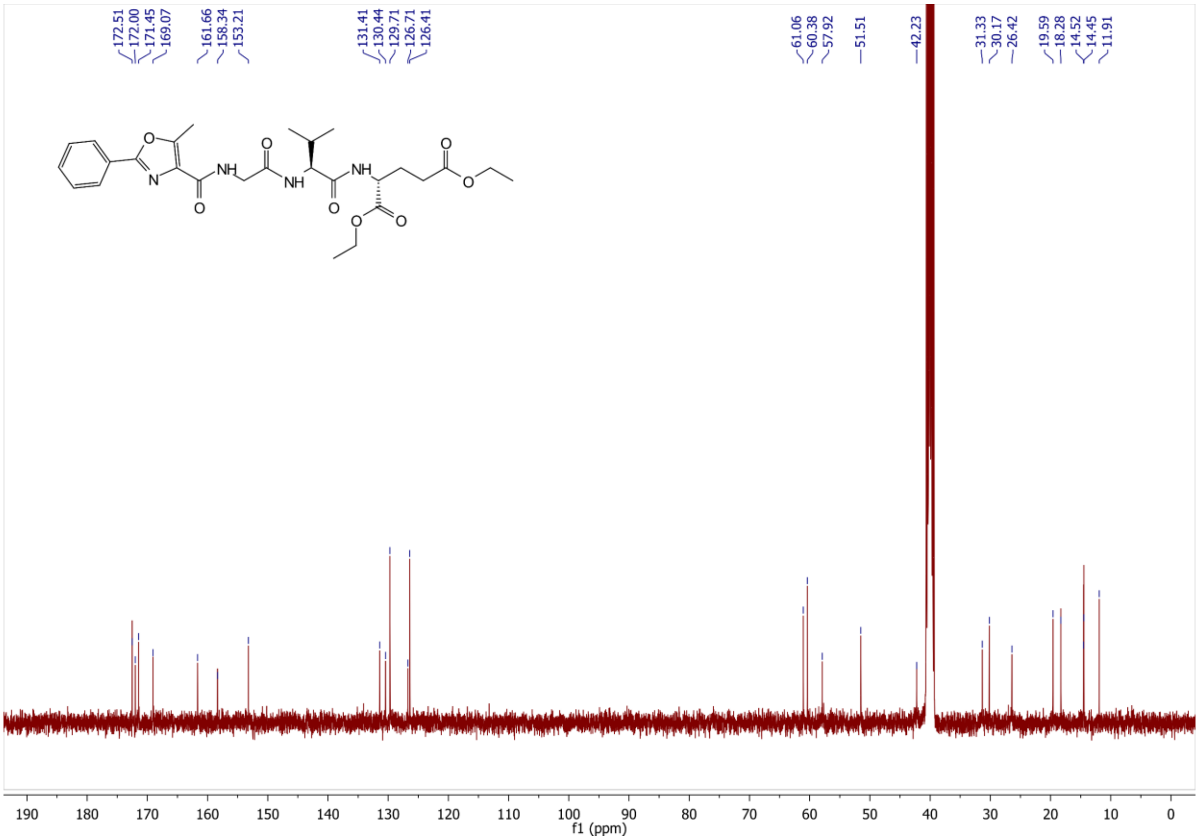

Compound **38**: ^{1}H, 400 MHz, DMSO-d_6

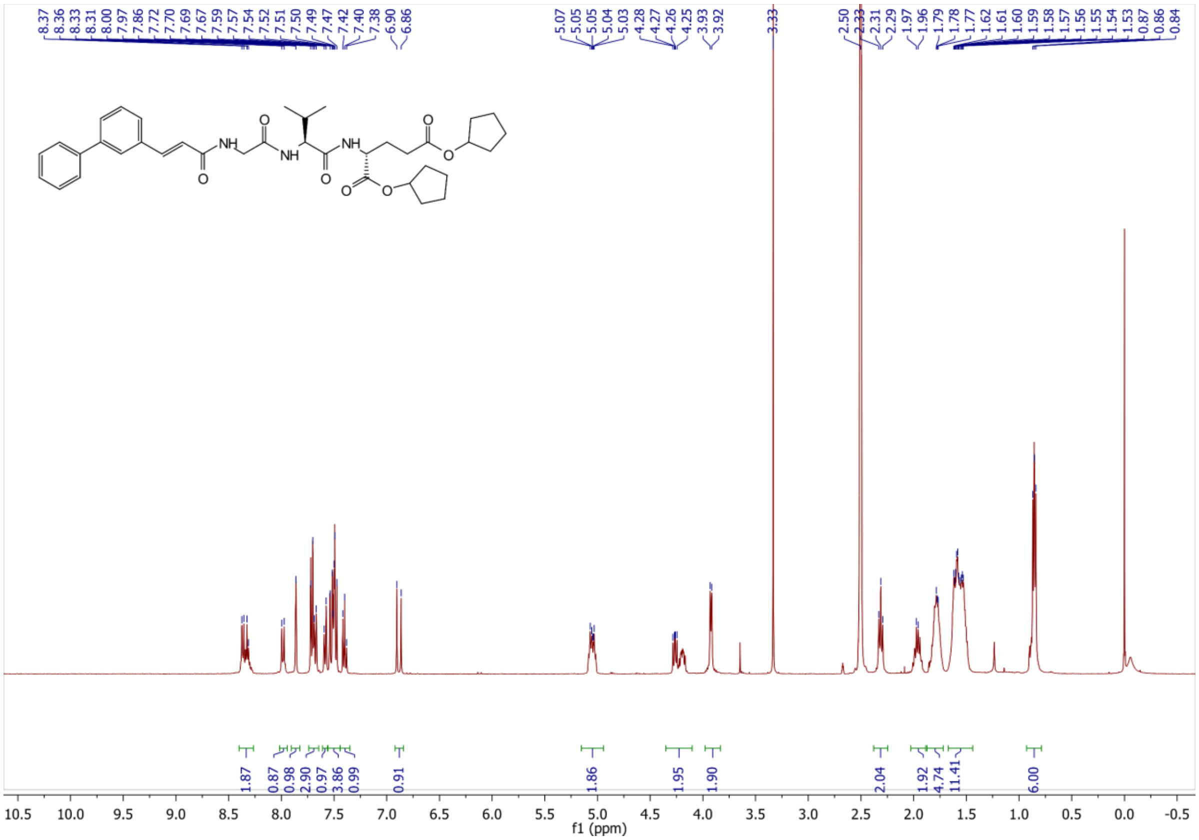

Compound **38**: ^{13}C, 101 MHz, DMSO-d_6

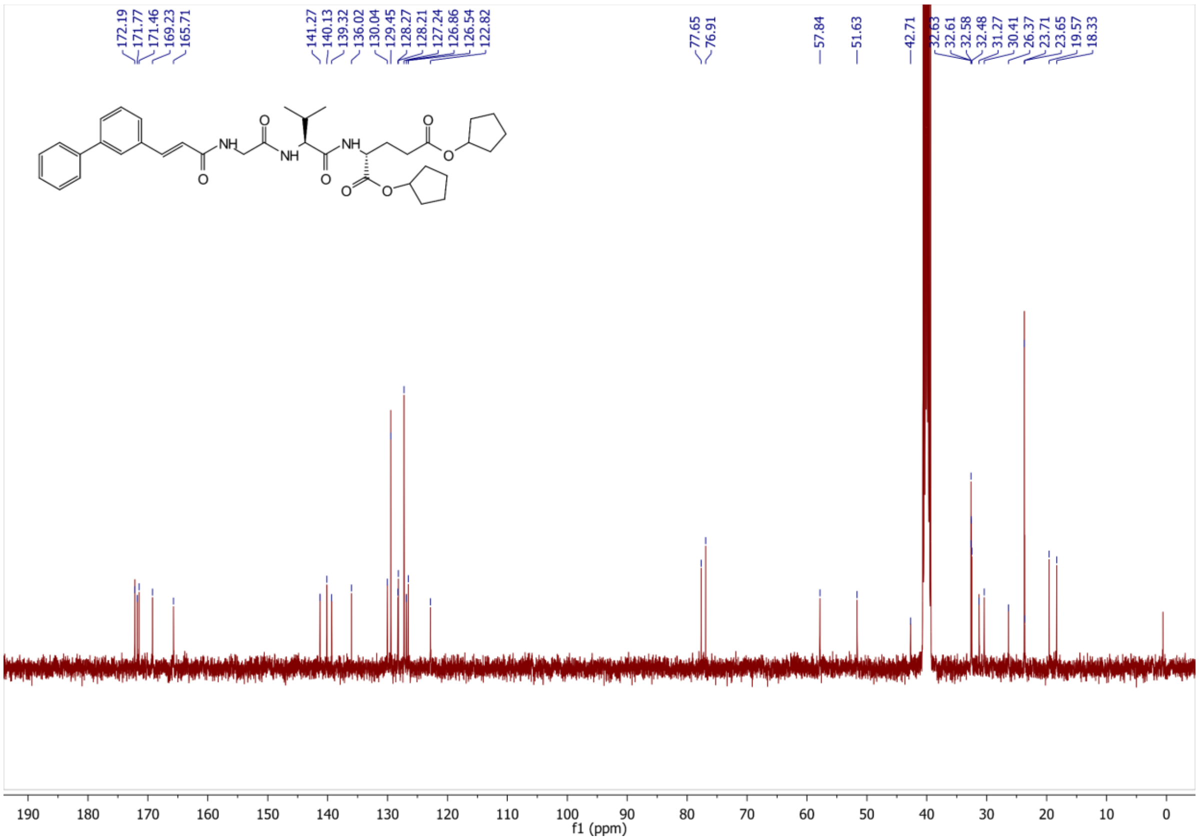

Compound **39**: ^{1}H, 400 MHz, DMSO-d_6

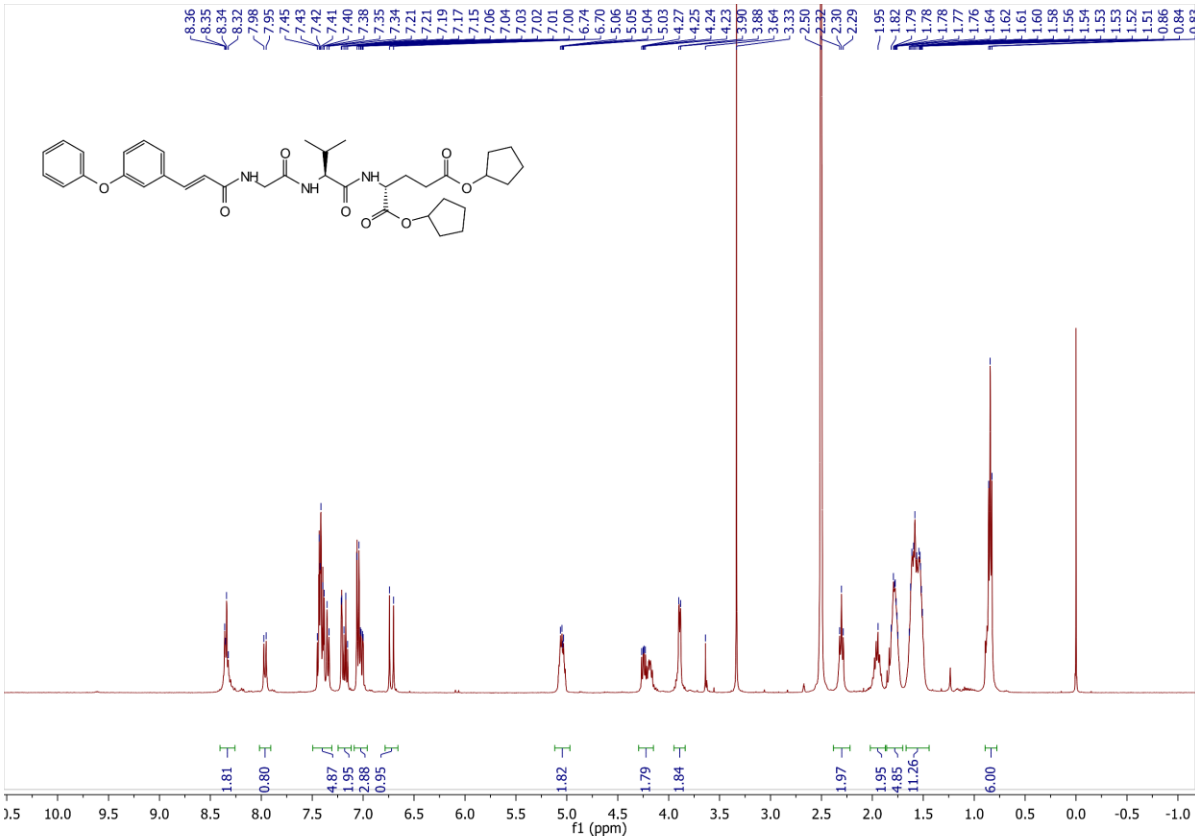

Compound **39**: ^{13}C, 101 MHz, DMSO-d_6

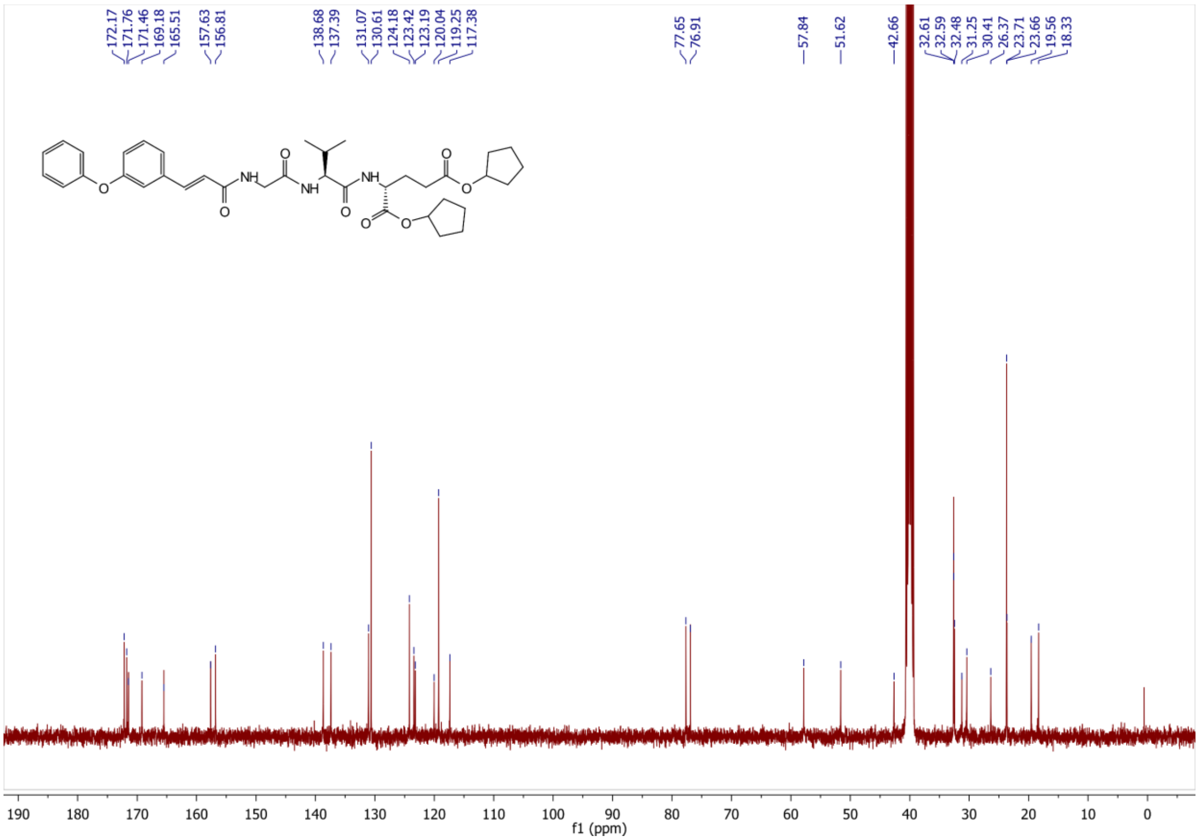

Compound **40**: ^{1}H, 400 MHz, DMSO-d_6

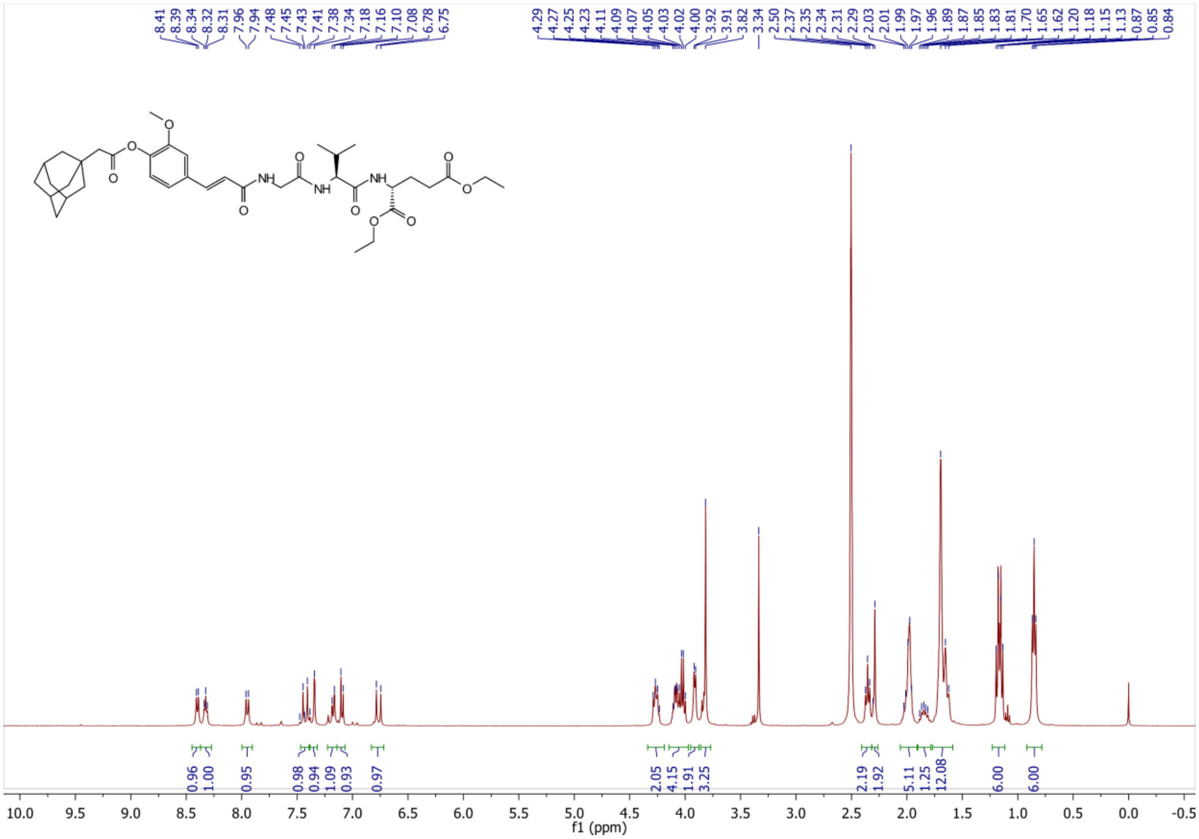

Compound **40**: ^{13}C, 101 MHz, DMSO-d_6

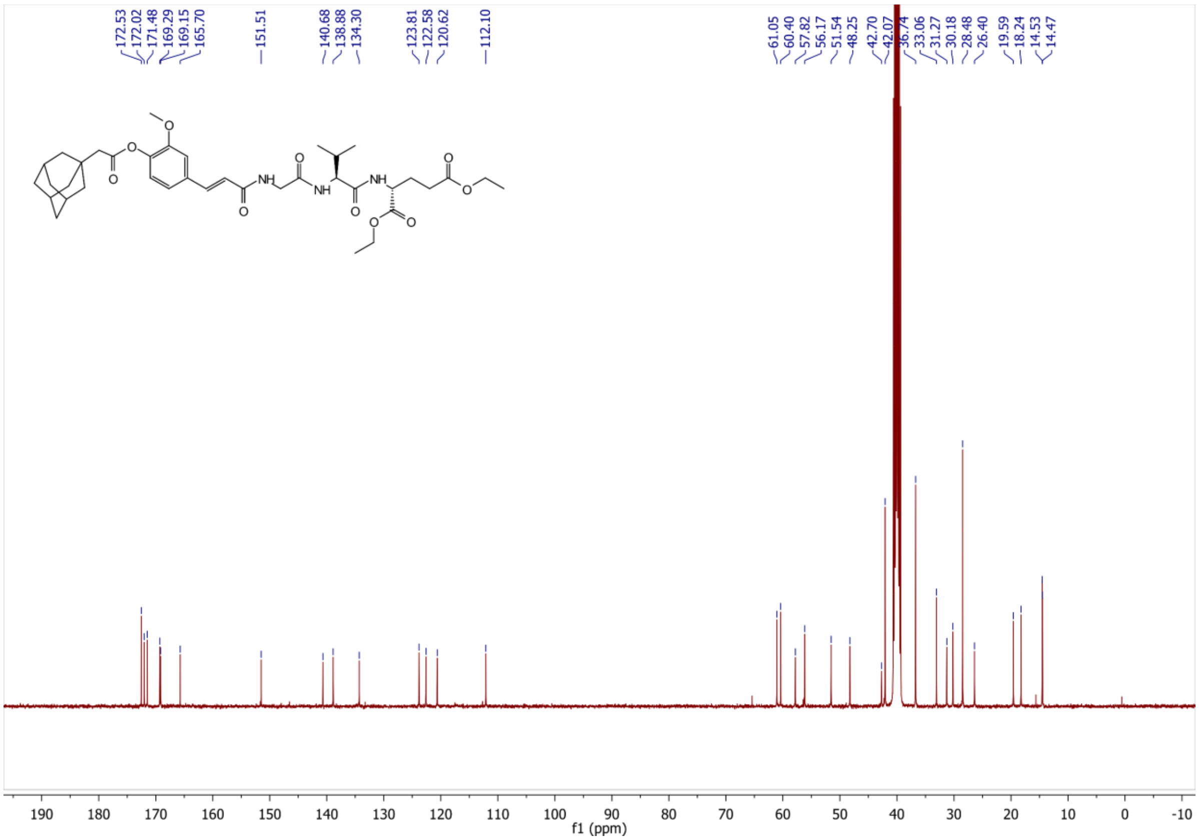

Compound **41**: ^{1}H, 400 MHz, DMSO-d_6

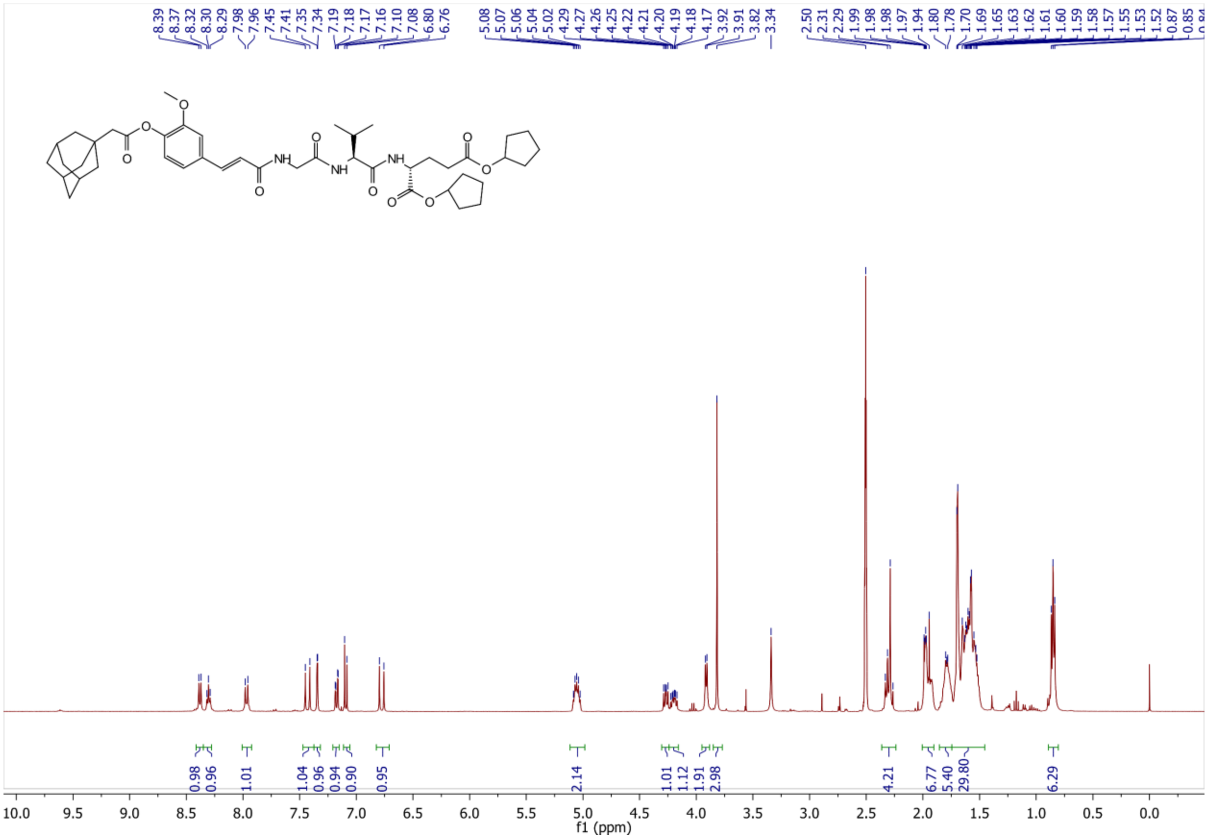

Compound **41**: ^{13}C, 100 MHz, DMSO-d_6

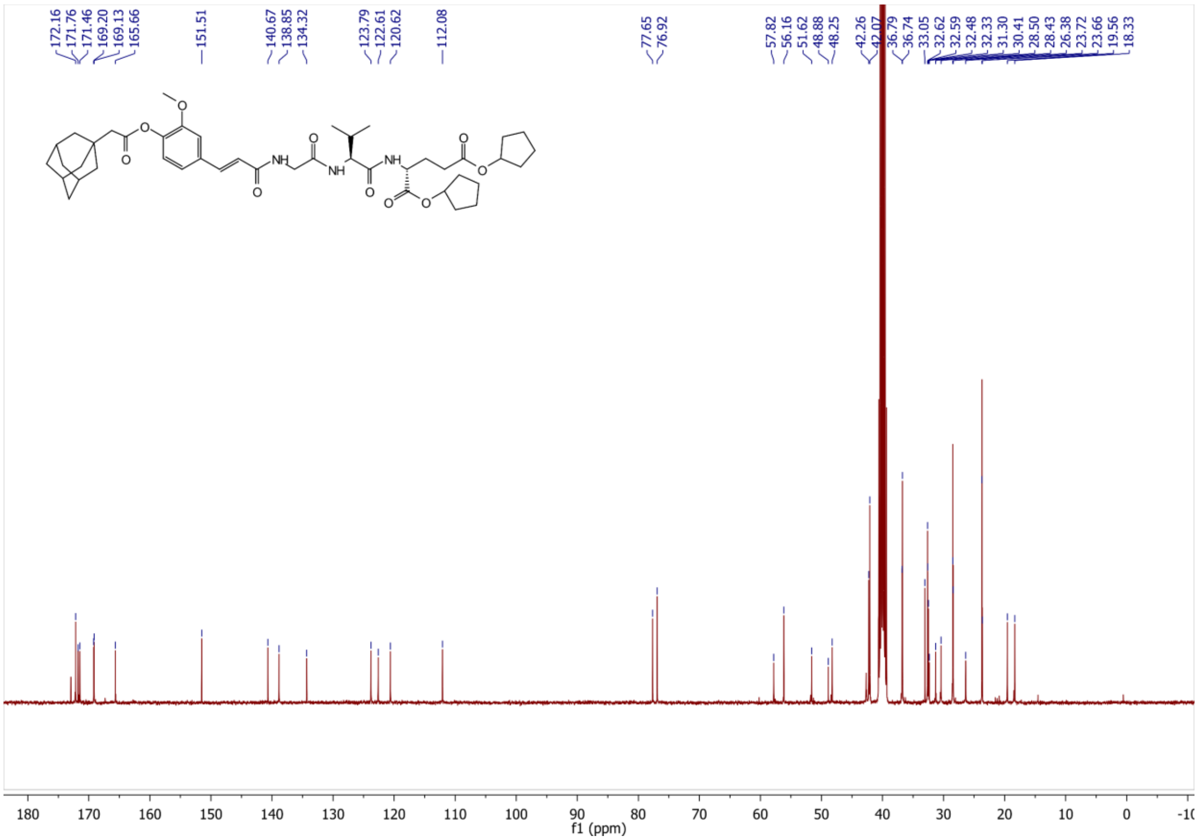

6 References

(1) Gobec, M.; Tomašič, T.; Štimac, A.; Frkanec, R.; Trontelj, J.; Anderluh, M.; Mlinarič-Raščan, I.; Jakopin, Ž. Discovery of Nanomolar Desmuramylpeptide Agonists of the Innate Immune Receptor Nucleotide-Binding Oligomerization Domain-Containing Protein 2 (NOD2) Possessing Immunostimulatory Properties. *J. Med. Chem.* **2018**, *61* (7), 2707–2724.

(2) Guzelj, S.; Nabergoj, S.; Gobec, M.; Pajk, S.; Klančič, V.; Slütter, B.; Frkanec, R.; Štimac, A.; Šket, P.; Plavec, J.; Mlinarič-Raščan, I.; Jakopin, Ž. Structural Fine-Tuning of Desmuramylpeptide NOD2 Agonists Defines Their In Vivo Adjuvant Activity. *J. Med. Chem.* **2021**, *64* (11), 7809–7838.

(3) Guzelj, S.; Gobec, M.; Urbančič, D.; Mlinarič-Raščan, I.; Corsini, E.; Jakopin, Ž. Structural Features and Functional Activities of Benzimidazoles as NOD2 Antagonists. *Eur. J. Med. Chem.* **2020**, *190*, 112089.

(4) Carmen B, L.; Bismarck B, L. Human Chronic Myelogenous Leukemia Cell-Line with Positive Philadelphia Chromosome. *Blood* **1975**, *45*, 321–334.
